# Vertical Archaeology: Safety in the Use of Ropes for Scientific Research of Pre-Columbian Andean Cultures

**DOI:** 10.3390/ijerph18073536

**Published:** 2021-03-29

**Authors:** Pedro Ignacio Saez, Elena Ángela Carrión, Encarnación García, Manuel Ollanta Aparicio-Flores

**Affiliations:** 1 Honorary Collaborator, Building Sciences & Urbanism Department, University of Alicante, 03690 Alicante, Spain; pi.saez@ua.es; 2 CRESMES, Research Group for Testing, Simulation and Modelling Structures in Civil Engineering and Architecture, Civil Engineering Department, University of Alicante, 03690 Alicante, Spain; 3 Senior Lecturer in Construction Unit, Building Sciences & Urbanism Department, University of Alicante, 03690 Alicante, Spain; 4 Arquitecto de Cusco, Perú, Especializado en Conservación del Patrimonio Cultural ARC90-ICCROM, Representante del Proyecto Ukhupacha en Perú, 04100 Rome, Italy; ollanta1@cuscoananau.com

**Keywords:** vertical archeology, ropes, vertical work, personal protective equipment against falls, harness, safety at height

## Abstract

Scientific research is sometimes subjected to go through field study in places that are difficult to access. Where man has not managed to reach through traditional techniques, work at height systems offer new possibilities, provide safety in exploration and represent an excellent tool that allows a new insight of spaces object of scientific research. For more than 20 years, the Ukhupacha team has been studying, analyzing and selecting the teams, techniques and rope progression systems that best adapt to archaeological works in vertical environments. The projects studied are developed in Pre-Columbian cultures of the Andean mountain range, the high Amazon jungle and its surroundings. As a result, a new working methodology called Vertical Archaeology has been developed. It prioritizes user’s safety by means of ropes when accessing archaeological research areas. The recommended and safest systems, techniques and personal protective equipment (PPE) are presented for each of the phases in which it is proposed to divide archaeological investigations: exploration, training and expedition. Using rope access techniques has allowed the safe study of new artistic and heritage aspects in ancient civilizations, as well as the approach of the scientific community to places that until now had remained hidden.

## 1. Introduction

**Figure 1:**
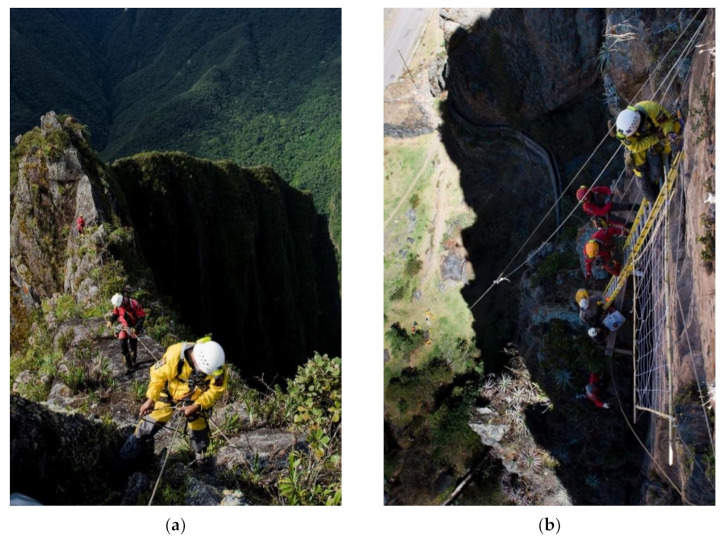
(**a**) Qhapac Ñan exploration near the Machupicchu site. (**b**) Conservation works on a cave painting of Inkapintay in Ollantaytambo.

On the eastern slope of the Andean mountain range, there are areas of great archaeological interest susceptible to scientific study [1,2,3,4,5,6]. In the Andean worldview, ancestor worship is a relevant tradition. Therefore, it is a very common practice to build both small burials or “chullpas”, as well as large entire necropolises, in vertical places at high altitude through the construction of walls of support and graves [7,8,9]. After the first Inca occupation and later, with the arrival of the Spanish, these described places and, also, other residential-type settlements such as Kuelap, which are markedly inaccessible, disappeared when abandoned. The jungle and nature reoccupied them, and so, they have remained over time and for centuries without being pinpointed, mainly due to the complexity of accessing the study areas [10]. In this context, archaeologists and researchers are forced to use complex rope access techniques in order to carry out their scientific investigations (Figure 1). In the South American continent, the Andes form the backbone of the territory. There, millenary cultures developed, those leading to the Inca magnificence. Unique works such as Machu Picchu, Choquequirao or Kuelap were built. The strategic location at a height meant, in addition to its utilitarian sense and cosmic observatories, the proper administration of the territory between its valleys. Especially, the areas of the Andean mountain and the Amazonian Andes are area with an orography characterized by great verticality. Much of the heritage interest, especially in architectural and construction manifestations, are characterized precisely by that marked verticality.

Until now, different methods have been used for the archaeological investigation of these places. However, the usual means of working at a height (stairs, scaffolding or platforms) are not safe or effective or even applicable due to the environment. Other projects have been using, in an unsafe way, sport techniques called “vertical progression techniques” [7] from mountaineering, climbing, canyoning and, mainly, speleology [11,12,13,14,15], which were used at the beginning.

Currently, it is being observed in other archaeological research projects developed in the Andean environment that sports techniques and equipment (from climbing, caving and canyoning) continue being used directly and indiscriminately. Scientists and researchers are personnel with little or no experience or training in mountain techniques or working at a height; therefore, it is essential to increase their safety by adapting these techniques to the user’s profile. A Fall Arrest Systems (FAS) consists mainly of three parts: body harness, anchor and connection system. In addition, there are other important elements and components, such as ropes, connectors, energy absorbers or anchor equipment, that are used to complete working systems at a height [16,17,18,19]. An efficient use of FAS contributes to the decrease of accidents [18]. The consequences of an accident and the probability, increased if the users are not experts and lack the necessary training, shows a risk in the assessment [17]. In addition to the risk for users, mainly that of falling, there is a non-negligible risk of damage to the cultural, archaeological and natural environment if the equipment and access techniques are used incorrectly [7].

It is remarkable that, since the nineties, worldwide legislation and regulations have been articulated and are applicable to any work carried out at a height or with the risk of falling. In recent years, an important technical evolution can be noticed in systems based on personal fall protection equipment to carry out work at height, together with the appearance of new equipment and systems, as well as the proliferation or related regulations. Nowadays, personal protective equipment appears in two different fields: labor and sports. Compliance with the safety requirements demanded by the legislation and the technical specifications applicable in different international regulations make it so that the workplace and the sports world follow similar paths but with clearly divergent techniques, equipment and systems. From a legal and regulatory standpoint, the set of American standards ANSI/ASSE Z359 [20] is known as the Fall Protection Code. They are a series of standards that, since the first edition in 1992, have progressively regulated all aspects of these systems [20]. Later, in Europe in 2008, with the appearance of the EN 363 standard [21], a new classification was established that described, detailed and standardized the five Fall Protection Systems (FPS): restraint systems (RS), fall arrest systems (FAS), work positioning (WP), rope access systems (RAS) and rescue systems (RES).

The equipment used in the world of work has the great advantage of meeting the high safety requirements. However, its main drawback is the weight and volume of the PPE used. Their transfer and mobilization in this vertical environment are very complicated and, sometimes, not even feasible.

The project’s objective is to develop a safe and effective method based on techniques of working at a height with ropes to facilitate the study of these new archaeological discoveries. In the last 20 years, an important technological advance in work systems and equipment has been produced to facilitate access and study to these places safely. The Ukhupacha Project [22] is made up of a multidisciplinary team that are experts in rope access techniques who have developed several projects in Andean territory. Such a team has conducted more than 20 expeditions in the area. The team was created in 1997, when a group of speleologists, climbers, university students and firefighters, all of them with the mountain as a common interest, came together around a central idea: to make accessible and give visibility to places that the scientific community had not accessed so far. For the last two decades, this project has been developing the method call Vertical Archaeology.

In the development of several projects, the installation and use criteria were established, as well as the use of FAS; the researchers used PPE safety conditions, and the knowledge for the management of the necessary equipment and techniques was transmitted. This made it possible for users to reach the areas of study with a minimal risk. From a safety viewpoint, no height falling accident has been registered for more than two decades of projects and investigations. The most remarkable accident has been a sprained ankle while walking on the trail.

**Figure 2:**
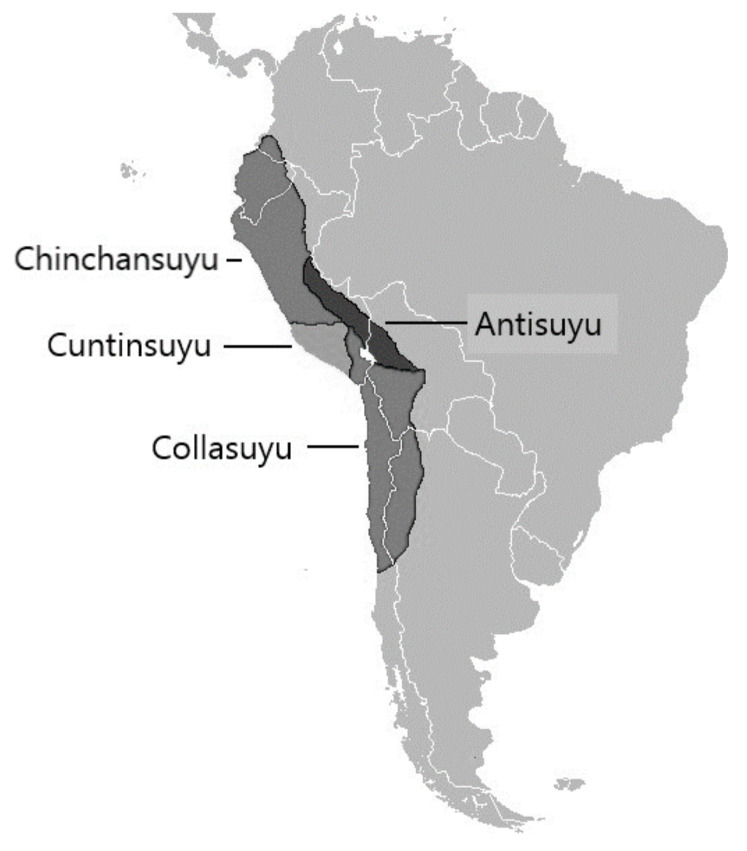
Geographical localization where these techniques have been applied.

The use of rope access techniques is a very important step that represents a great advance to guarantee the safety of researchers in research projects. For the first time, the adaptation of sports techniques is proposed, together with the main contributions, in terms of safety in the world of work and falls arrest systems. As Figure 2 shows, the main zones of archaeological intervention of the Ukhupacha Project were developed within the geographical area historically known during the Inca time as Tahuantinsuyu. In Quechua, it means the state of four regions: Chinchaysuyu, Antisuyu, Collasuyu and Cuntinsuyu. It is part of the Inca culture, with a universal heritage value.

Qhapac Ñan, also known as Inca path, was the most remarkable civil work that enabled the development of the empire. It is a road network that communicated all the territories of Tahuantinsuyu, with more than 30,000 km for the Peruvian territory and, in its ex-tension through the South American continent [23], reaching 60,000 km [24]. Its construction in inaccessible high-altitude places and its marked vertical development make it one of the main objectives of the investigations and explorations carried out by the Ukhupacha Project [25,26]. 

This work indicates the great relevance of the heritage to be studied in this vertical environment and the great difficulty in accessing it. In rope access techniques, an adaptation of all these systems, techniques and equipment of sports and labor areas is crucial to guarantee two aspects: first, the appropriate safety standards for nonexpert personnel such as scientists and researchers, and second, the preservation and the respect of heritage by the appropriate use of the techniques and materials applied.

## 2. Materials and Methods

This section exposes how the selection of the different suitable materials, equipment, systems and methods for Vertical Archaeology has been conducted.

### 2.1. Materials

Part of the study of the features and performances of PPE available on the market [17,27,28,29,30,31,32]. There is a wide variety of PPE, which provide solutions for several activities, for example, within the working sphere (pruning, power lines, posts, fall arrest systems, vertical Works, etc.), as well as sports sphere (climbing, speleology, mountaineering, canyoning, etc.). Analyzed, studied and used more than a thousand pieces of equipment, the most versatile and safe ones selected for Vertical Archaeology works. In this paper, Section 3 exposes the PPE resulting from this research and its main features. Ukhupacha Project has always taken into account technical innovation. It has always been in direct contact with the main manufacturers of new equipment to be able to analyze and test them in the Andean area and, thus, determine if they are appropriate for this complicated environment.

The different materials and the equipment used in the projects are detailed in Table 1. From sports PPE used by specialists, PPE from the working world have been included for being used by researchers (with little training in height works).

The technical members of the Ukhupacha team develop their professional lives linked to the use of these PPE coming from different professional fields, such as cavers, climbers, firefighters, rescuers from mountain rescue groups, PPE manufacturing companies and safety engineers committed to the selection of PPE.

All of them are permanently informed and are testing the latest news and developments in the complex field of PPE. If a new product is detected in the market, it is communicated to the team, which immediately studies its suitability for archaeological research. Likewise, the different theoretical and practical possibilities of application are analyzed. After that, a meeting of the team members is held so as to determine, with the information provided by the manufacturer and the practical tests carried out, if such PPE is compatible and in which manner it can be incorporated into the usual work. 

**Figure 3:**
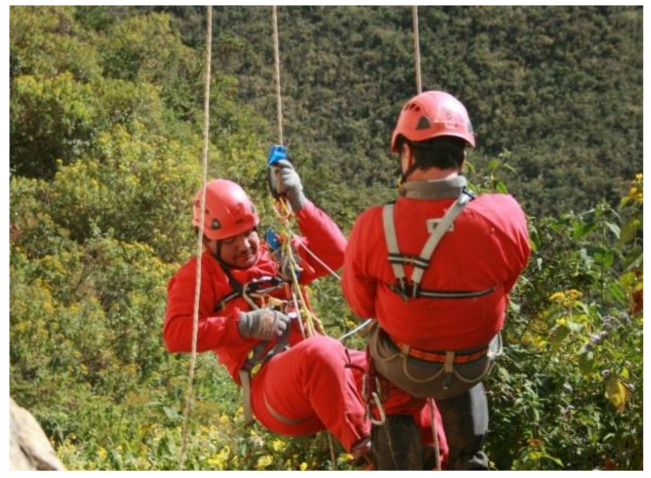
Archaeologist speleology training, workmate rescue/retrieval appropriate to his level, Leymebamba (Peru, 2013).

Once its suitability has been approved, it is first put into practice by the Ukhupacha team of vertical specialist technicians. If the result is satisfactory, this PPE is made available for training to scientists and researchers of a more inexperienced profile (Figure 3). Finally, it is assessed whether the equipment is useful, comfortable and fulfils its functionality.

### 2.2. Methods

The Ukhupacha technical team has studied more than 30 different methods, systems and techniques based on vertical rope access [7,17,18,19,27,28,29,45,46,47,48]. It is composed by highly experienced rope access specialists who come from different fields of work, including rescuing and rescue services for professional fire brigades [27], federation mountain rescue groups, helicopter rescue services and health specialists; in some cases, these members hold the title of head of the aforementioned professional services. The results and conclusions of the Emergency Plan section are based mainly on all this extensive experience in more than a thousand mountain rescues accumulated by the whole team.

Besides that, some members work in the training of those groups and keep a direct business relationship with the most important worldwide manufacturers of personal protective equipment [31,32]. This fact has allowed to obtain first-hand information and to test certain prototypes before even going to the market. They meet 3 to 4 times a year with the objective, among other issues, of evaluating the appearance of new PPE, considering the opinion and experience from different points of view (labor and sports) and carrying out the proper selection of PPE needed for the environment studied in this article. 

Finally, other people have a university academic profile—among which, there are architects, engineers or higher specialists in the safety and prevention of occupational risks. They normally work in construction, public and civil works or mining sectors. They are used to the development of singular works and even some of them in the Andean environment with great development in heights. Their knowledge on works, legislation related to these techniques, equipment and systems has been found fundamental to legally justify the proposed method. Particularly, they have been essential at the time of choosing redundant safety systems such as double rope or the incorporation of safety PPE like fall arrest harness, energy absorbers and guided type fall arresters. This equipment is not used in sports, but they are crucial to guarantee researchers’ safety requirements.

The methodology is based on the different meetings of the technical team. There are three types of meetings: annual with all the members, semiannual when there is a need to take other types of more urgent decisions and telematic ones to finalize the details. During the first meetings, an initial risk assessment and appropriate preventive measures were carried out, mainly to control the risks of falls from heights. The need to establish the three different phases in all the projects was concluded (exploration, training and expedition). The first remark was that the technical team of specialists has the highest knowledge and skill in rope access and rescuing, while the team of researchers and scientists do not have any experience or training. In the case of the latter group, errors are more likely, and therefore, redundant safety techniques are required. The minimum contents of the annual meetings that affect each project are: election of the director or person in charge, temporary planning, number of members who participate in the work and specific functions of each one, techniques and PPE for the Ukhupacha technical team, light techniques for recognition and advancement, training techniques and PPE in a controlled environment, redundant safety techniques for students (archaeologists and other scientists), emergency plans, proposed improvement of new maneuvers and systems and the analysis of new equipment on the market. 

Feedback: there is a constant update of the method since during the different phases of each project; field notes, relevant data and observations are taken. Once finished, a report is developed [22] in which the totality of relevant data is consigned in order to be used in future actions; the results obtained and specific aspects such as the suitability of the PPE are also included and advantages and disadvantages are analyzed, as well as problems that have appeared and everything that directly affects the Vertical Archaeology techniques used. Likewise, telematic meetings are held to solve urgent questions if necessary. In the case of a discrepancy at the time of selecting a specific PPE or technique to be applied, the most restrictive criterion from a safety point of view is adopted. Every new project takes the memory of the last project as its starting point.

Prior to any field activity and vertical archaeology research project, it is essential to draw up an Emergency Plan that includes directives for immediate help in the event of an accident. Similarly, you must have the necessary permits and administrative procedures for the start of activities. The authorization not only by the central administrations of the corresponding governments and ministries is also essential that the communities and populations closest to the deposit know, get involved and authorize the project. Not having previously considered these two safety premises before, the work can be the origin of many later problems, including the failure of the project.

Eventually, after having selected the appropriate PPE, having studied and analyzed the different techniques and systems, it is all applied in the fieldwork. The developed method establishes three perfectly differentiated phases as for PPE and employed techniques: (1) the exploration phase, with experts in heights, where the techniques used mainly belong to sports. Lightness and versatility prevail, in order to be able to locate the sites; (2) the training phase, in which methods are redundant in safety and a controlled environment (base camp), until researchers acquire certain skills, and (3) the expedition phase, in which capable researchers and height technicians go to the sites so as to guarantee the safety of researchers at all times. In this last phase, techniques used with ropes are of redundant safety, but the environment is not so controlled anymore. This taxonomy of equipment, techniques and systems in three differentiated phases has not been used previously in similar research projects, assuming a notable advance in the safety of researchers.

## 3. Results and Discussion

The direct application of techniques and PPE from sports, such as climbing or speleology, without a prior analysis of suitability means a serious error that can lead to negative and even fatal consequences for researchers. The transposition of these techniques requires a detailed study by experienced specialists and a process of technical adaptation of the equipment and systems, which are adapted to the profile of the inexperienced user to whom they are addressed. The vast experienced throughout all these projects allowed us to observe notable differences that must be kept in mind and stand out among the area of sports with respect to the specific techniques applied in Vertical Archaeology. These differences are summarized in Table 2.

The main results obtained after years of development, testing and research within the Ukhupacha Project. The main safety and protection measures required in Vertical Archaeology works stand out.

First, the methodology and systems to be used are described for each of the three phases: exploration, training and expedition. In a second section, the materials, techniques and suitable PPE for the work to be carried out are discussed. They are detailed by sections highlighting the most significant of each of the PPE and materials used. Finally, in the third section, the need to establish an emergency plan prior to the start of any activity will be discussed.

As a result of the application of this methodology called Vertical Archaeology, the Ukhupacha Project has managed to carry out more than twenty expeditions, helping different publications from universities and researchers. Table 3 shows the breakdown of the suspended work time accumulated by the experts in all the projects developed. The number of people trained by the Ukhupacha Project has been 65 scientists–researchers and a total of 16 technicians for working at heights. The number of hours of daily work is eight h, of which suspended work (it is understood suspension is when someone is hanging from a rope) corresponds to an average of 50% of the total hours worked.

In more than 20 years of experience of the Ukhupacha Project, only two accidents have been registered, both during the walk to access the verticals. One of them was due to an ankle sprain, and the other was caused by a fall on an embankment when part of a road collapsed. For all the above, it can be concluded that rope access techniques, correctly applied under the supervision of experts, is a very safe work technique, since not a single accident has been found during the work in suspension.

Safety and risk prevention in work at heights are essential. Above all, researchers who carry out their interventions safely and effectively are committed to it. Using appropriate individual protection systems is a first step to achieve this.

### 3.1. Vertical Archaeology Methodology

**Figure 4:**
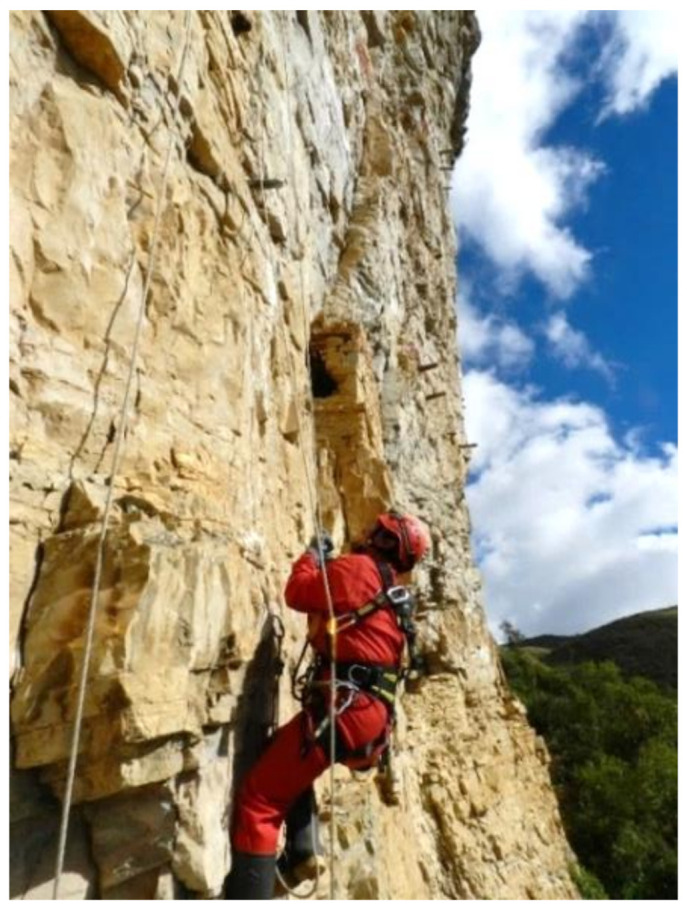
Archaeologist from the Peruvian Ministry of Culture using Rope Access Systems.

The methodology has been divided into three well-distinguished areas—namely, exploration, training and expedition. During training and expedition, the methodology must respect the safety requirements established in labor legislation and regulation. The main system used for access and positioning is the Rope Access Systems (RAS) [21]. As Figure 4 shows, the most notable feature of this system is that it has a double rope. A work rope with a double function: it prevents falls as it is used for progression and access to the workplace and a second safety rope in case of a first rope failure.

In what follows, the three proposed phases for this new method will be explained: exploration, training and expedition.

#### 3.1.1. Exploration Phase

**Figure 5:**
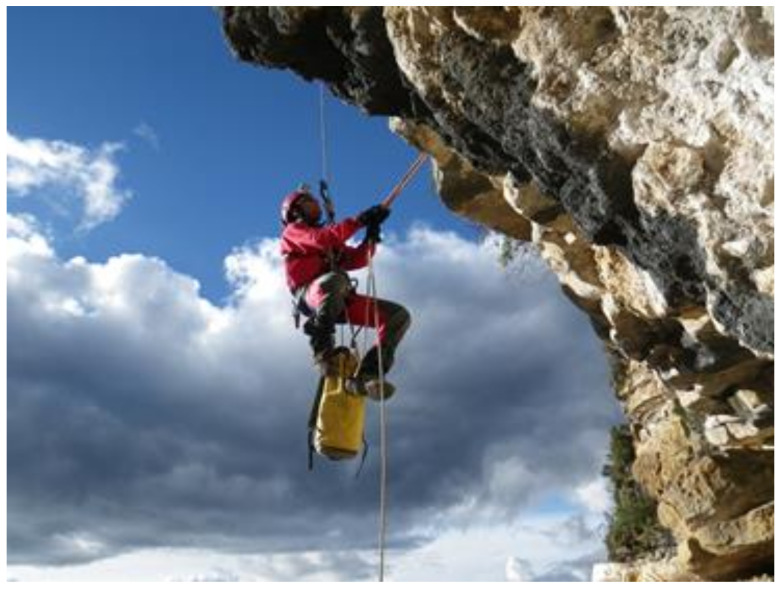
Exploration phase: accessing the city of Kuelap.

In the exploration phase (Figure 5), all details related to safety, risk, prevention, accesses, anchors and installations are analyzed and studied in depth. This first stage includes the study of the characteristics of rope installations, energy absorbers and other personal equipment, as well as the diameters, lengths of the cables and other equipment for collective use. Hence, the most appropriate ones are selected for the safest accomplishment of the expedition phase. In this phase, only the specialists of the Ukhupacha Project team perform immersion in the environment. Technicians experienced in these techniques, equipment and systems have the mission of determining the most feasible route for the research team to the different points of archaeological or scientific interest. The difficulties of the route are analyzed and documented to reproduce them in a controlled environment during the training phase. Some of the techniques used in the exploration phase have their origin in the French school of speleology [49,50]. From there on, the members of the Ukhupacha Project over the years have developed specific working methods that have been translated into guides [47,51] that also include the appropriate material and PPE.

**Figure 6:**
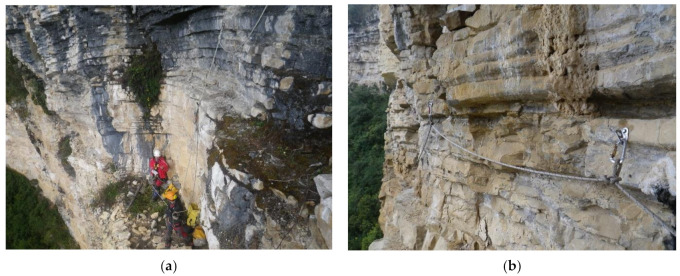
Instances of vertical and horizontal rope installation. (**a**) Installation of vertical ropes, and (**b**) handrails to facilitate safe access for researchers.

Only expert users, experienced specialists in these systems, techniques and equipment carry out the prospecting of the research areas, which allows the use of sports equipment and techniques, as well as faster progress in situ [47]. As Figure 6a,b shows, the area is studied; the different approach routes are analyzed and the rope progression installations is prepared (anchors, headlands, handrails and ropes). All this effort will make arrival and access easier for the multidisciplinary team of researchers (archaeologists, biologists, geologists, etc.). The use of sports techniques is imposed by the need to open paths that are impossible to do with redundant safety labor techniques. The success of the entire project depends on this phase, and it is the time of greatest uncertainty. Thanks to the profile of specialists, the equipment and materials at this stage are much lighter and less numerous. Likewise, it is used to carry a large part of the necessary material for the later phases (see Table 1 of Section 2, Materials and Methods). 

Another relevant aspect is the search for the suitable location for a base camp. It should be a place as close as possible to the archaeological site, always considering the safety of the participants: weather conditions, dangers due to snowfall or landslides, local fauna, availability of water and supplies, response of local communities and all the circumstances that may influence the safe development of the project. The Emergency Plan and all the necessary material for a possible contingency must be planned from the beginning of the work, even in this first phase.

The qualification and specialization of the members of the technical team is another factor to take into account. It must be studied in depth by those responsible for the safety of the project. It is essential to have a multidisciplinary team. It must include at least health workers (doctor or nurse), mountain rescuers, engineers and experts in safety and prevention of occupational risks. It is necessary that all of them have in common some experience in mountain sports.

**Figure 7:**
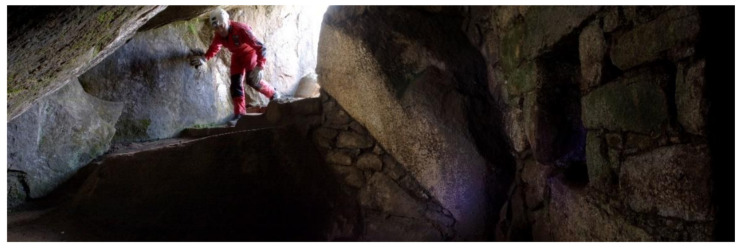
Exploration phase: an expert from Ukhupacha exploring Chinkana near Machupicchu.

This exploration phase normally lasts between 7 and 14 days. Before starting any field or research project, it is essential to have the required permissions and administrative procedures to start the activity. The authorization must not only come from the central administrations of the corresponding governments and ministries, but it is also equally important to have considered the cooperation and participation of local communities, which are true advocates of local heritage and have a wide knowledge about the environment and its specific problems. Not taking these two premises into account can lead to numerous subsequent safety problems, even the stoppage of work. Despite their lack of accessibility, difficulties in transportation and communications, the areas that have had greater social contact have been subjected to multiple archaeological looting [52]. Fortunately, a large part of this heritage has been preserved over the years thanks, on the one hand, to the ignorance of its existence, and on the other, to the need to use complex rope access techniques to gain access (Figure 7).

#### 3.1.2. Training Phase

**Figure 8:**
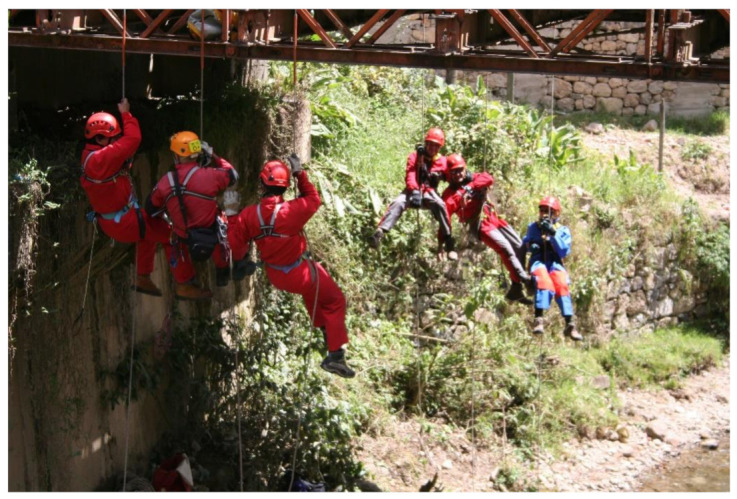
Training for researchers camp in the Amazonas Department, Peru.

The training phase includes the arrival of scientists and researchers to the area where it is stipulated to carry out the theoretical and practical classes necessary to train them in the techniques of access and positioning with ropes. In this way, they acquire the basic notions to later function safely and with a certain degree of ease in the research sites. In this controlled environment, the maneuvers that the researcher will have to perform later in the expedition phase are reproduced. Many projects are easily located in environments with altitudes greater than 3500 m above sea level. Therefore, it is vital to acclimatize the place correctly, taking into account possible problems derived from the altitude. The training has a double objective: on the one hand, to acquire a certain essential physical shape and, on the other, to train the researcher for safe progression by rope by organizing various learning activities (Figure 8). These techniques are quite complex and need periodic and regular training before each expedition. RAS management and, especially, self-healing techniques are crucial and require a certain degree of dexterity that can only be achieved with regular practice.

The systems and equipment used with researchers are more technically demanding from the point of view of safety, since they are not experienced users. For that reason, the double-rope system is used, which entails redundant safety. In essence, the safety and prevention requirements of work at height for the world of labor are strictly contemplated. This phase is fundamental, and the success of the project is due to a proper previous training of these users. In this phase, a filtering process is carried out. Those people who do not meet the necessary physical and psychological requirements are not allowed to participate in the last phase of the project, since they can put themselves and the rest of the team at risk. To this end, a series of activities are scheduled where individual technical preparation, physical condition and teamwork capacity are evaluated.

#### 3.1.3. Expedition Phase

**Figure 9:**
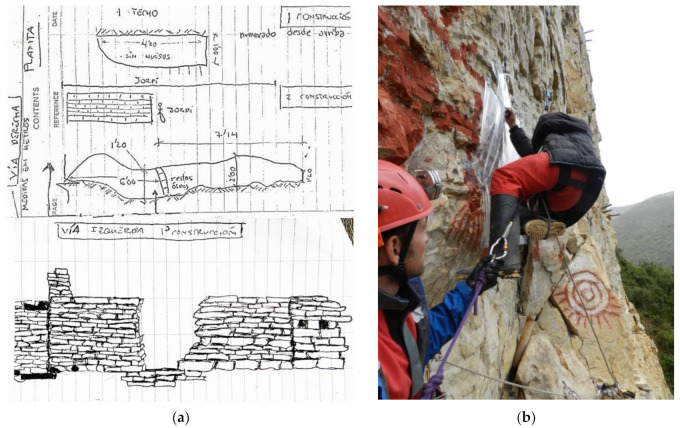
(**a**) Data collection notebook with details on the chullpas and masonry walls in burials in Chachapuyas culture, and (**b**) Researchers’ safety control.

Finally, the expedition phase consists of the culmination of the research project, an onsite testing for all the analyzed techniques, installations and equipment that have been prepared and practiced in phases 1 and 2. The acquired knowledge is put into practice, and the programmed scientific research works, sampling, sketches (Figure 9a) and other relevant data for the research are carried out. Experts in rope progression techniques and safety officers accompany the team at all times and ensure the safety of researchers (Figure 9b). To do this, the specialist explains step-by-step how they should tackle the different critical points of the site approach installation. In this last phase, safety requirements from the world of labor are used. PhD. Marla Toyne from the Department of Anthropology, University of Central Florida explains her experience in Ukhupacha Project and the mastered techniques, as well as the archaeological achievements (Figure 9a,b) she could reach thanks to the use of rope access techniques [7,53].

**Figure 10:**
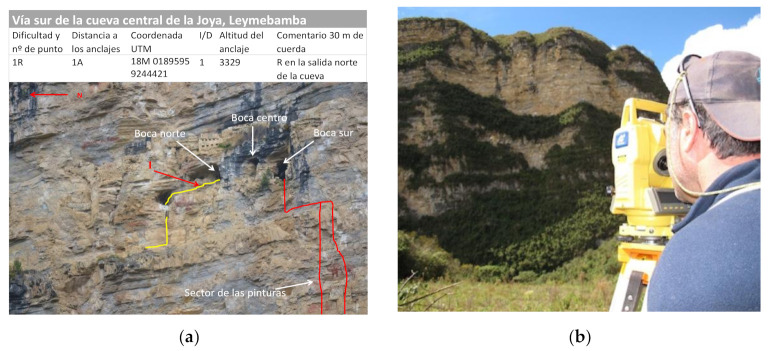
(**a**) An example of a geolocation sheet, and (**b**) geosystems and GPS, Leymebamba.

Data collection and georeferencing by GPS and 3D station of all points of interest are carried out in all the archaeological sites [54]. In the case of rope installations, all anchors and relevant data referring to the difficulty of rope installations are referenced. A particular nomenclature (in Spanish) is adopted to be able to identify these points of interest: P (well or vertical), R (slope), PM (handrail) and A (anchor). Figure 10 shows an example of a geolocation sheet for anchors and points of interest. It is essential to document and reference the installations and anchors for future studies in such a way that they allow a new access without greater visual impact to the site.

### 3.2. Personal Protective Equipment for Vertical Archaeology

#### 3.2.1. Installation

**Figure 11:**
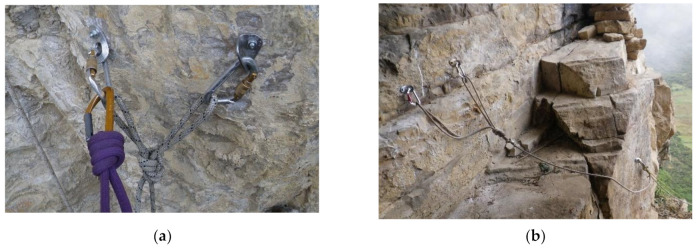
(**a**) Anchor system rigging with a double figure of 8 on the bight (called also bunny knot or bunny ears: (**b**) in-line anchor system, right anchor supports all the load and left anchor only goes into the load in the case of right anchor failure.

Installation is the set of anchors, anchor systems (line-tied), re-belay, knots, ropes and other elements and equipment that are necessary for safe rope progression. The anchor system (line tied) is the upper part of the installation where the loads are distributed to different points of the rocks (Figure 11). In the last phase of expedition, the areas of archaeological interest are accessed with the rope access system regulated in the world of labor. This new proposed method is the clearly differentiating element compared to purely sport techniques or used in the first phase of exploration.

**Figure 12:**
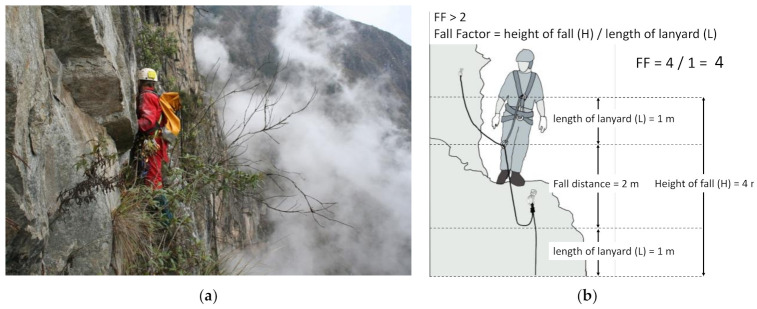
(**a**) Situation of FF > 2 in Qhapac Ñan exploration in the vicinity of Machupicchu, and (**b**) example of FF > 2.

On the other hand, due to the environment where archaeological works take place, it is common that personnel could face more complex situations than in the labor environment. As can be observed in Figure 12a the degree of danger in a fall can be measured by using the Fall Factor (FF), which depends on the user’s position regarding the anchor (Figure 12b). In situations like the one explained above, the FF can be greater than or equal to 2 (maximum FF allowed in the workplace), the definition of FF according to the American National Standards Institute (ANSI) [55]. This index represents a serious fall, so extreme caution is essential when selecting the right technique, materials and PPE.

The PPE resulting from the research carried out and recommended is described in the Table 1 of Section 2 (Materials and Methods). They were divided into two wide groups: generic equipment and specific equipment for the RAS. The logistics planned in such a table do not include the necessary estimation of other aspects such as food, since it depends on the specific characteristics of each project.

Vertical Archaeology, as an adaptation of RAS, is not for experienced users (climbers or speleologists) but for archaeologists, anthropologists, biologists or historians—that is, users with basic training. The proposed techniques will be learned in the training phase and put into practice in the expedition phase.

#### 3.2.2. Knots

Knots have been dealt with in depth by other authors [27,48,56,57], and there is a large bibliography regarding the characteristics of the different knots. In the Ukhupacha project, different types of knots have been put into use, finally making a choice of knots according to the profiles of the researchers. The main safety criteria are established: a low or high loss of resistance R_o_ (residual resistance), simplicity of execution, ease of undoing after being subjected to loads and, finally, simplicity when reviewing. The most efficient and safe knots for the execution of rope progression installations are listed in Table 4.

#### 3.2.3. Ropes

**Figure 13:**
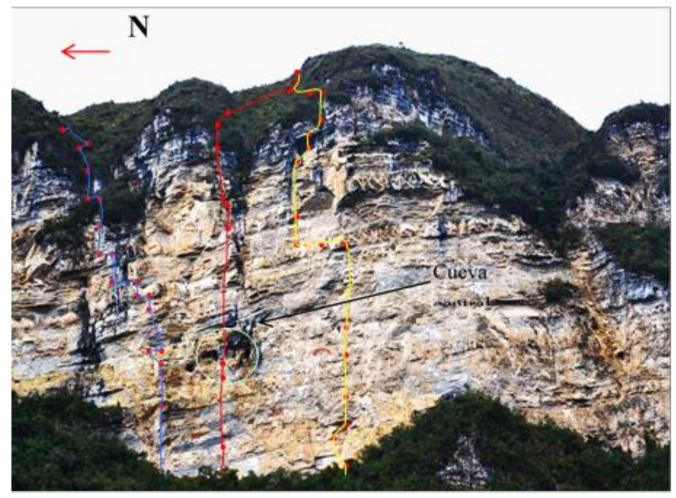
Anchors geolocation at the Joya archaeological site, Leymebamba, Amazonas Department, Peru.

It has been proven that semi-static ropes, with exceptions, are the ones that should be used. They conform to Type A of the UNE-EN 1891 regulation [40]. During rope access (in the training and expedition phases), two ropes are used: the work rope and the safety rope. Both ropes are used equally, and their functions are sometimes interchanged. Therefore, it is necessary that both ropes meet the same technical specifications, to be able to secure or move, allowing the exchange of devices. The fastening of both ropes must be independent at all times (redundant safety), and the diameter that best adapts is 10.5 mm, since these ropes show features that make them fit within more than acceptable safety parameters, both in ordinary work and in possible rescue actions. The 9.5-mm diameter ropes (type B) should only be used for exploration work and only with experienced users. Obviously, they are used due to their greater lightness that facilitates their carrying to places of difficult access (Figure 13).

Semi-static ropes will always be used to progress over them and to handle loads at heights. They will never be used to arrest a possible fall on their own without the use of an energy absorber, system or safety equipment. In such a way, they should guarantee a force received by the injured person with nonharmful values in case of a fall—that is, an arresting force less than 6 kN [45,58,59,60].

The use of ropes that meet the double certification, semi-static and dynamic [40,41], provides a very interesting versatility. The use of this special type of versatile ropes means a significant advance from a safety viewpoint. They offer unbeatable results, because they can be used both in ordinary work tasks and in rescue operations, thus facilitating the application of the Emergency Plan [27].

Finally, it is essential to record and document the geolocation of rope installation for future research (Figure 13).

#### 3.2.4. Anchor

The anchor devices are part of the safety chain with the greatest visual impact in the archaeological site. An inappropriate choice has serious consequences on the conservation of the archaeological heritage.

The anchorage point (the rock, in this case) is the only point of the entire installation without certification from a manufacturer. Due to the physical environments where the projects will be developed, it is a particularly significant point in the installation on which the utmost attention will have to be paid. Experience and accident analyses [17,27,61,62] indicate that a high percentage of accidents are due to failures in the choice of anchorage point. For the choice of the most appropriate anchors, an extensive bibliography has been reviewed, with Marti Puig, Bouthors and Lorenzo Bañuelos [28,29,46].

During the development of the Ukhupacha Project, it has been found that anchors have been installed indiscriminately in many other research projects. The execution of anchors on elements of heritage interest is not acceptable [7]. The choice and placement must be the responsibility of technical experts in vertical works, experts who will previously carry out a detailed study of the anchors, where to locate them, the appropriate type and placement process. The study of the correct positioning of the anchors on the rock is mostly carried out in the exploration phase.

Among the different types, transportable anchors and ultimate-performance screw anchor ones are the most recommended (see Table 5). When removed once the work is finished, they generate less of a visual impact. Furthermore, the case of transportable type B anchors, certified according to the EN 795 [43], present a great safety advantage since, during the process of obtaining the CE marking as category II PPE, they undergo strict manufacturing and production controls [63].

Thanks to them, the user does not have the need to test «in situ» the strength of the anchor during the placement; hence, the supervision of a competent person is enough.

Other safety advantages in the use of transportable anchors are durability (they are removed after use), conservation, traceability and control of the anchor. They are not affected by meteorological factors, oxidation and other problems of continued exposure to the environment, misuse or uncertainty about their use.

However, as aforementioned, the most outstanding feature of this type of anchor is its cleanliness. Once the anchor has been removed, only the hole in the support remains, which can be covered in different ways. Figure (b) in Table 5 shows a makeshift anchorage used by the “huaqueros” (looters) at the La Joya site (Leymebamba).

#### 3.2.5. Rope Adjustment Devices

The rope adjustment devices to be used come from the labor sphere. They are those indicated in the EN 12841 regulation [36]. They are assembled on rope installations of the appropriate type and diameter, in accordance with manufacturer’s specifications. They will allow the user to progress up the rope, vary his or her position along the rope and ascend and descend safely on the rope. They also allow you to securely lock onto the rope. The ascent and descent rope adjustment device (RAC) are installed on the work rope, and the fall arrest RAC is installed on the safety rope [36,64]. The guided-type fall arrester, a safety rope, is essential to guarantee the integrity of researchers, as well as safety against possible errors due to a lack of training and experience in using these systems. The guided-type fall arrester is the characteristic element of a system where scientists are not experts in these techniques. This double rope with fall arrest used in the final phase of the expedition (researchers with little training) is the main difference with the initial phase of exploration, where the expert technical team uses the technique of only one rope coming from the world of sports.

#### 3.2.6. Lanyard Anchor (Cow’s Tail Lanyard) and Energy Absorbers

Within the lanyard equipment certified according to EN 354 regulation [37], those manufactured with dynamic rope must be used, since they are more elastic and have greater energy absorption capacity.

**Figure 14:**
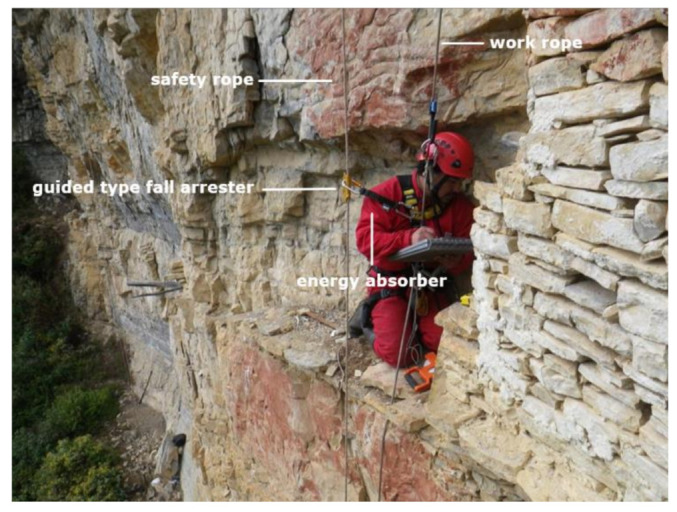
Archaeologist using a double rope. The line on the left is an energy absorber. La Joya site, Leymebamba, Amazonas Department, Peru.

Energy absorbers according to EN 355 [38], as shown in Figure 14, are installed in the safety rope, together with the guided-type fall arrester. The recent appearance of energy absorbers has significantly increased safety when working at heights [19,59,65]. Their main advantage is to ensure a nonharmful arresting force for the user in event of a fall, with a factor of 2 (FF = 2), where the length of the equipment is 2 m, the fall is 4 m and 100 kg is weight. Energy absorbers are selected according to the European regulation as it is more restrictive on safety issues. For instance, they limit the shock forces generated in falls to values lower than 6 kN compared to the 8 kN allowed in the North American regulations [37,38,64].

Other techniques of energy absorption coming from sports, such as securing by using dynamic rope, if well-executed and used by well-trained users, also conform an appropriate technique, but it is only applicable in exploration phase with trained and experienced users. Nonetheless, the main advantage of the dynamic rope, compared to the absorber, consists of not being able to guarantee those impact forces lower than 6 kN under any circumstance. In addition, they require previous experience and constant attention from the securer. Since different profiles and not just climbers work in Vertical Archaeology, this technique is not recommended in the training and expedition phases aforementioned.

#### 3.2.7. Connectors

Connectors are used to link the different elements in the system. They are also the means of a union with anchors; those suitable are those in accordance with EN 362 regulation [42]. Connectors for sports should not be used, since they belong to other areas, have other uses and their technical specifications entail a lower degree of safety requirements than work connectors, as they do not have a safety lock. Connectors used for sports can accidentally open and cause a fall. EN 362 connector closures [42] can be of two sorts: closures with automatic locking and closures with manual locking. The self-closing or automatic connectors are chosen, since they are better adapted to work situations, and they considerably reduce the possibility of accidents due to carelessness (open or incorrectly positioned connectors); hence, safety is increased [42].

#### 3.2.8. Helmets

Protective helmets are PPE-designed to cover the head and contribute to reduced damage derived from impacts caused by dropping objects and small collisions with fixed elements. The current regulations recommend selecting a helmet that is adapted and meets the requirements. As an essential requirement, the need for a chinstrap is highlighted. Therefore, it must be certified in accordance with EN 12492 [39]. It is interesting that the helment simultaneously meets several certifications (sports and work). It must comply with the requirements of the sports standard, regarding the resistance of the chinstrap, and those of the industry regarding the protection of falling objects. It is also crucial that they have a lighting system, since, in many occasions, archaoelogical findings occur in dark places, inside cavities or even at night [39].

#### 3.2.9. Harness

**Figure 15:**
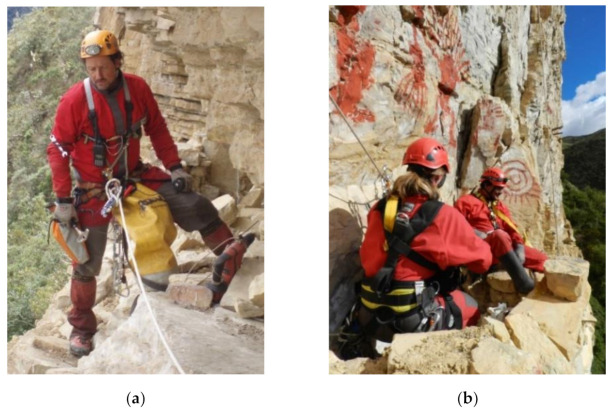
Differentiated PPE for different phases. (**a**) PPE for the exploration phase. (**b**) PPE used in the last stage of an expedition.

In the world of sports, there are many types of harnesses, such as those for speleology, canyoning or climbing, all of them missing suitable technical requirements for arresting a fall with impact (Figure 15). These harnesses are not valid for Vertical Archaeology work, since their design is not conceived to retain a fall safely [66]. In these jobs, where most users lack experience, the best option for the training phase and the expedition phase is an integral-type harness, since it simultaneously has the specifications contemplated in several certifications: EN 358 for restraint harness [34], EN 361 for arrest fall harness [33] and EN 813 for seating [35]. 

For the exploration phase (with specialists), the most commonly used harness is usually a harness such as those used speleology; although, for certain tasks, other specific combinations with appropriate fall arrest anchors may be required. Figure 15a shows an example of equipment in the exploration phase, and Figure 15b presents an example of the equipment used in the training and expedition phases.

### 3.3. Emergency Plan

Precisely because of the peculiarity of the environment and the high response time required by professional emergency services, it is essential to have an Emergency Plan prior to any start of activity that may provide immediate help in the event of an accident [27]. This is possible thanks to the special work of several members of the Ukhupacha team, such as rescuers belonging to different rescue groups and their great experience in the development and implementation of new procedures for firefighters in Spain and Latin America [27]. Moreover, experts have extensive experience in speleology, cave rescuing, climbing, mountaineering and canyoning, as well as other mountain disciplines. All those sports based on ropes and harness management have been the source for many techniques used in Vertical Archaeology.

As an example, the accident that took place in the Department of Amazonas, near Leymebamba in Peru in 2013, in a cave of archaeological interest known as Inti Machay is cited. Two members of the Ukhupacha Project participated as cave rescuers. The complete rescue, from the accident until the last rescuer left the cave, took 20 days, of which the injured person spent 12 days inside the cave until his extraction [61,62,67,68]. The immediate availability of all the necessary materials was really complicated. Fortunately, the intervention time was greatly reduced thanks to the use of PPE equipment, a rescue stretcher and materials from the Ukhupacha Project that were located nearby at an archaeological site in Peru.

**Figure 16:**
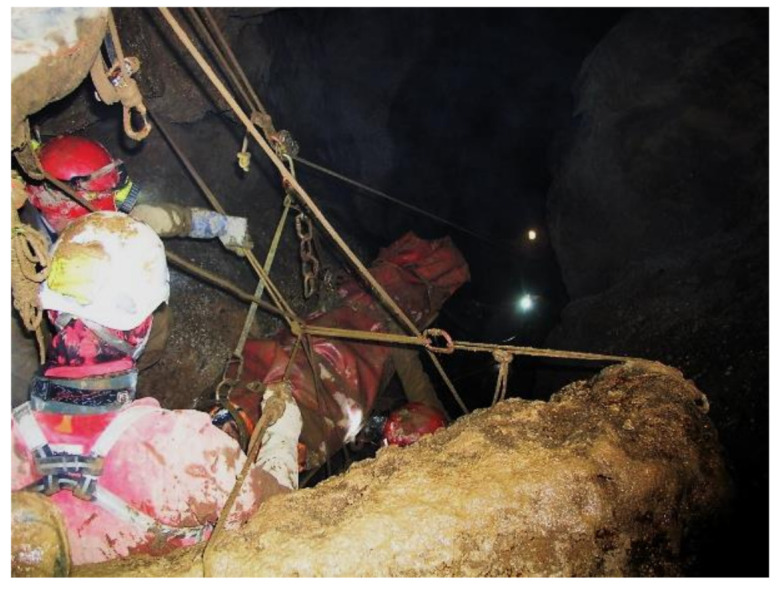
Real rescue of a caver (spelunker), Inti Machay, Amazonas Department (Peru, 2014).

A major risk in the use of RAS is Harness Syndrome [69,70,71] or suspension trauma, a subject widely studied by other authors and treated in numerous medical congresses of mountain health specialists. Given the complexity of the Andean environment, the intervention of professional rescue services cannot be waited on, since most of the action takes place in archaeological sites that are so difficult to access that rescuers may need several days to arrive. Hence, it must be the users of the RAS themselves (Figure 16) who are prepared and carry out the emergency procedure [30].

For all the aforementioned, the Emergency Plan must clearly differentiate between Emergency procedure (self-help) and rescue. Emergency procedure is carried out by the personnel present at the incident (workmate rescue/retrieval) with the means and materials available at that time. The rescue is carried out by specialized professional groups with the specific means, techniques and materials that these bodies have at their disposal.

Examples of rescuing in this environment have been cited, and accidents due to work at a height are mentioned bibliographically. The importance of collecting statistical data regarding accidents in the environment and as a future field of research is highlighted. This would provide a remarkable documentary support to the work reported in this article (More information in Supplementary materials). 

**Figure 17:**
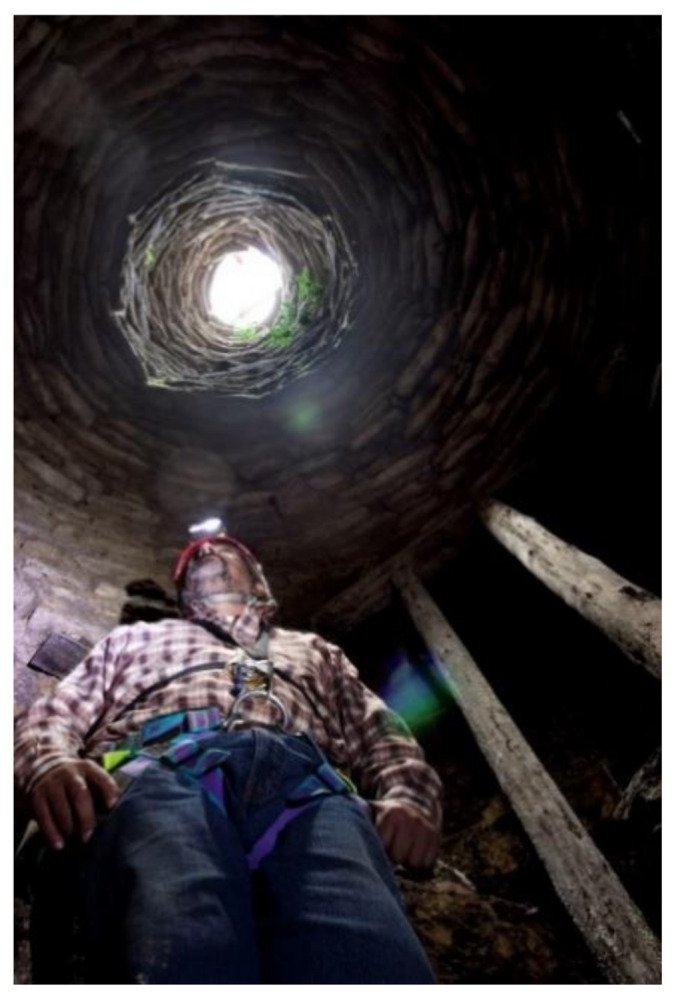
Tintero study: new funeral patters in the city of Kuelap.

Hence, the panorama of future applications of rope access techniques offers an unbeatable solution that can help and facilitates the recovery of the historical identity of all these cultures (Figure 17). The annual trainings, as technical assistance provided to the current Ministry of Culture (formerly INC Peru) through the Ukhupacha Project, helped local teams to autonomously employ techniques for the maintenance, conservation and restoration of inaccessible sectors of the Inca roads. It is expected that the seed started by the Ukhupacha Project and all the experience accumulated during more than two decades of work will provide solutions to improve the safety and can serve as a reference guide for future research projects and conservation of Andean heritage. In the same way, it is hoped that it can help to solve unknowns such as “What kind of techniques and means did these cultures use to execute all this heritage in such vertical and inaccessible places?”

## 4. Conclusions

The proposal for the organization of works in three phases (exploration, training and expedition) is an important novelty compared to previous research projects. In particular, it represents a remarkable advance in safety for researchers, allowing access to places that were previously inaccessible. For the first time, active safety and prevention techniques are incorporated into this type of work, as well as specific PPE for each phase of work and type of intervention. Particularly, the methodological proposal with work procedures (phases or stages), the Emergency Plan and self-help techniques facilitate a notable reduction in risks and a decrease in the number of accidents, which are important increases in researchers’ safety.

The projects carried out have helped to better understand the pre-Hispanic civilization of the Andean environment and has made it easier for researchers to safely access and study all this great heritage. For instance, due to the vertical challenge completed in the study of the Qapac Ñan, there is a greater depth in the scientific knowledge of Tahuantinsuyu or Chachapuyas culture. In short, the proposed methodology has enabled the enhancement of those hidden and inaccessible cultural heritages until now, opening new paths and study possibilities for the analysis and conservation of historical heritage.

Moreover, researchers have been able to perform their work more safely in hitherto unknown and unexplored environments, opening up new avenues of research. Additionally, in the different aforementioned projects, the importance of forming multidisciplinary teams has been proved in order to approach the study of archaeological finds from all aspects of interest: anthropological, artistic, constructive or architectural.

Currently, a process of improvement and updating of all these equipment and techniques is taking place, as the training processes and application of systems must be continuously reviewed. The appearance of new technologies machinery and tools such as surveying, 3D photogrammetric or unmanned aircraft (DRON) allow a safer approach to research sites, shortening deadlines and providing new lines of exploration.

With the received training, researchers have achieved a certain degree of independence in rope progression. Notwithstanding, this independence does not qualify for an expedition without having a multidisciplinary team of experts who provide advice on safety matters, as well as the experience of specialists in vertical techniques and rescue. As shown, the occurrence of an accident can have serious consequences if an adequate risk assessment is not carried out and a specific Emergency Plan is not designed. The application of rope access techniques requires a level of specialization that implies continuous training for researchers. To put it briefly, it is not possible to get involved in these projects without prior training that guarantees the safety of the users.

The good reception of this new working method among the scientific community, researchers and organizations related to heritage conservation, means that collaboration projects with ministries, universities, administrations and organizations related to heritage conservation proliferate year after year for the realization of new expeditions.

The numerous projects carried out successfully and without accident by the Ukhupacha Project over the last few decades [72,73,74,75,76,77,78,79,80,81,82,83,84,85,86,87,88,89,90,91,92,93,94,95,96,97,98,99,100,101,102,103,104,105,106,107,108,109,110,111,112,113,114,115,116,117,118,119,120,121,122,123,124,125,126,127,128,129] prove the suitability of the established method. The techniques, personal protective equipment and systems used are adequate, safe and reliable. In addition, they provide a novel way of approaching scientific exploration (More information in Supplementary materials).

Hence, it is expected that the scientific methodology used will evolve in a multidisciplinary way, where archaeologists, anthropologists, biologists, historians, architects and engineers can contribute. In this way, it will be fully developed in all areas: exploration, academic works, training and research.

## Figures and Tables

**Table 1:** Personal protective equipment (PPE), selected materials and equipment for use in Vertical Archeology.

Generic Equipment and Individual Use of PPE	Specific Equipment and Collective Use of PPE	Logistic, Other Equipment and Materials
Full harness (EN 361 (arrest a free fall) [33] EN 358 (work positioning harnesses) [34], EN 813 (sit harnesses) [35]) Rope adjustment devices EN 12841 [36]: A Type (Back-up devices) B Type (ascending devices) C Type (Descending devices) Lanyard, EN 354 [37] Energy absorber, EN 355 [38]. Helmet EN 12492, with chinstrap and lighting system [39]. Snap Knife (workmate rescue/retrieval) Gloves, overalls Technical clothing: rain, humidity, heat, coal and height Suitable footwear; rain boots, mountain shoes. Leggings. Lantern	Ropes: 9.5 mm, low stretch kernmantle rope type A EN 1891, (exploration) [40] 10.5 mm, dynamic rope EN 892 (exploration) [41] 10.5 mm, low stretch kernmantle rope type A EN 1891, (expedition) [40] Different classes of Connectors EN 362 [42] B Type: symmetrical and asymmetrical regular use M Class: multipurpose, forces according to two load axes Q Class: thread for permanent use Anchors EN 795 [43] Undercut EN 795-A (exploration phase) Transportable Anchor, EN 795-B (exploration and expedition) Anchor sling EN 795-B, (exploration and expedition)	Folding rescue stretcher Drill: batteries and charger High performance solar panels Drill bits of different diameters GPS Total station Portable radio Phones Photo camera Binoculars Computers Blueprint protector Sleeping bag Mats Tents Solar showers First aid kit Thermal blankets Flexometer Laser meter Stationery: notepads, folders, pencils, markers
	Pulleys, EN 12278 [44] Bag for installation material and anchors: hammer, burin, spanner and caving nail. Anchor plates: Bent Twisted clown guy You take out various sizes	

**Table 2:** Differences between the working world, sports and vertical archeology.

Subject	Working World	Sport World	Vertical Archaeology
Safety	Mandatory	Volunteer	Mandatory
Technical	Work rope + safety rope	One rope	Exploration one rope, expedition and training two ropes
	Fall arrest harness	Pelvic sport harness	Specific harness
	Worker health and safety criteria predominate	Criteria of lightness and comfort prevail	First and foremost, safety
workmate rescue/retrieval	Simple techniques	Complex techniques	Greater effectiveness from suspension trauma (also known as suspension trauma, suspension syncope)
Training	Mandatory	Volunteer	Mandatory
Users	Little specialized, based on PPE	High degree of specialization	Inexperienced researchers

**Table 3:** Ukhupacha Project results from 2002 to 2017.

Year	Expedition	Duration (Days)	Number of Rope Access Technicians	Hours Worked (No Technicians × No Days × 8 h)
2002	Chinkanas, Machupicchu site (a collaboration with INTERFASI project)	30	8	1920
2003	QHAPAQ ÑAN expedition and cave catalog	25	9	1800
2004	Machupicchu, San Miguel path	20	4	640
2005	Inca bridge path expedition. SALAPUNKU cave/rock painting	20	13	2080
2006	QHAPAQ ÑAN. Culture Ministry	25	15	3000
2007	Continuation of the Machupicchu Expedition, Kuelap	25	12	2400
2008	Cusco and Amazonas exploration	20	10	1600
2009	New Inca path exploration, Cerro, Machupicchu	35	8	2240
2010	Topographic of Puente Inca, Machupicchu	30	8	1920
2011	Cave de Shigual exploration, Magdalena. Cave/Rock paintings, Inkapintay (Ollantaytambo)	25	12	2400
2012	La Joya and La Petaca exploration, Leymebamba.	20	11	1760
2013	Leymebamba project continuation.	15	15	1800
2014	INTI MACHAY rescue	15	2	240
2015	Scientifics Training, Culture Ministry, Cusco and Hot Waters (Aguas Calientes)	20	2	320
2016	Scientifics Training, Culture Ministry, Cusco and Hot Waters (Aguas Calientes)	20	2	320
2017	Tetuan Dungeons Project collaboration, University of Alicante	15	4	480
TOTAL		340	133	
Total Hours worked				24,920 h
TOTAL HOURS IN SUSPENSION				12,460 h

**Table 4:** Results: applicable knots.

Use	Type of Knots					
	Figure of Nine on the bight	Double Fisherman’s	Overhand Knot		Bowling Knot	Figure of Eight on the bight
Loading Knots	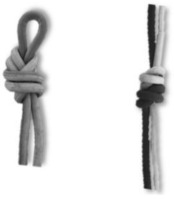 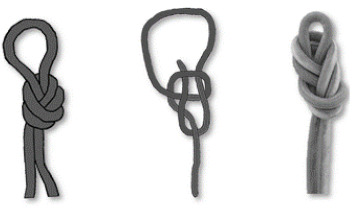					
Join Knots	Double Fisherman’s 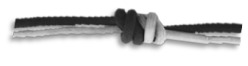			Water Knot 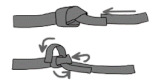 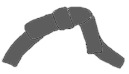		
Locking pulley	Machard 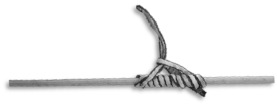					
Hitches	Clove 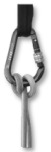			Munter 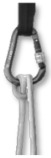		

**Table 5:** (a) Anchor types recommended and (b) looters anchor (boat anchor type).

Type A Anchor EN 795, Undercut Anchor [43]	Type B Anchor EN 795, Transportables [43]	Makeshift Anchor by Looters
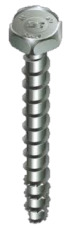	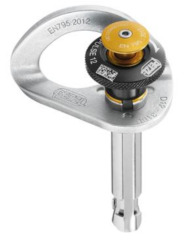	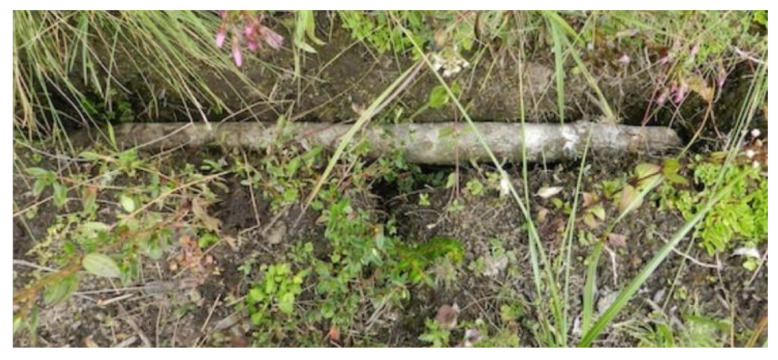
noninvasive anchors, they are easily removed, leaving no remains of the installation, they do not generate serious damage to the heritage		boat anchor type used by looters
(**a**)		(**b**)

## Supplementary Materials

The following are available online at https://www.mdpi.com/article/10.3390/ijerph18073536/s1: Video S1: Presentation of the Ukhupacha Project and Emergency Plan Rescue in Inti Machay.
