# Supplementary material for: Vertical Archaeology: Safety in the Use of Ropes for Scientific Research of Pre-Columbian Andean Cultures
Source: Int J Environ Res Public Health. 2021 Mar 29;18(7):3536. doi: 10.3390/ijerph18073536 (PMC8036328; doi:10.3390/ijerph18073536)

## Slide 1
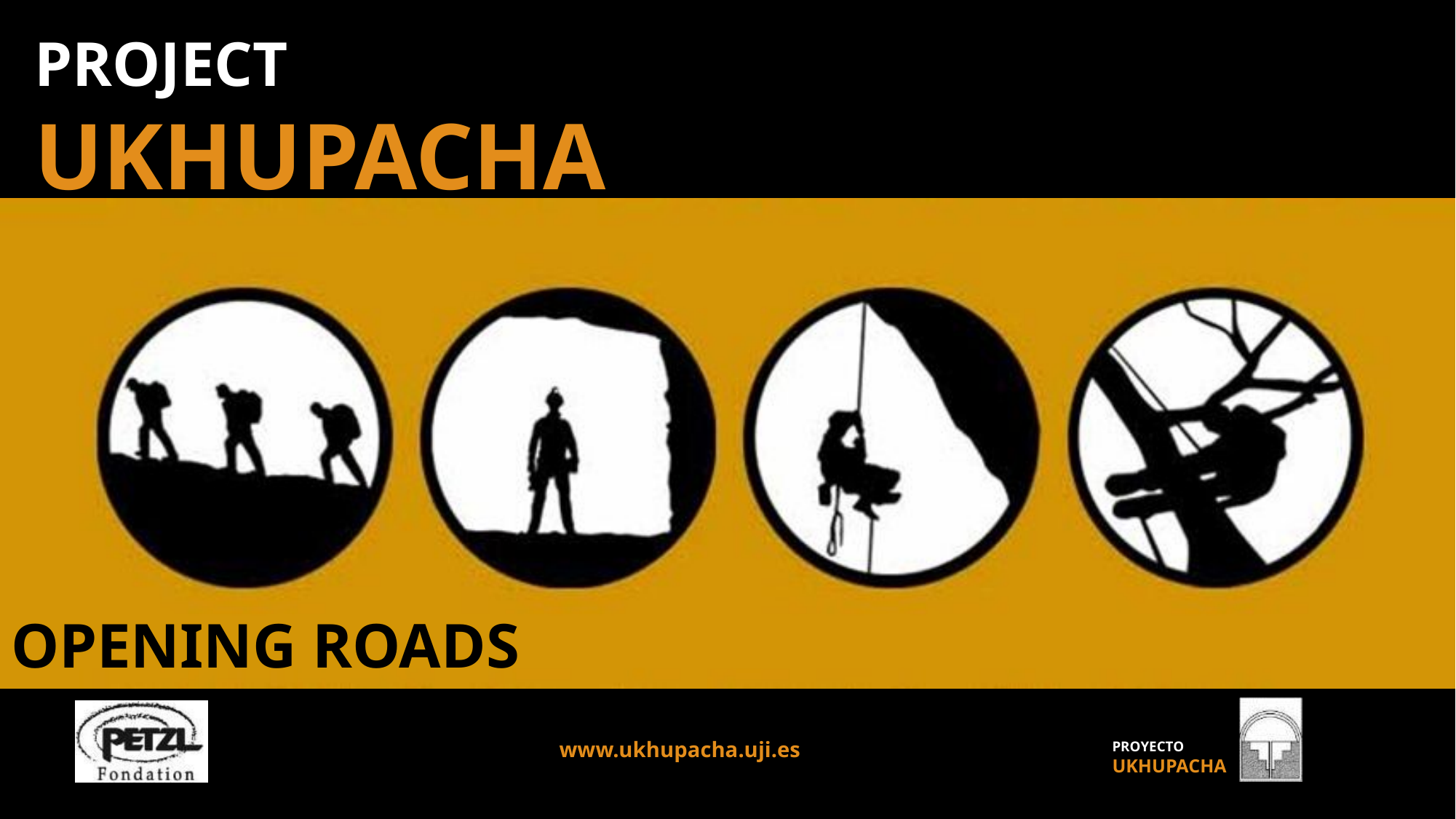

## Slide 2
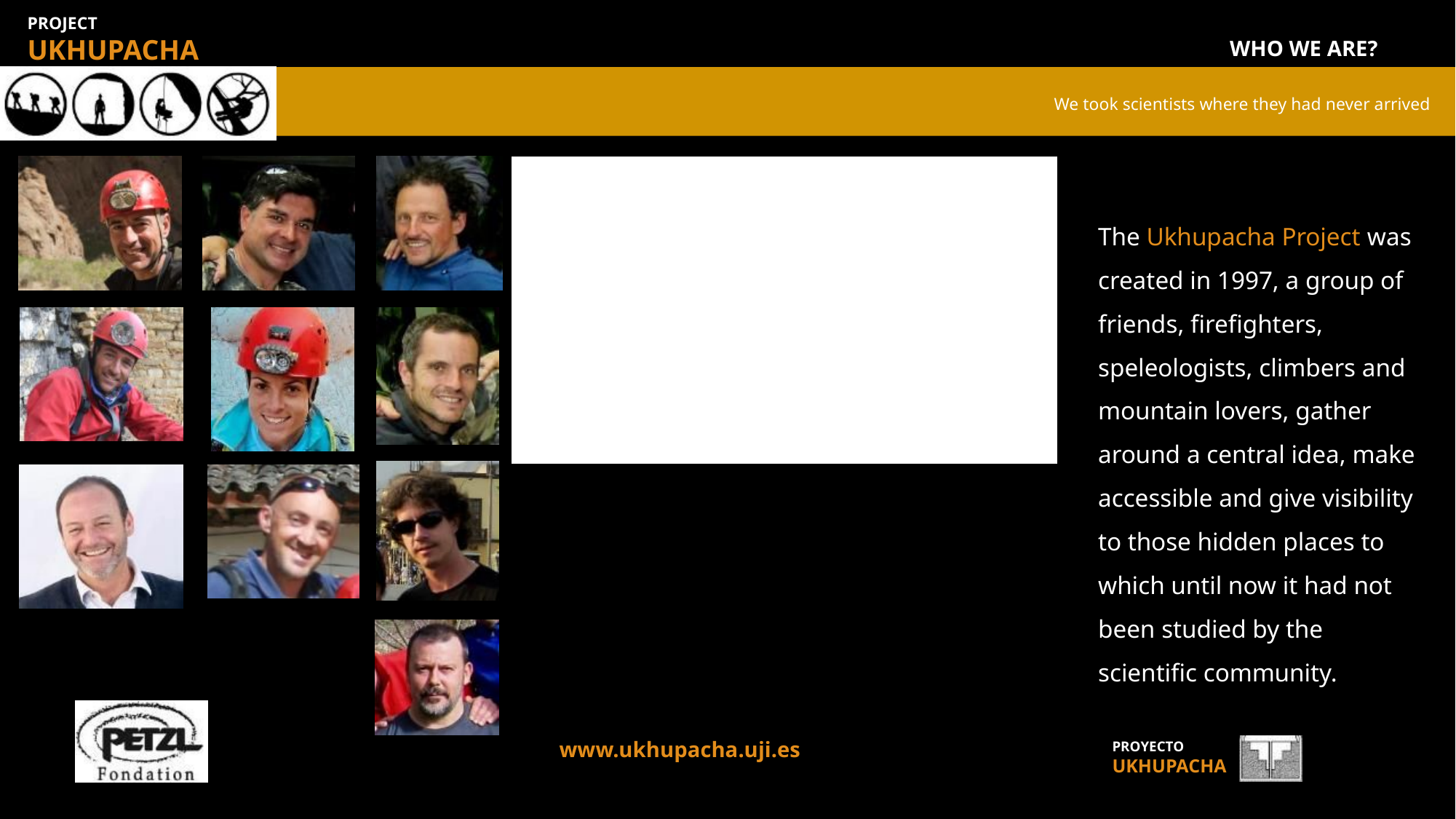

## Slide 3
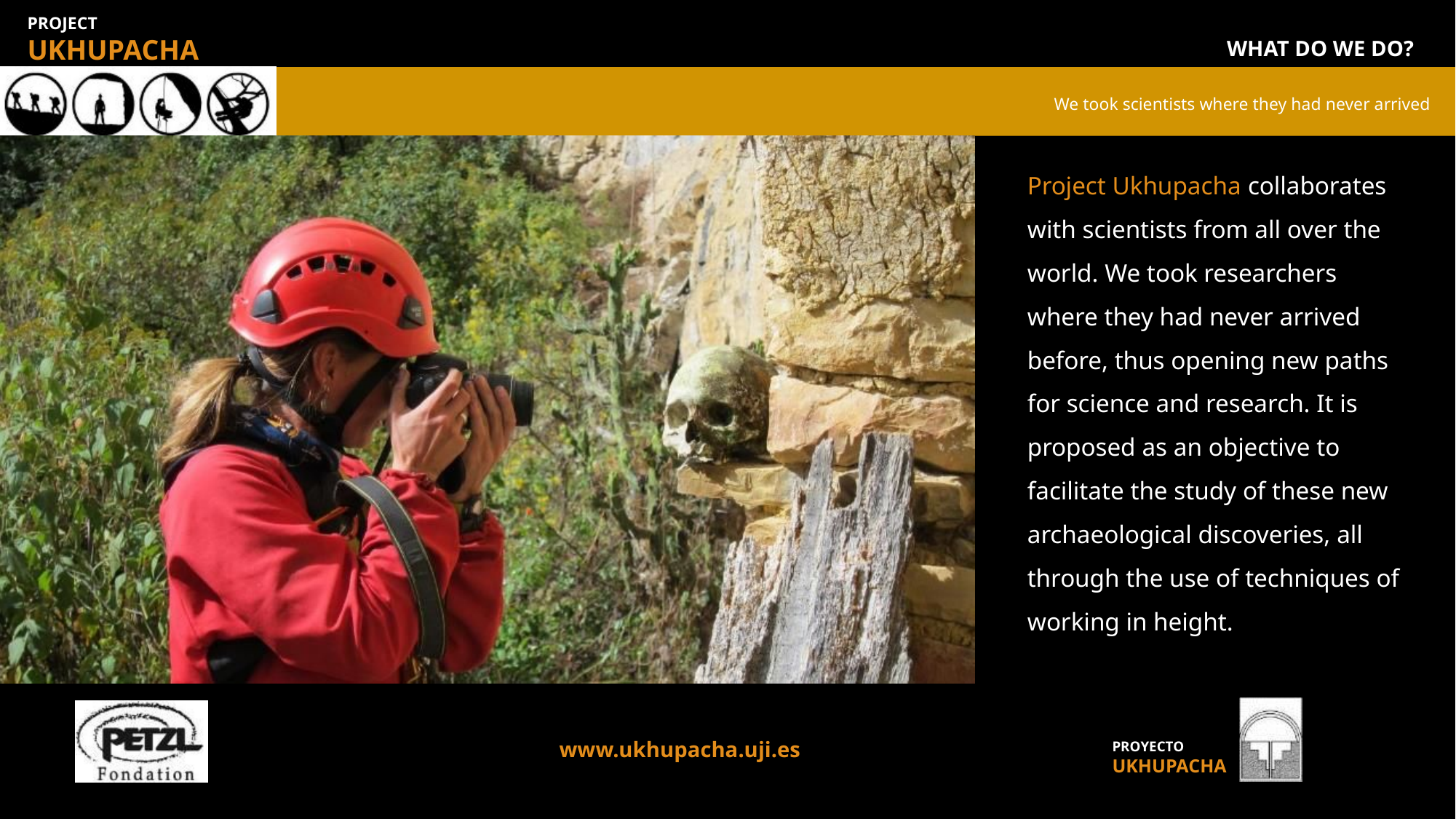

## Slide 4
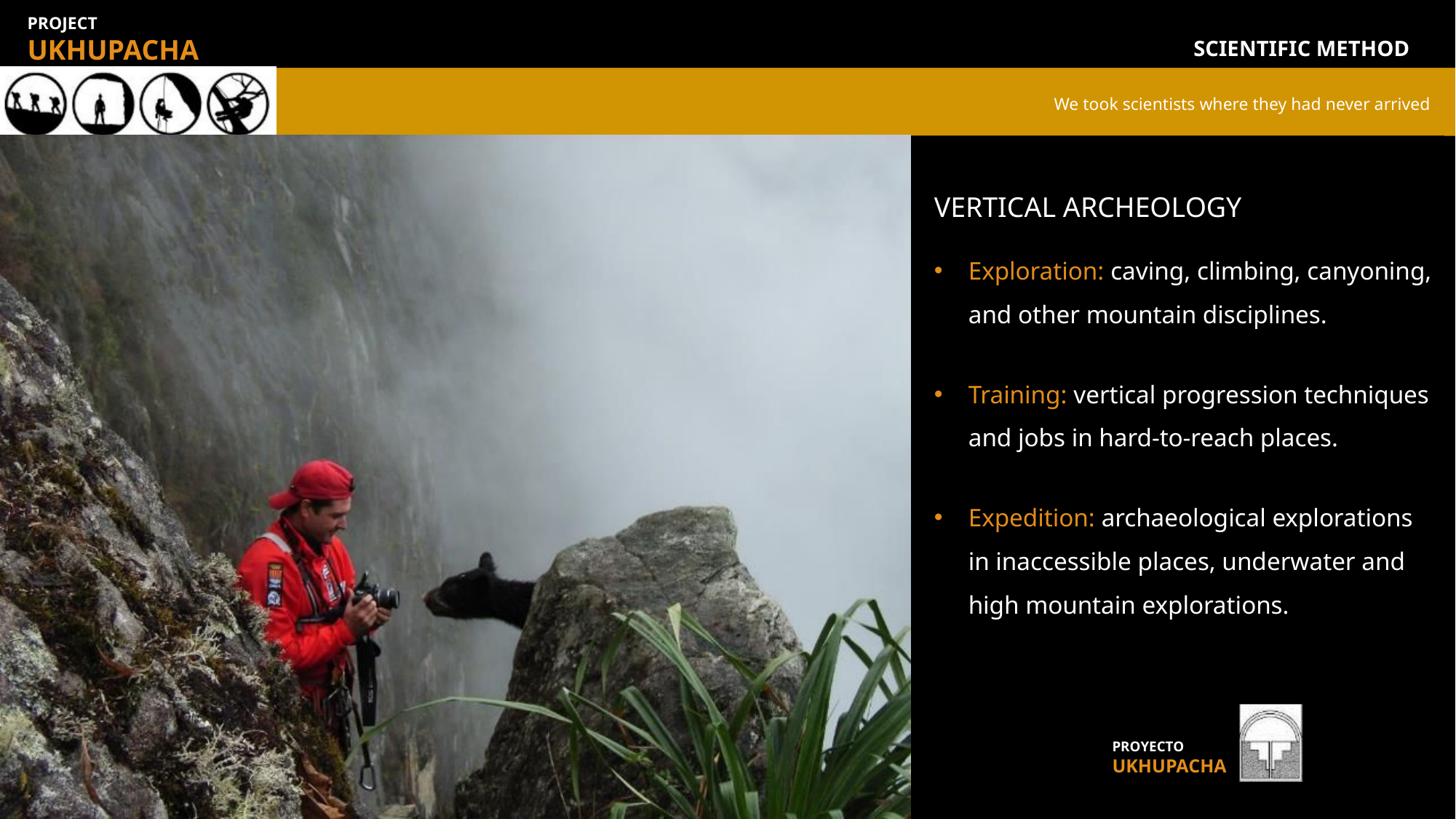

## Slide 5
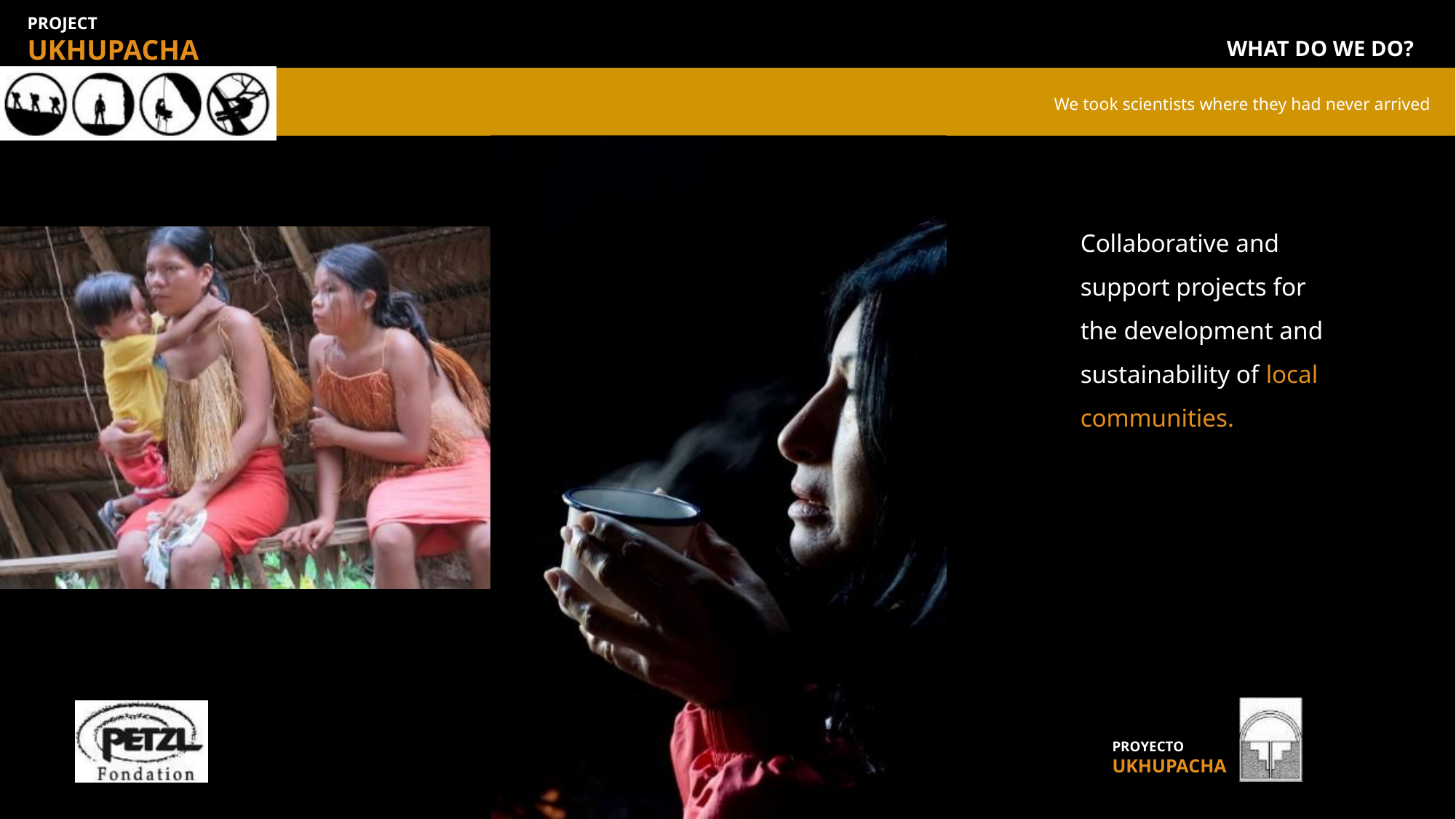

## Slide 6
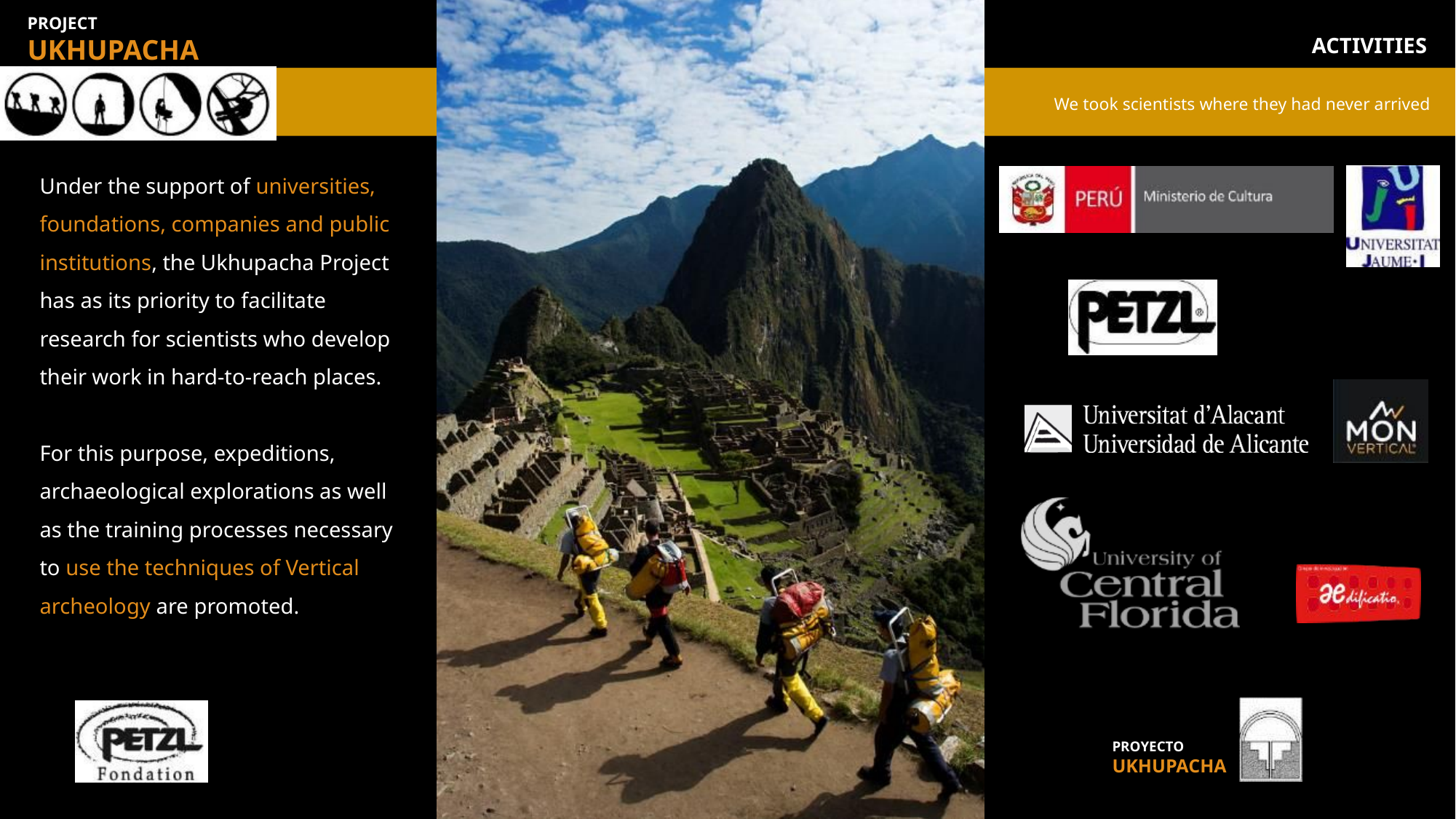

## Slide 7
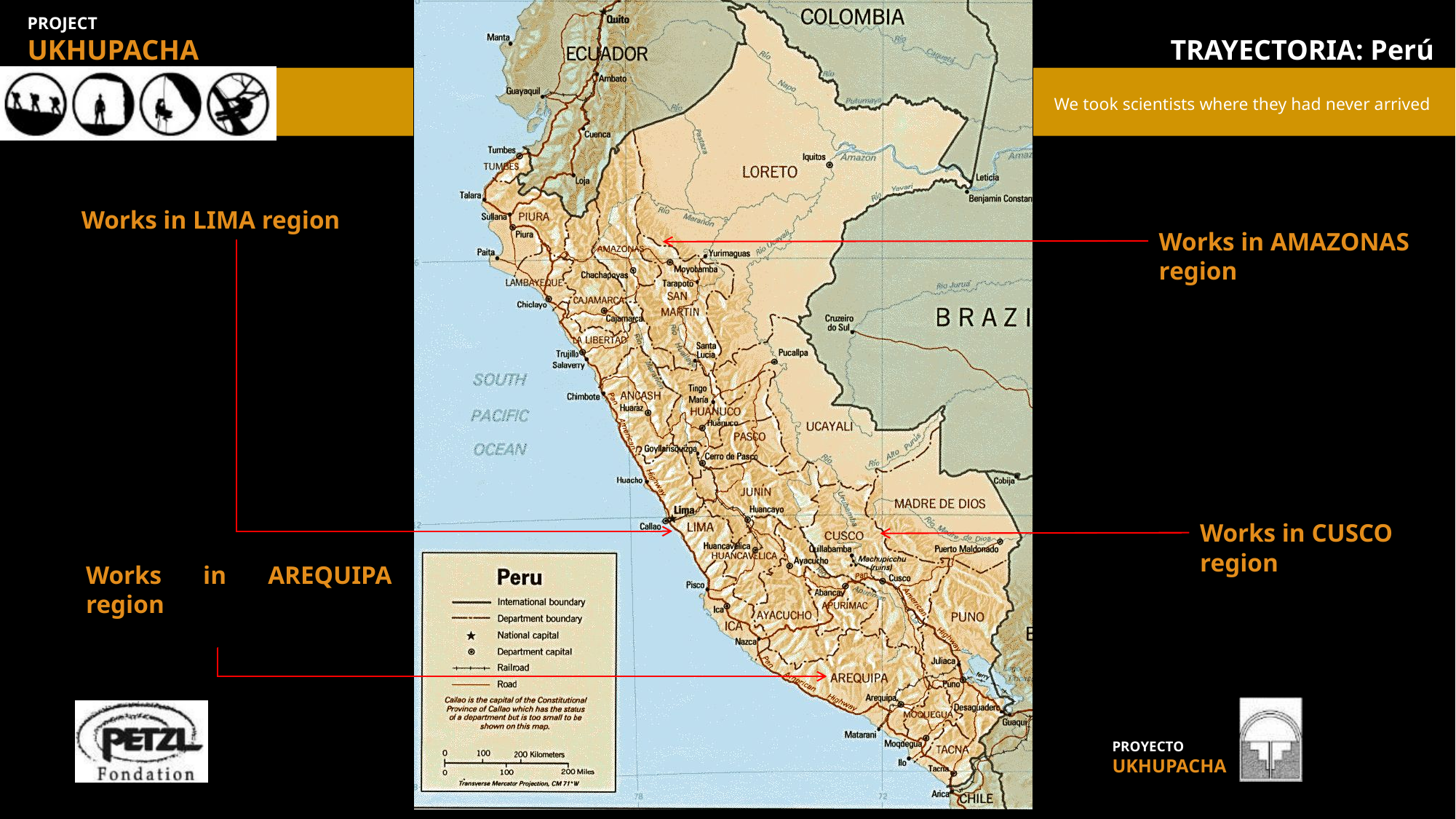

## Slide 8
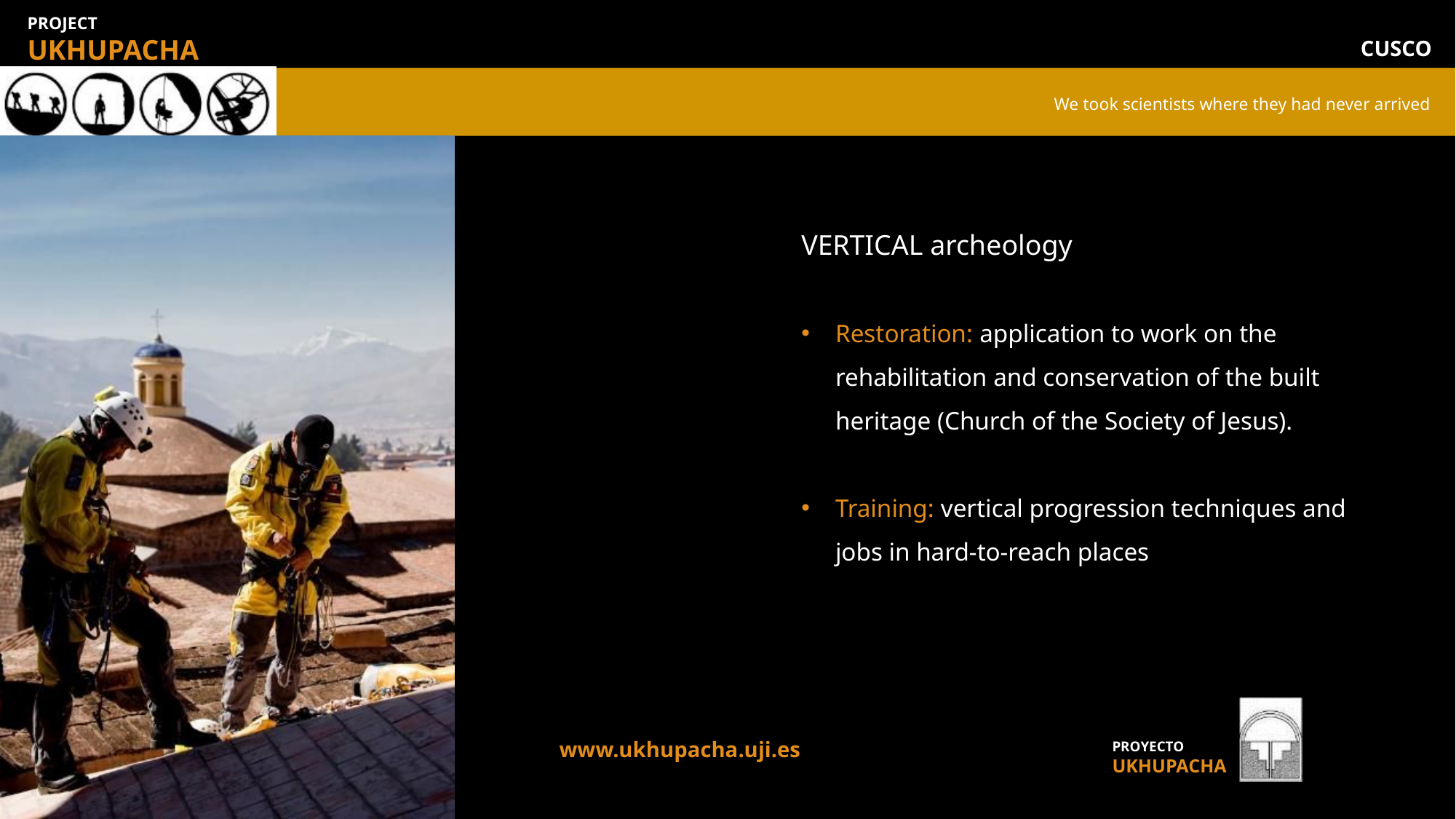

## Slide 9
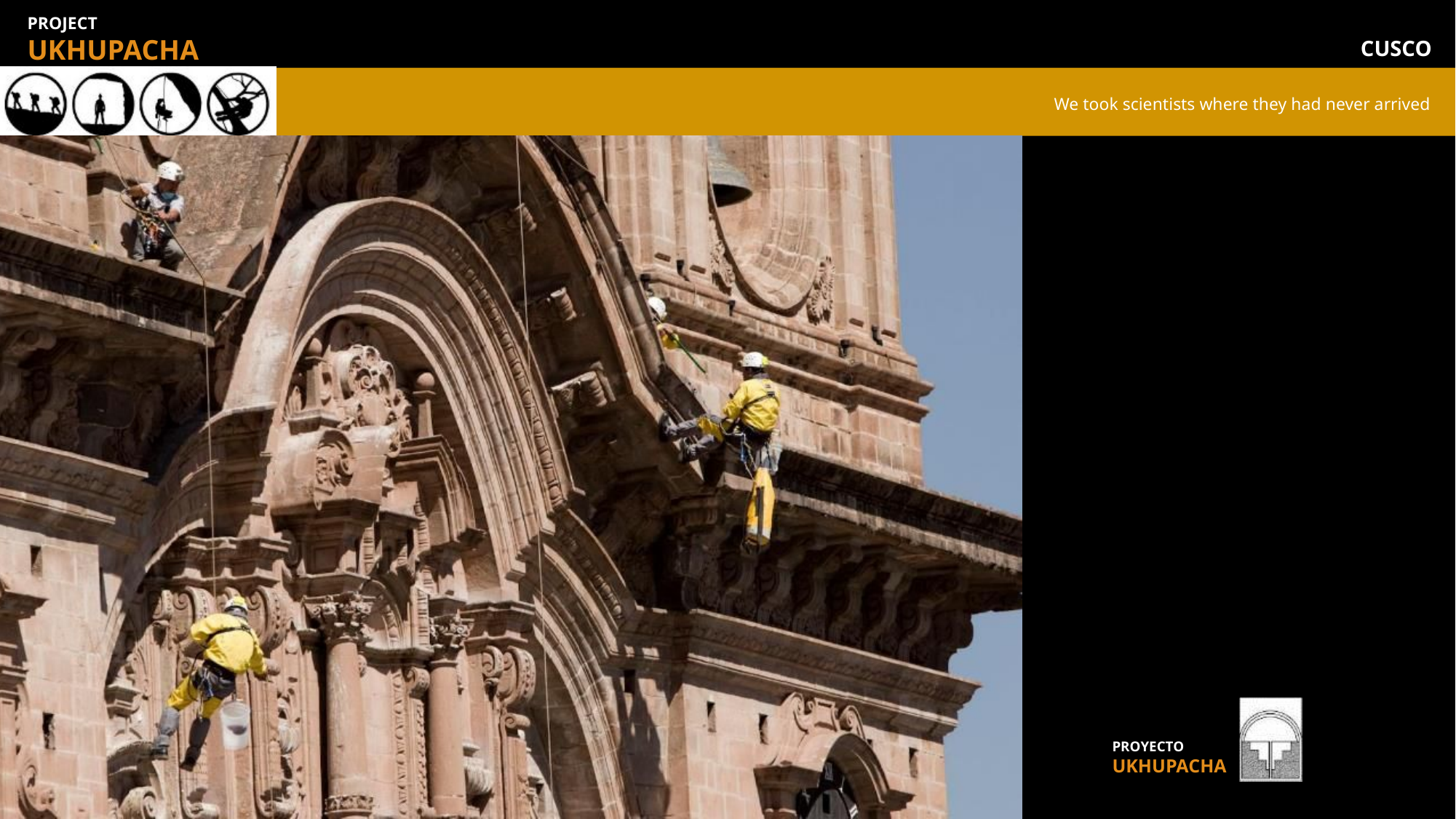

## Slide 10
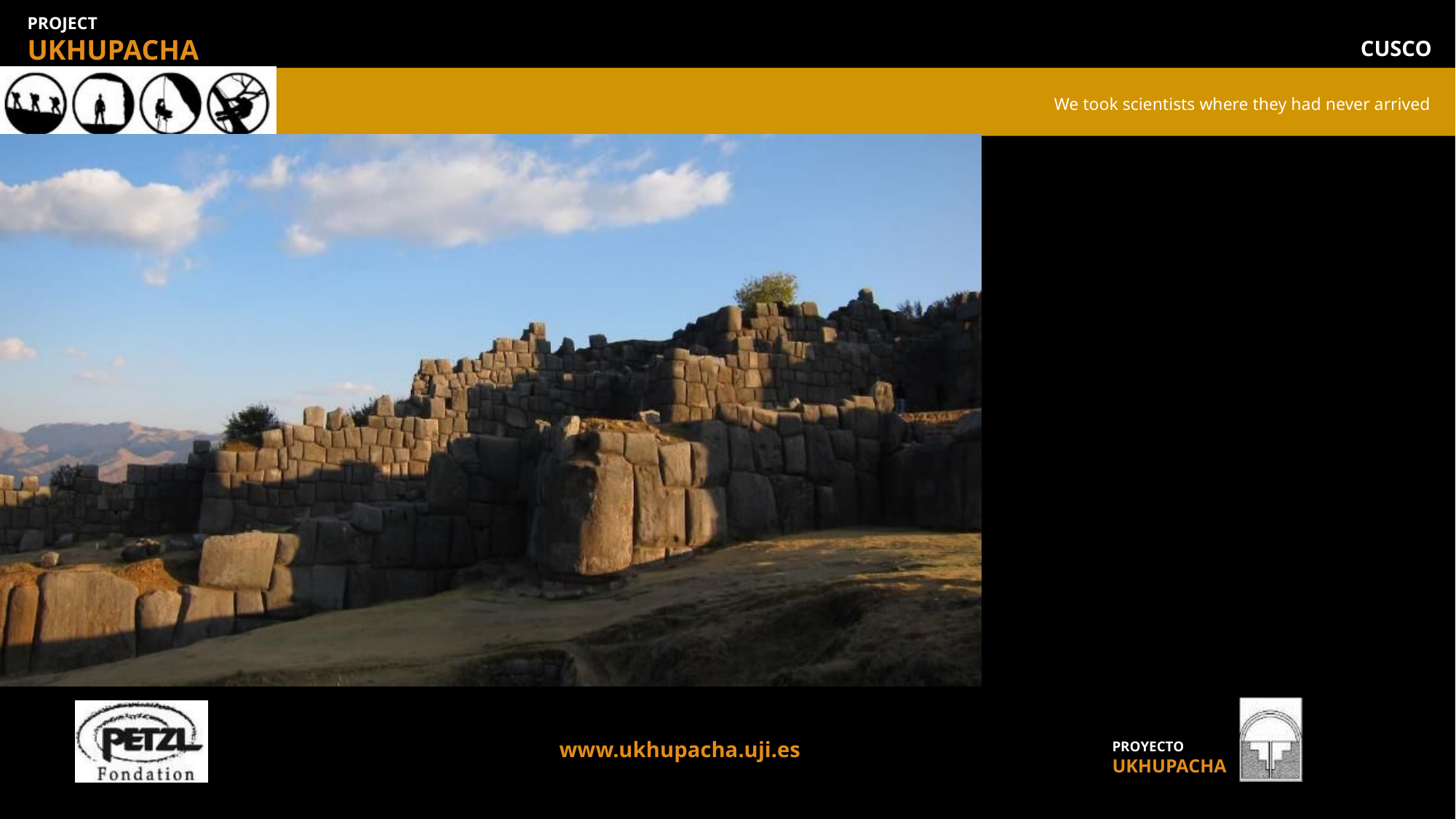

## Slide 11
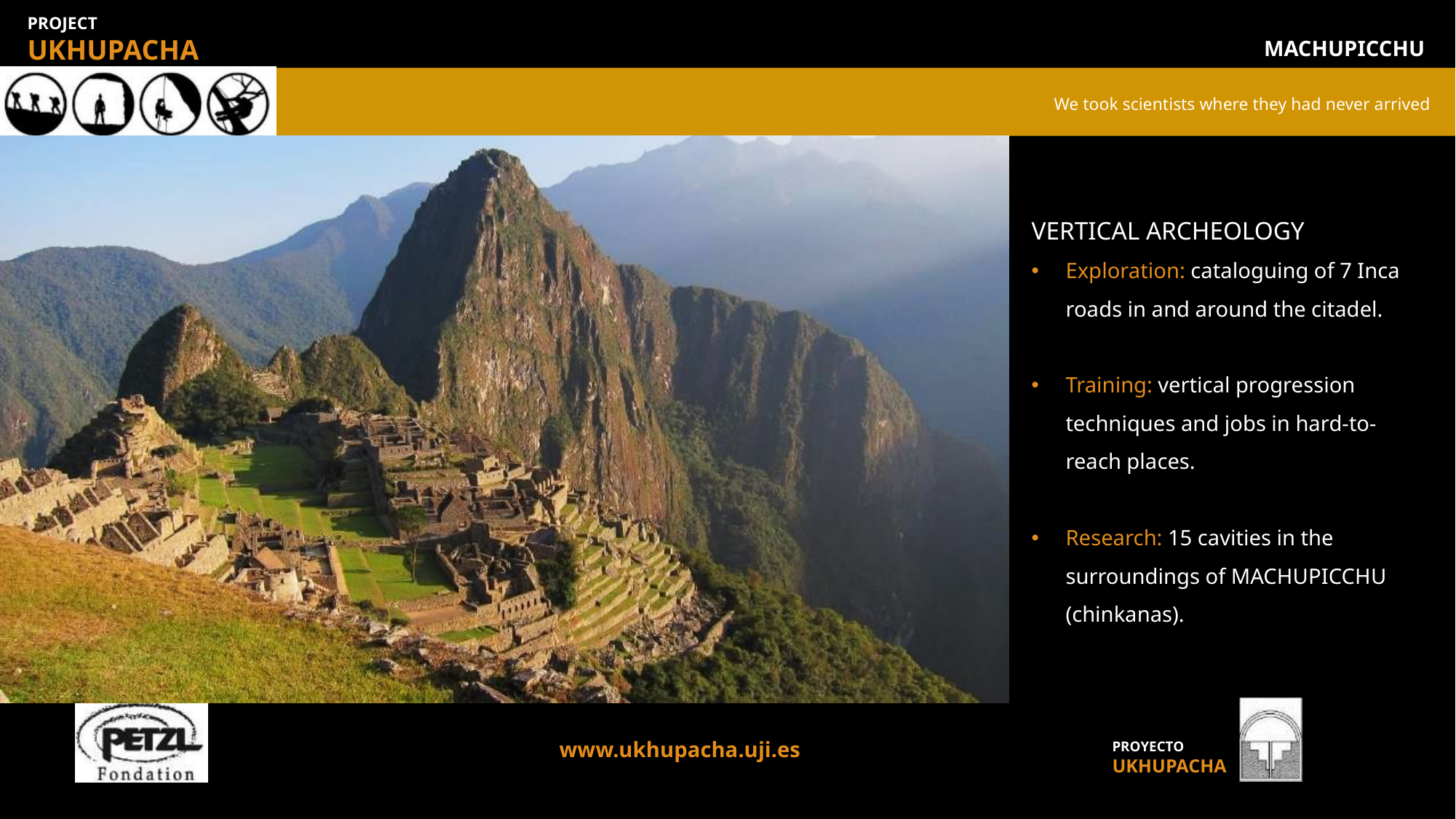

## Slide 12
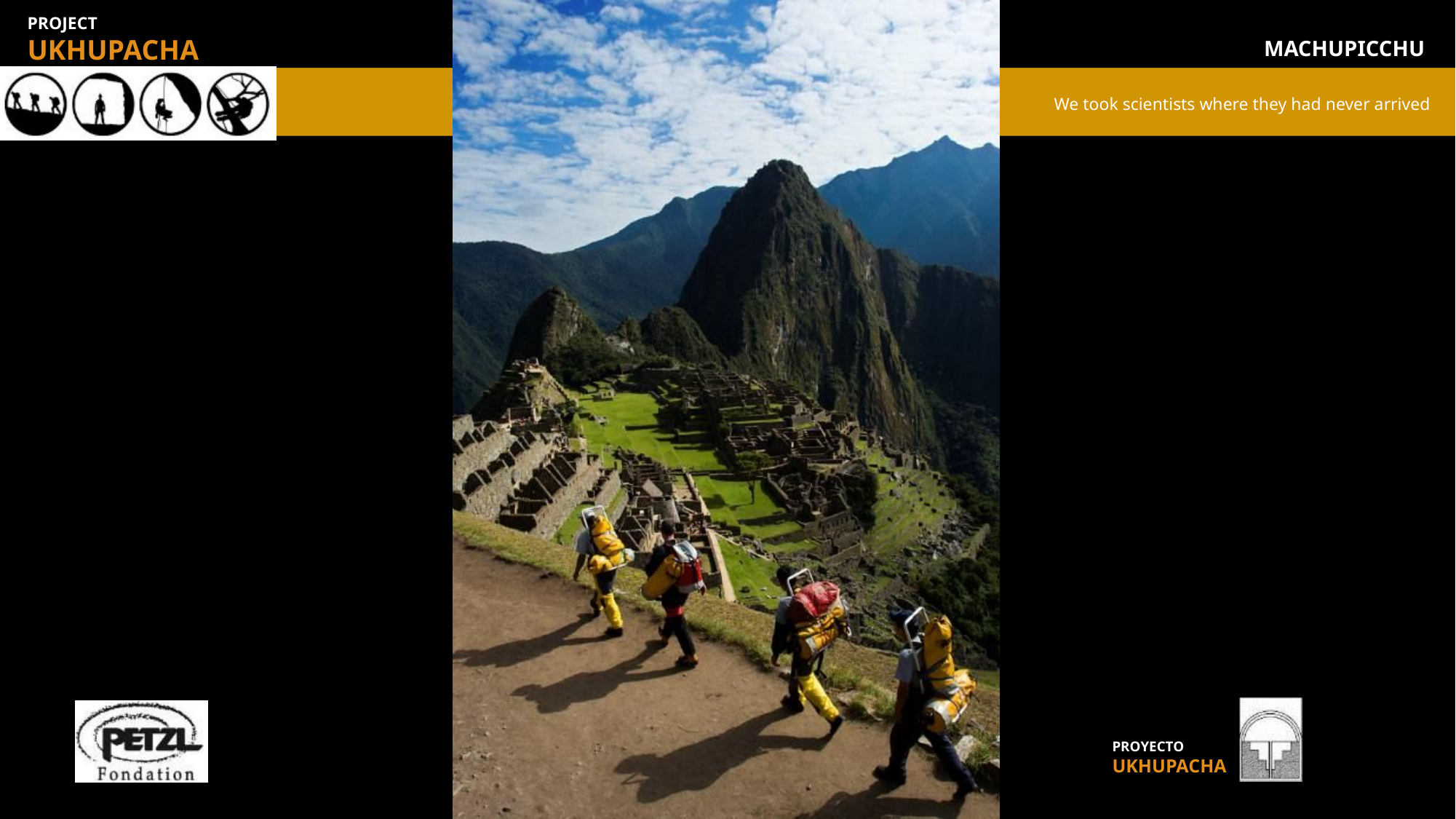

## Slide 13
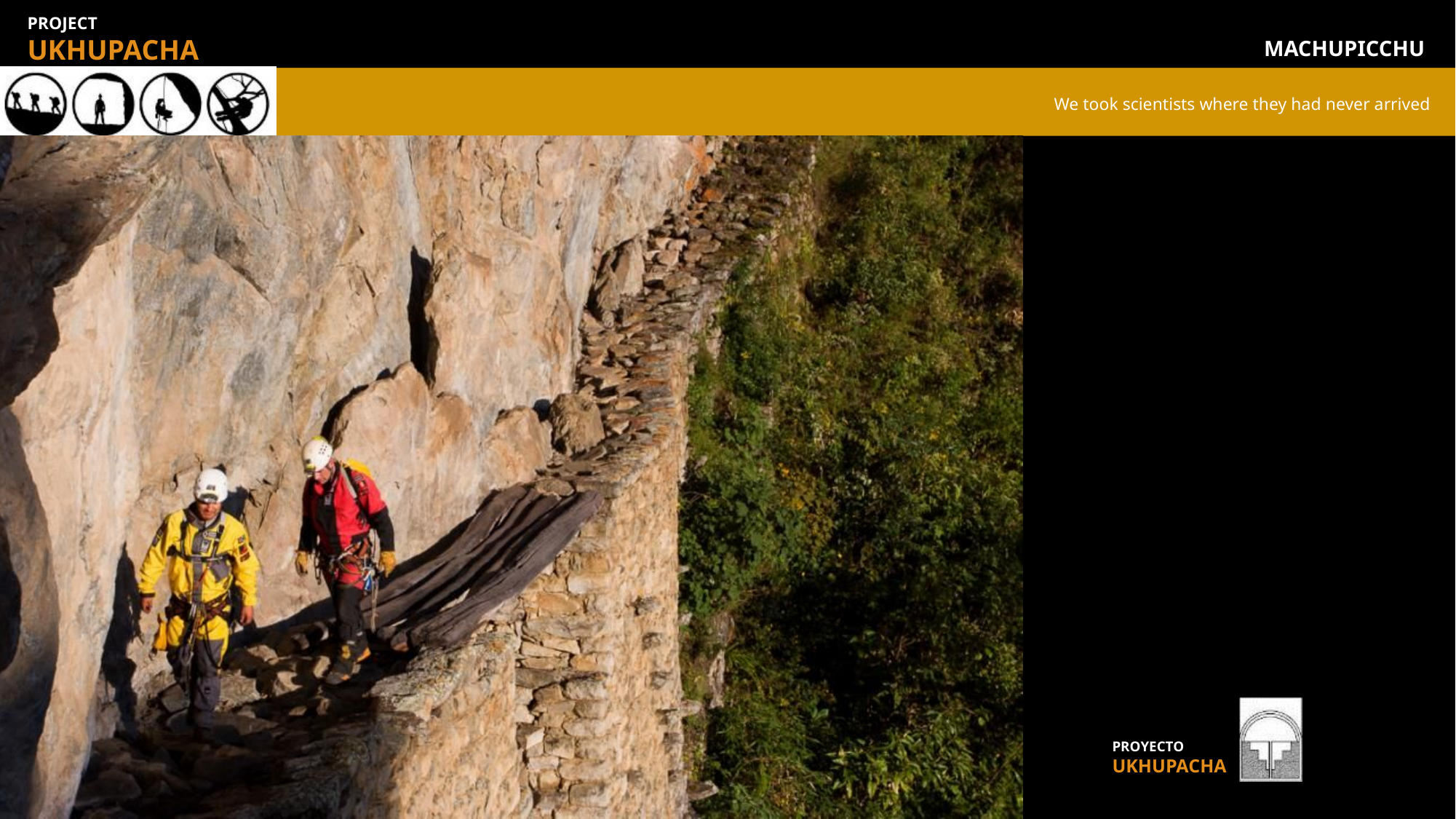

## Slide 14
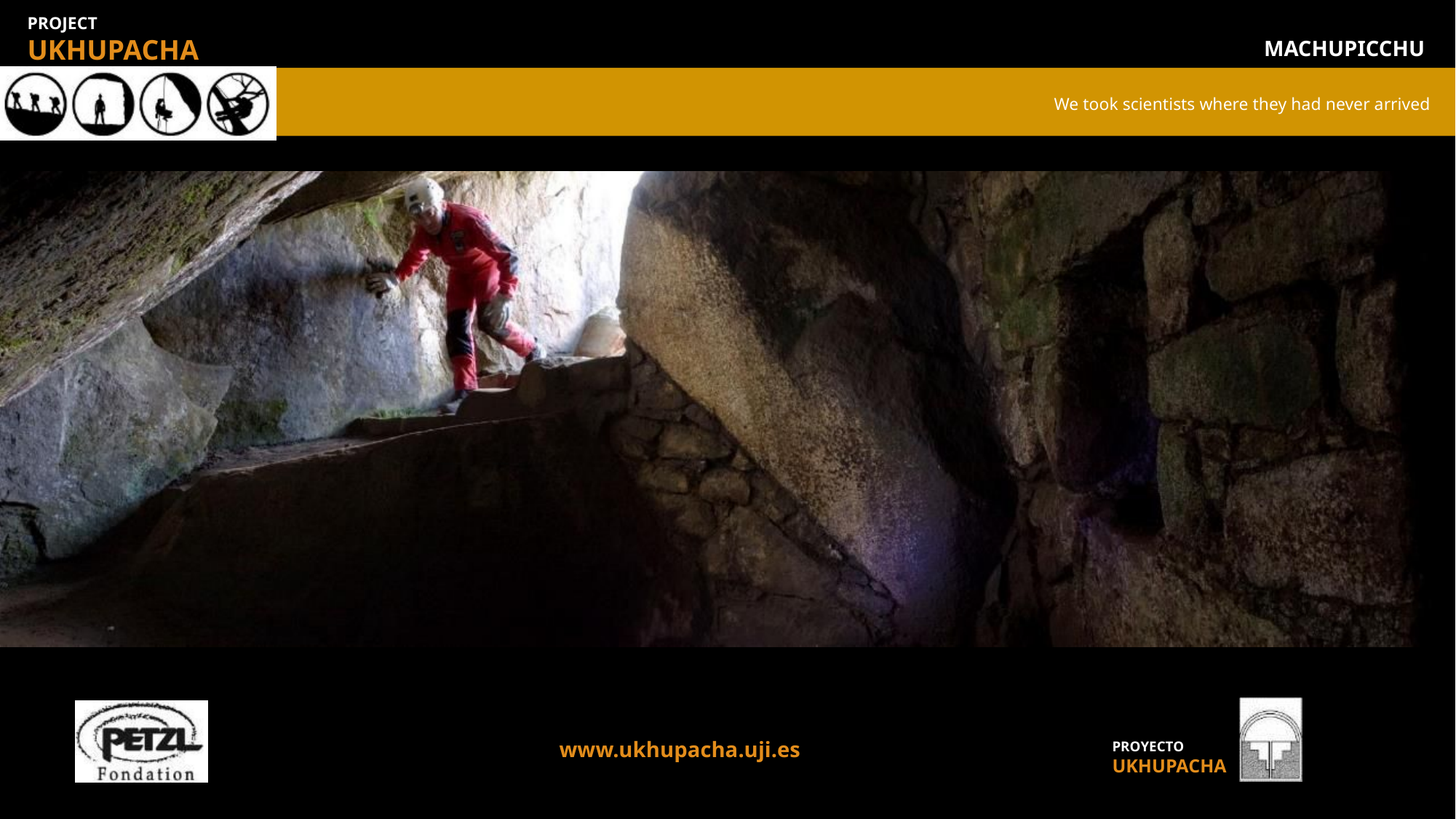

## Slide 15
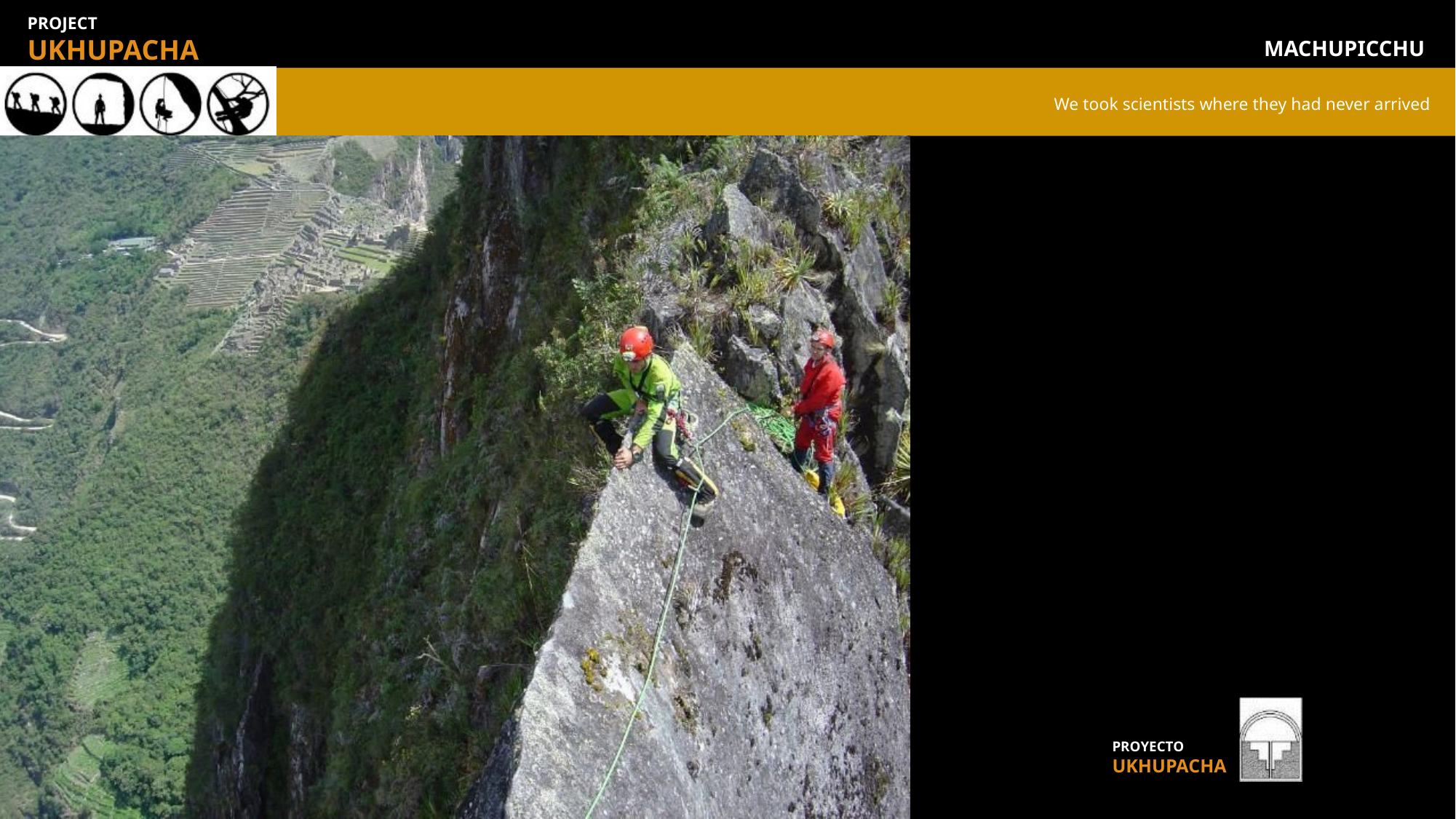

## Slide 16
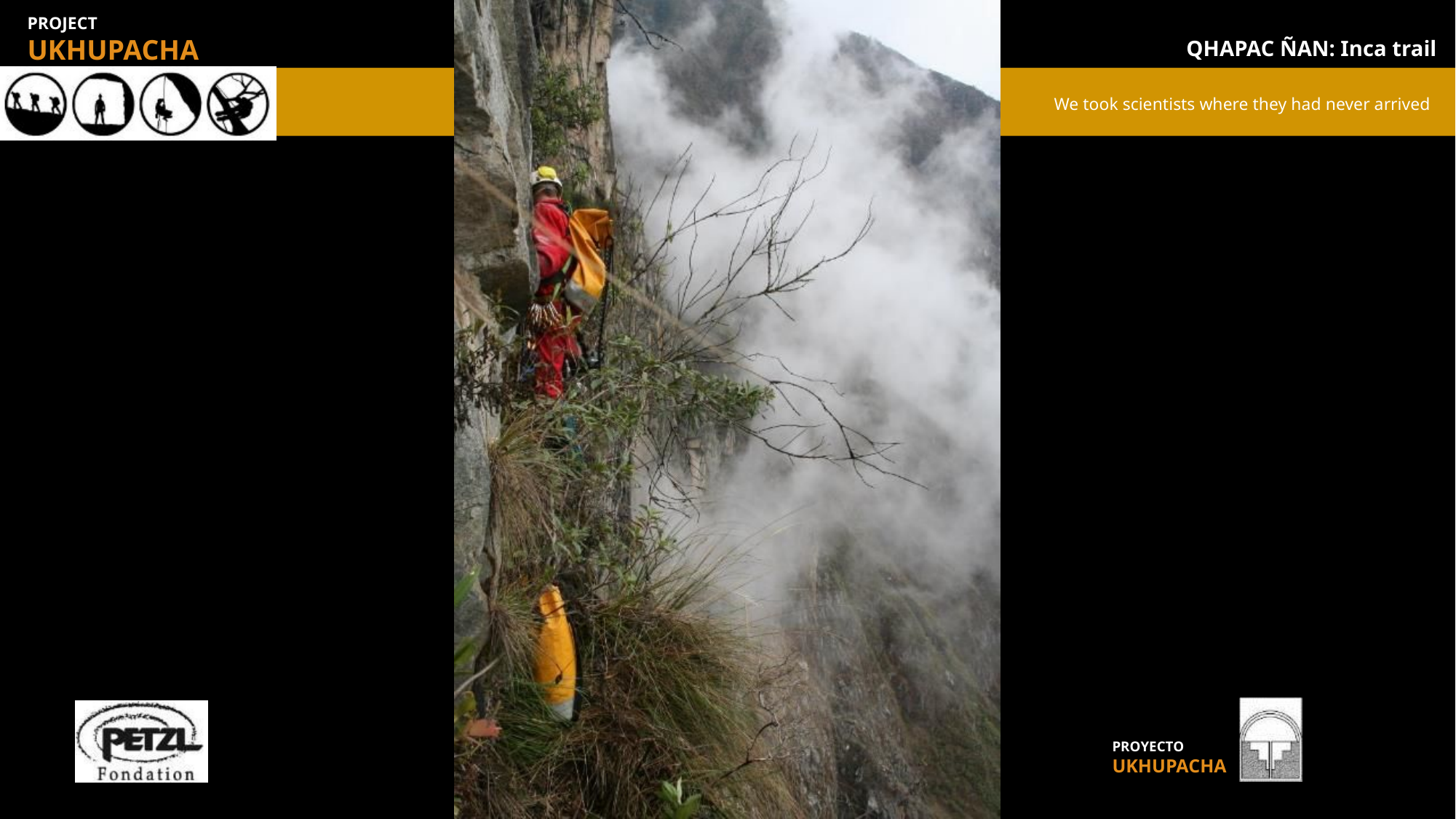

## Slide 17
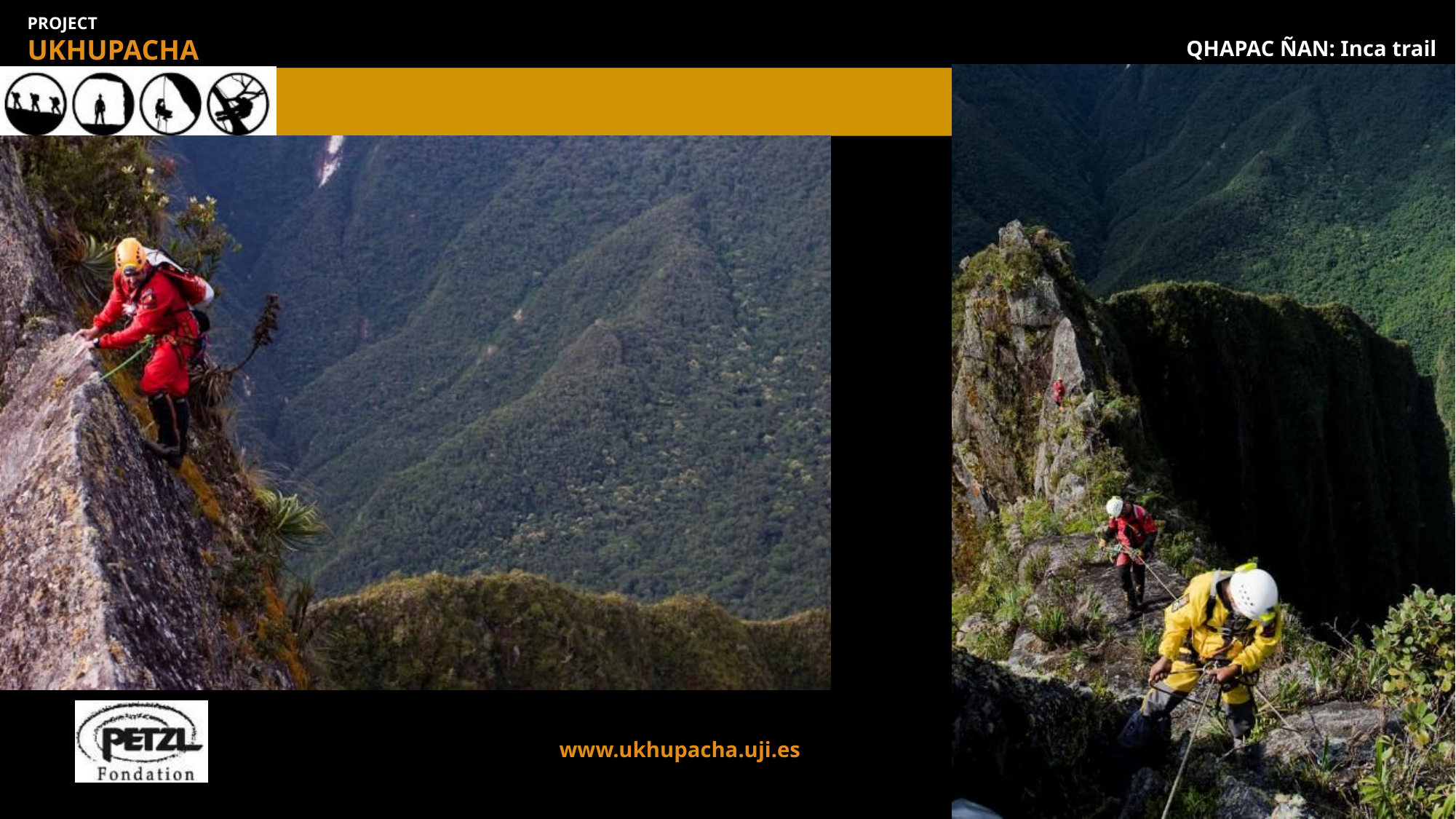

## Slide 18
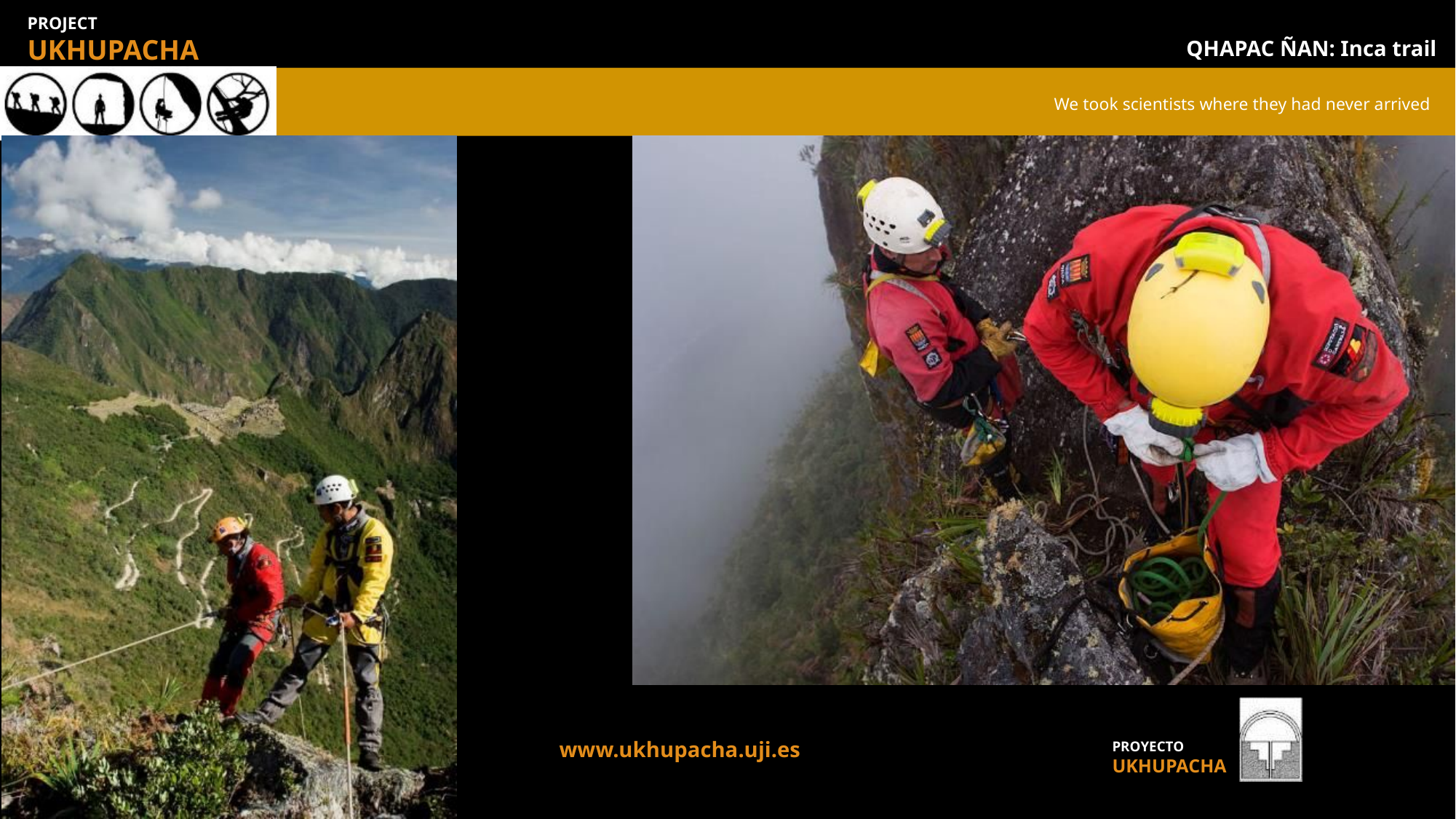

## Slide 19
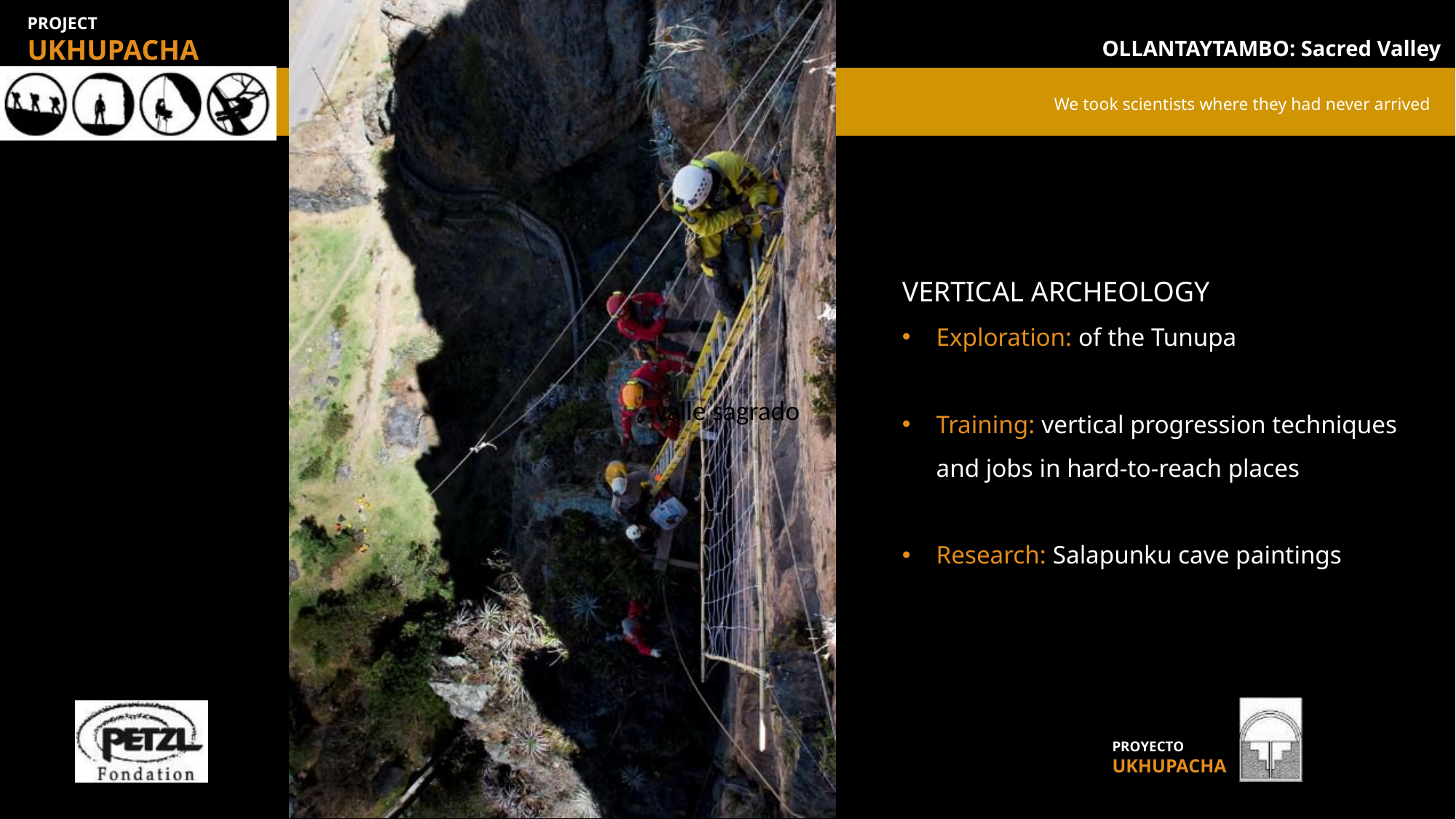

## Slide 20
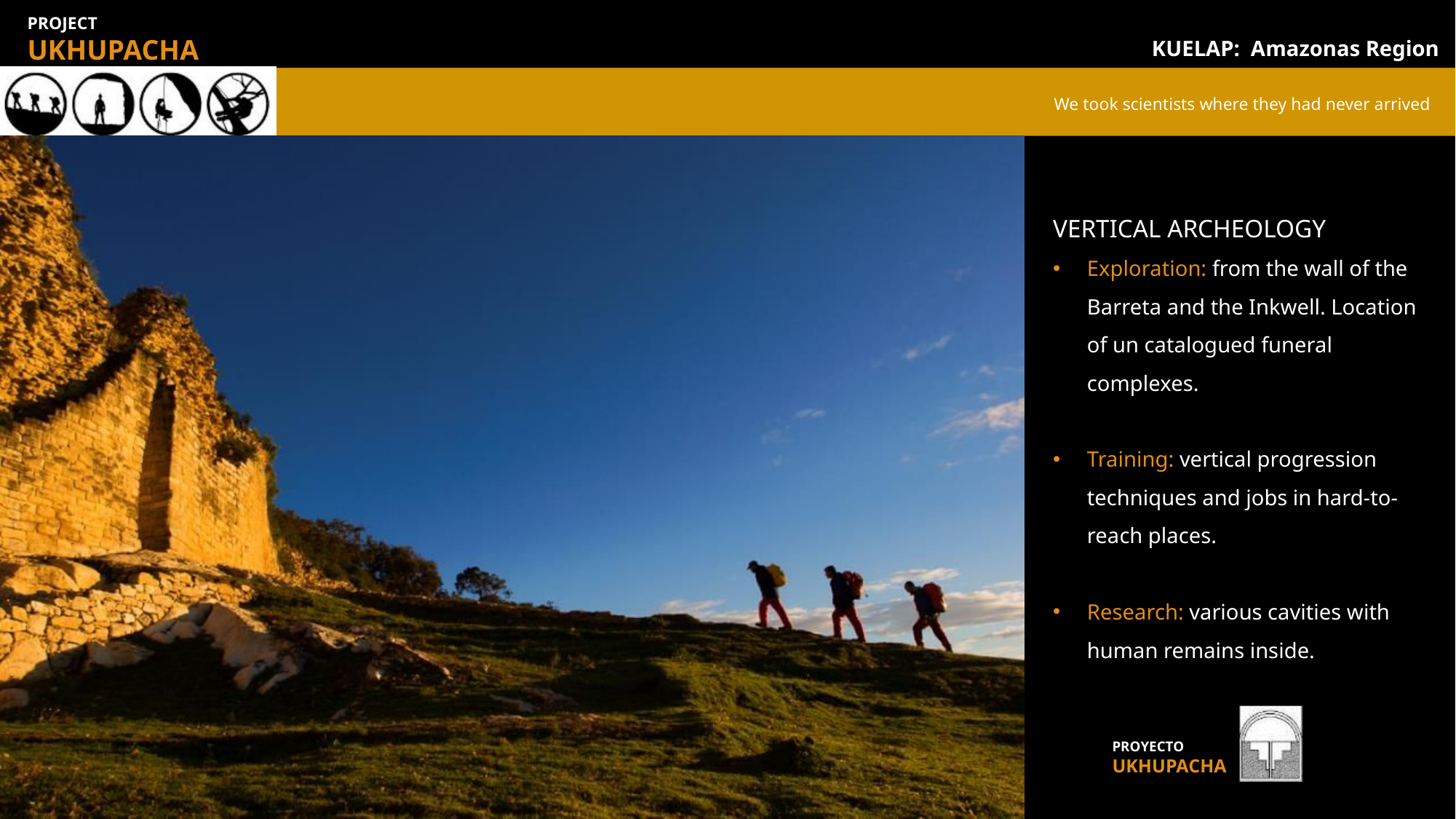

## Slide 21
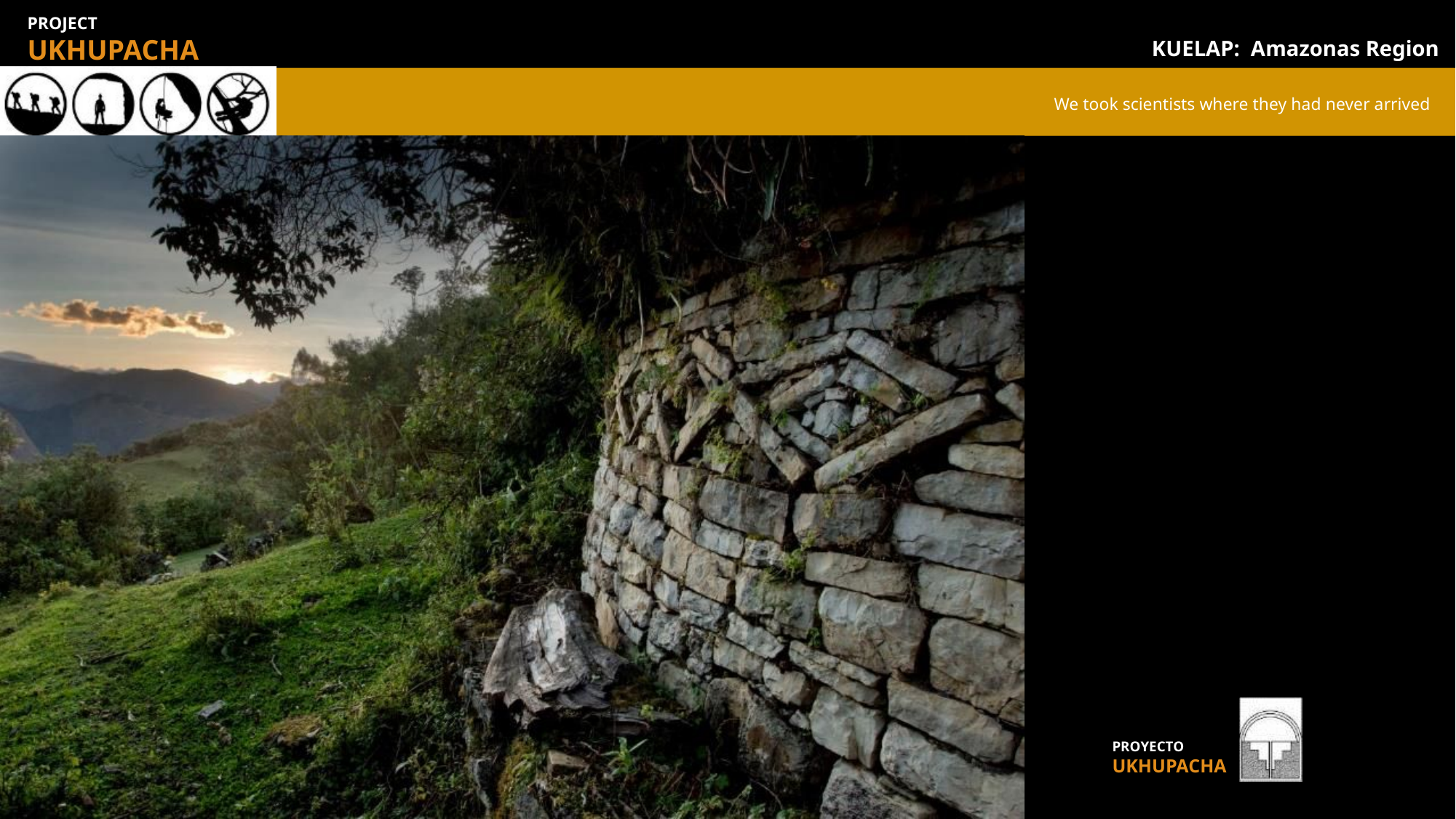

## Slide 22
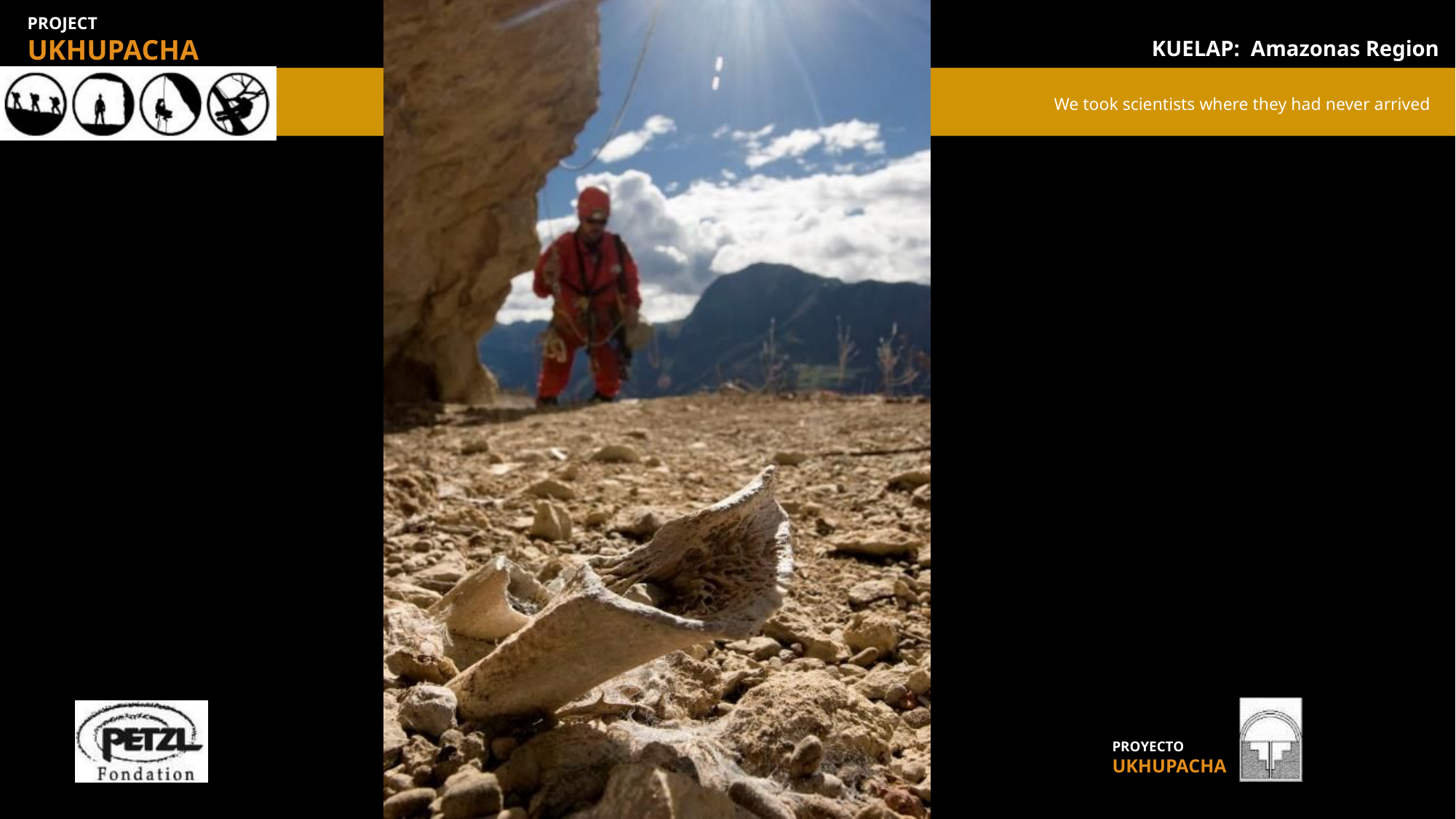

## Slide 23
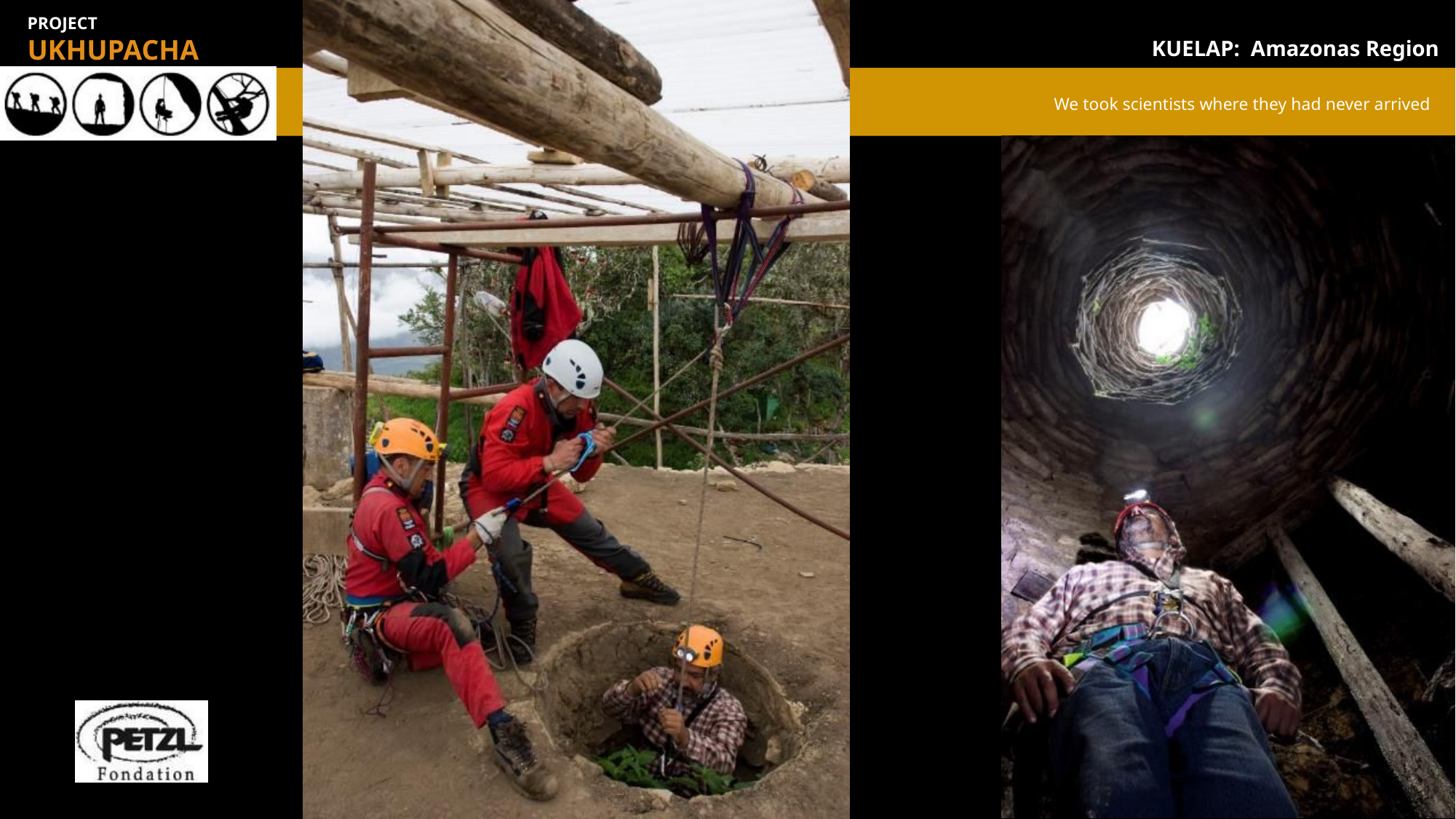

## Slide 24
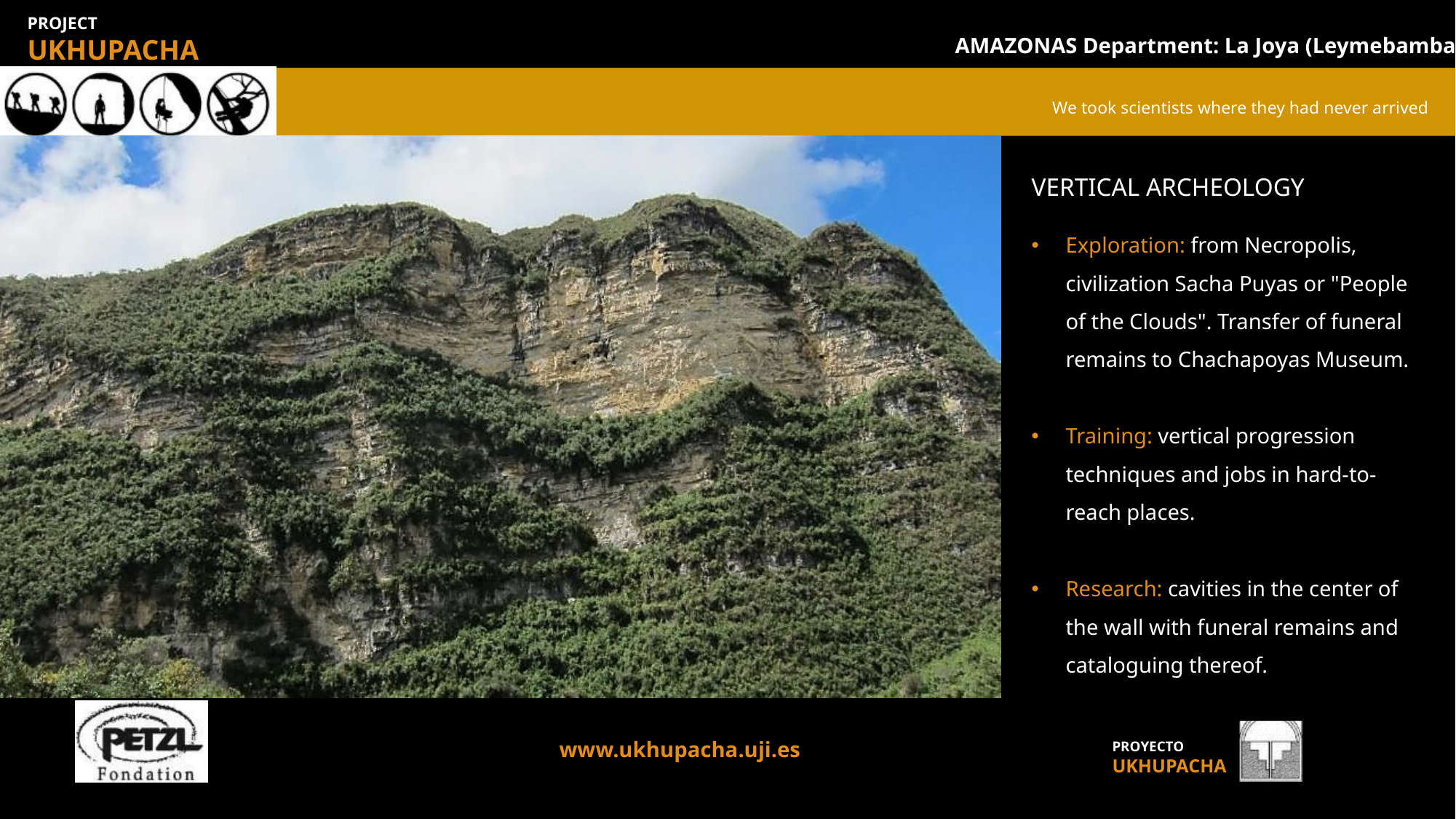

## Slide 25
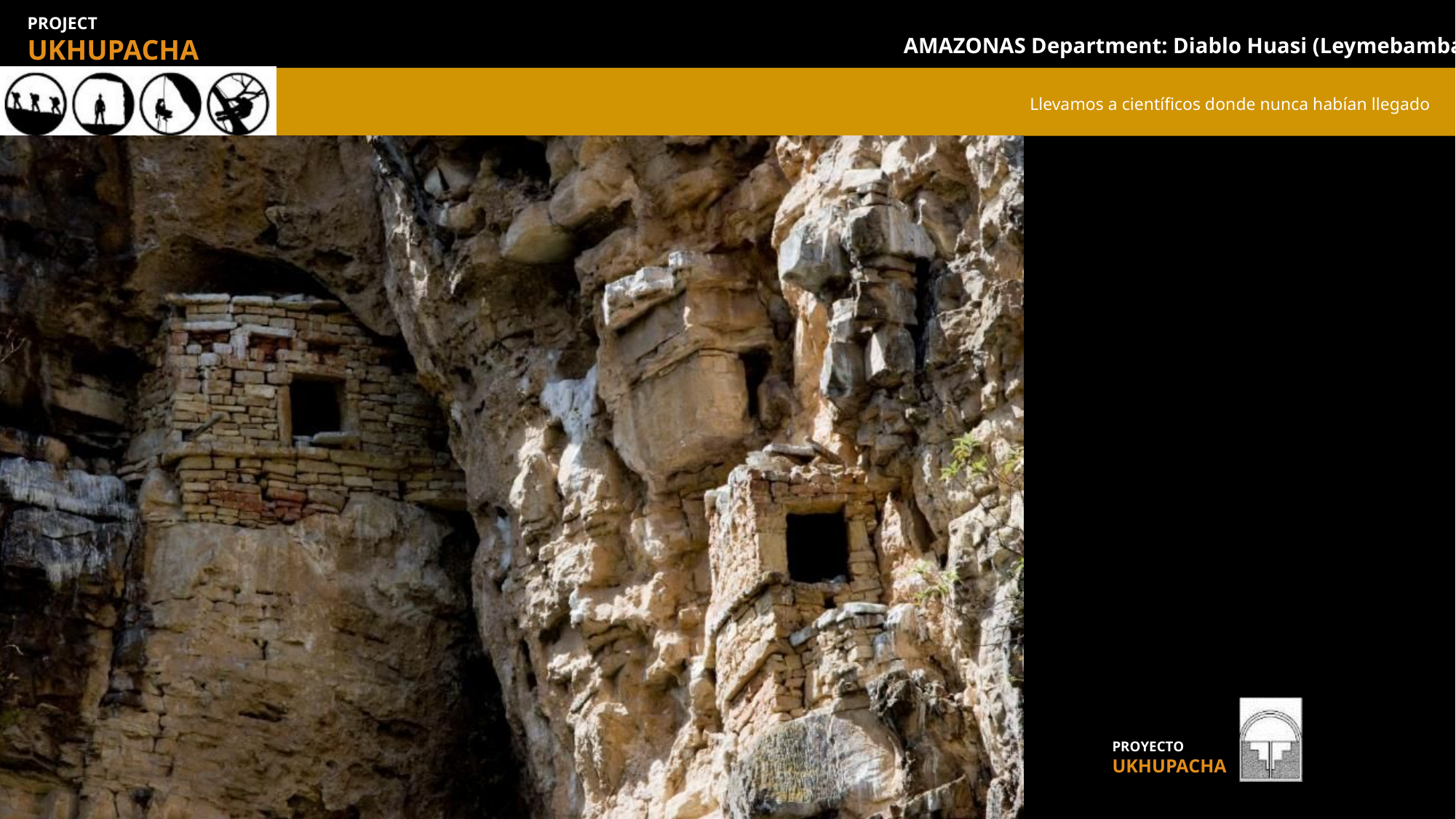

## Slide 26
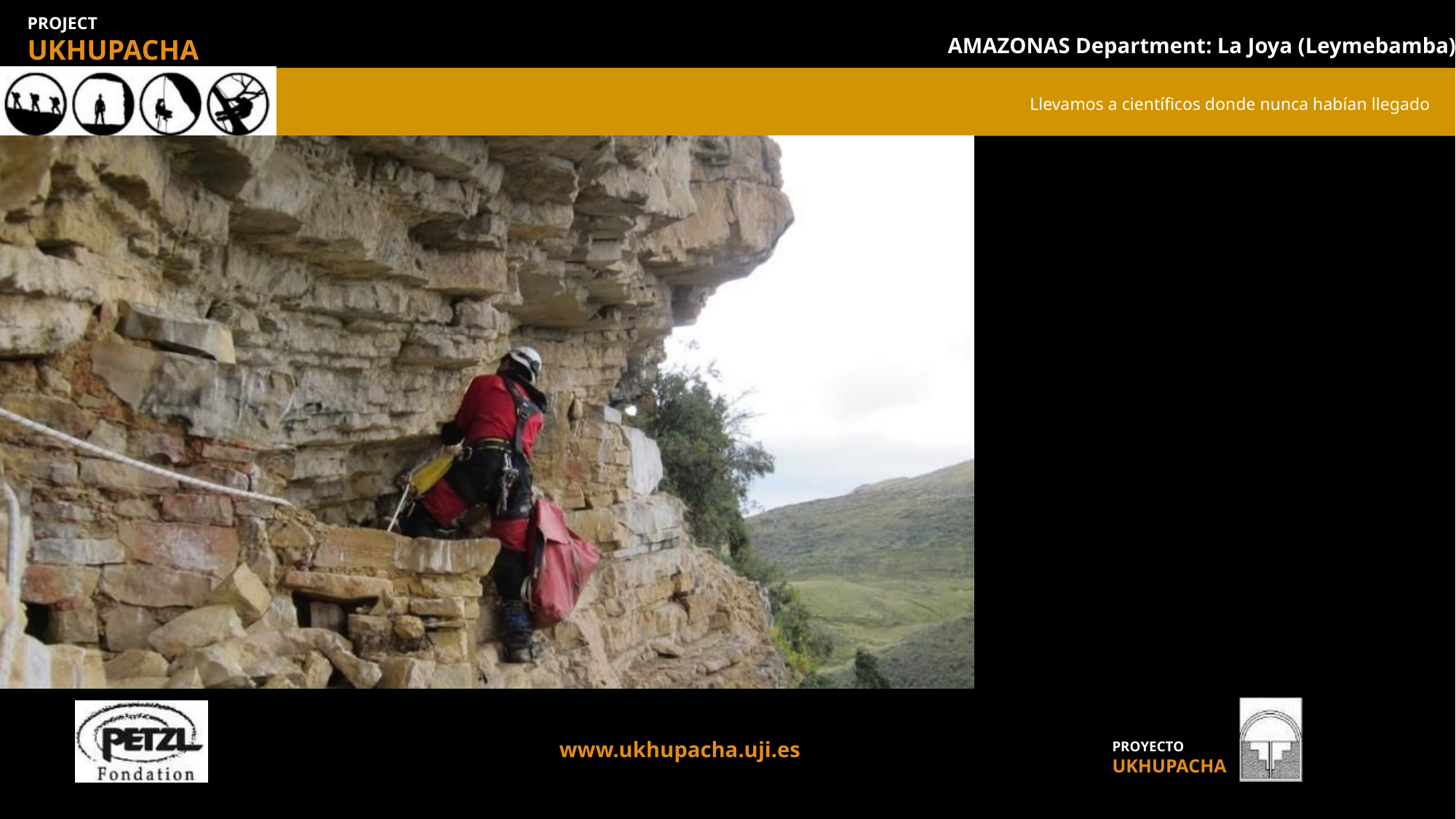

## Slide 27
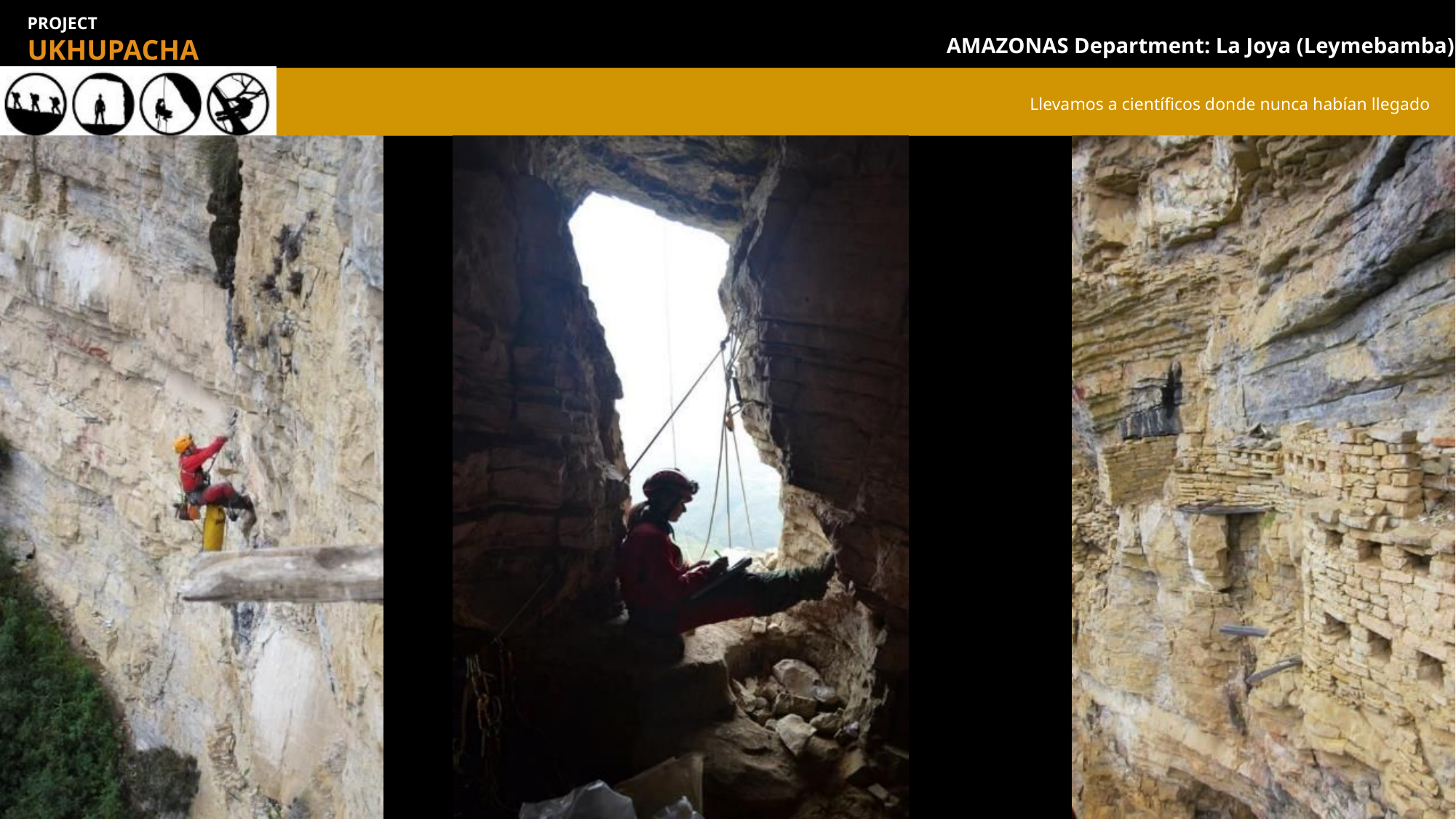

## Slide 28
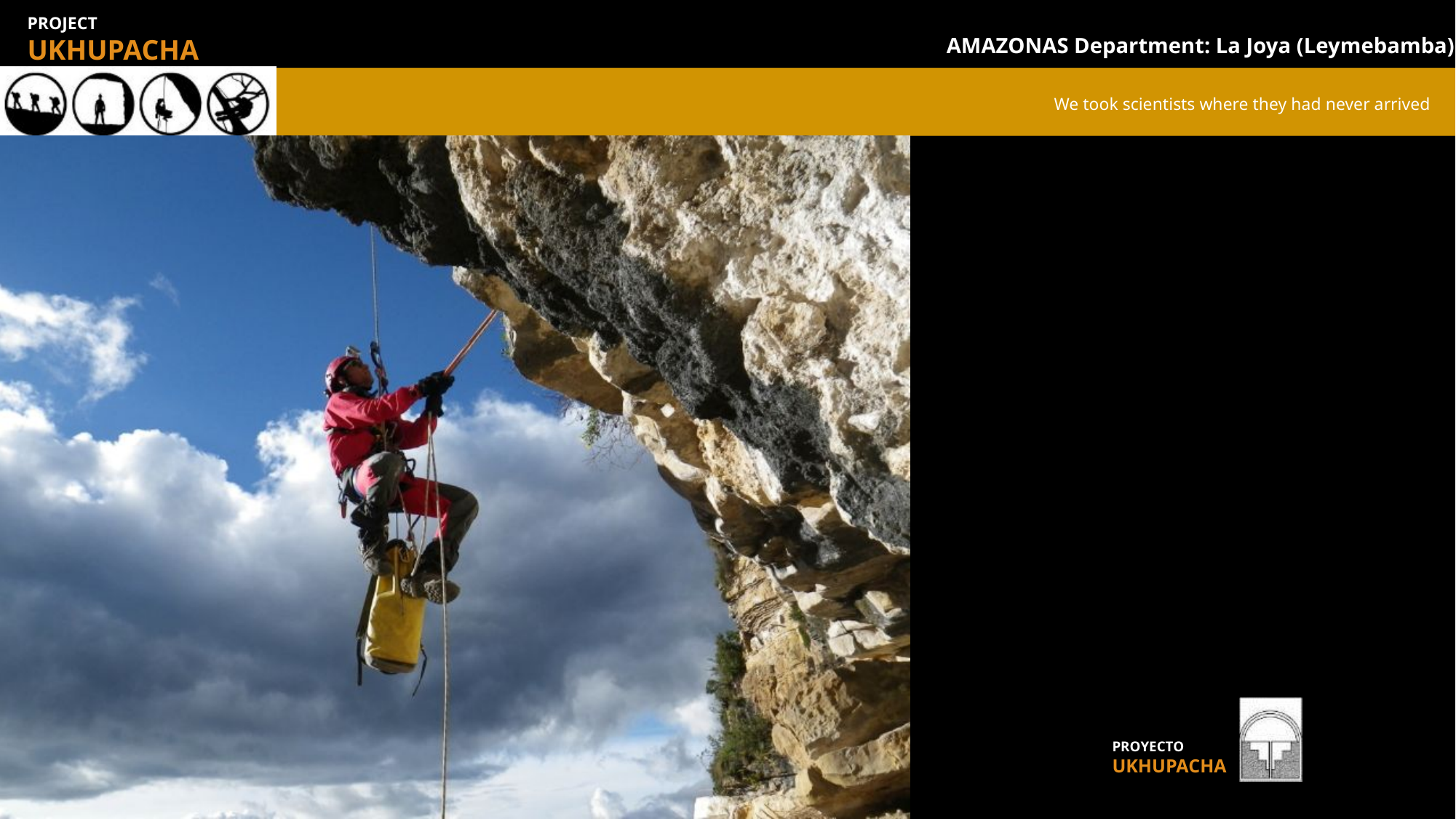

## Slide 29
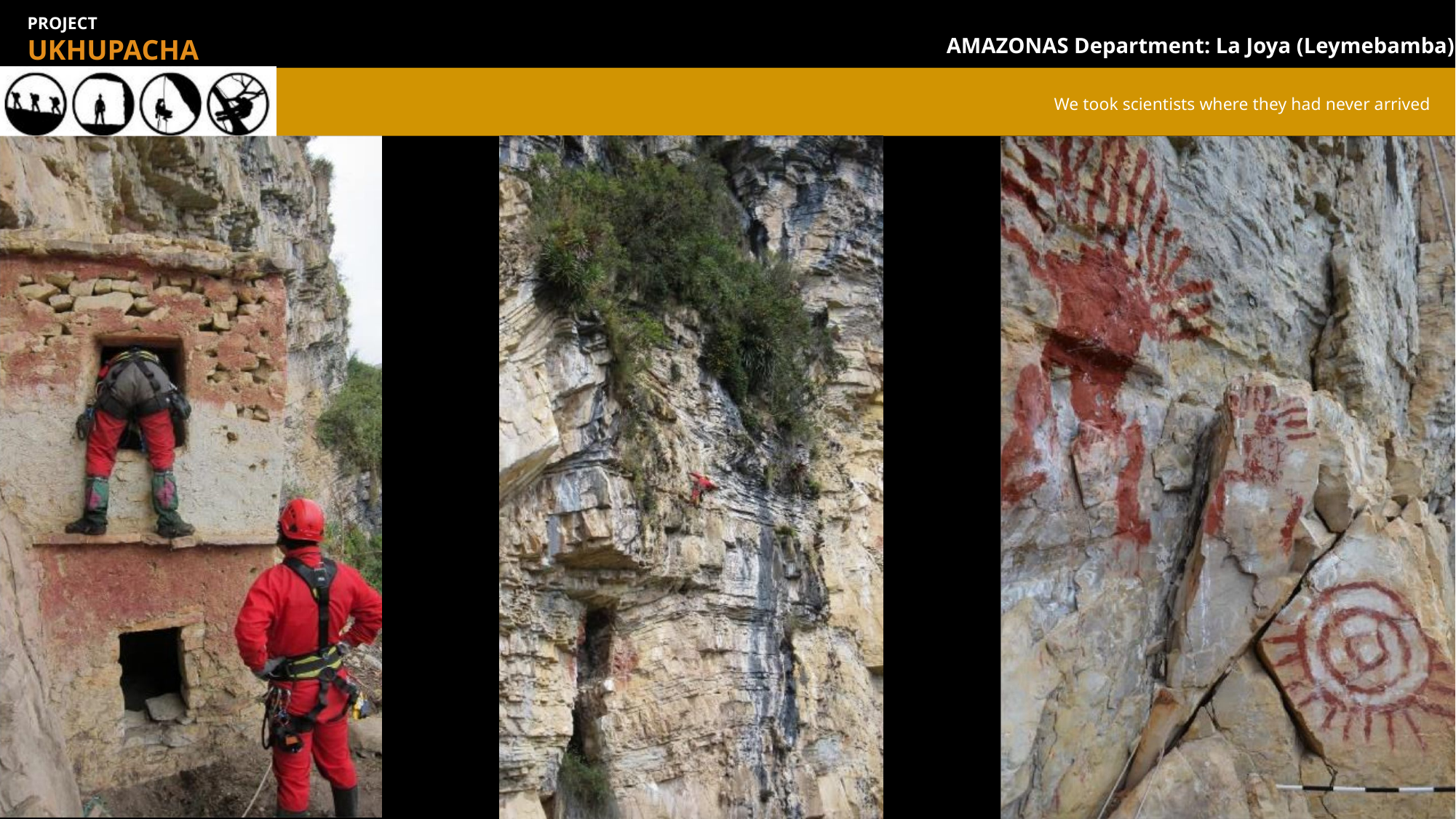

## Slide 30
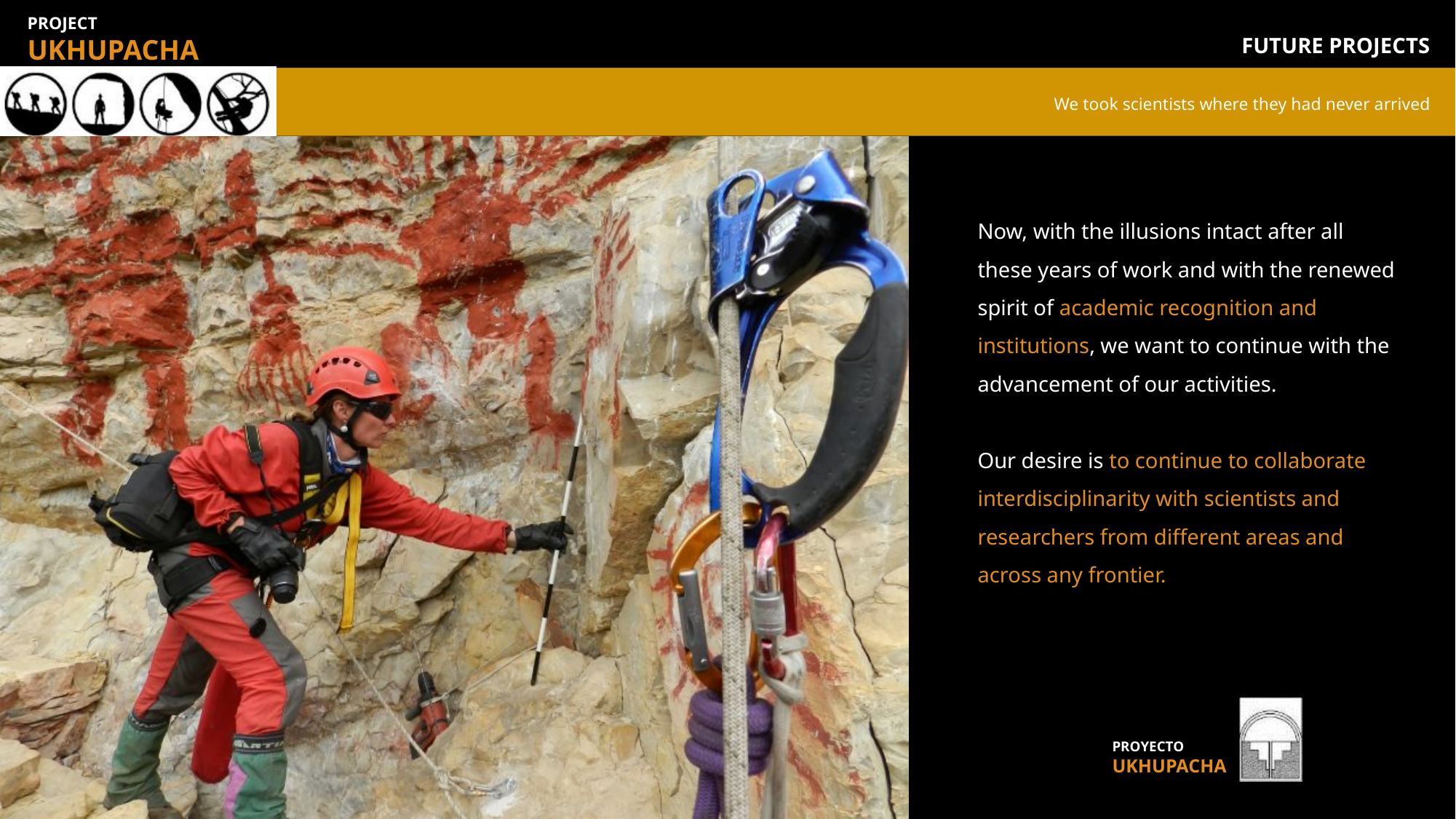

## Slide 31
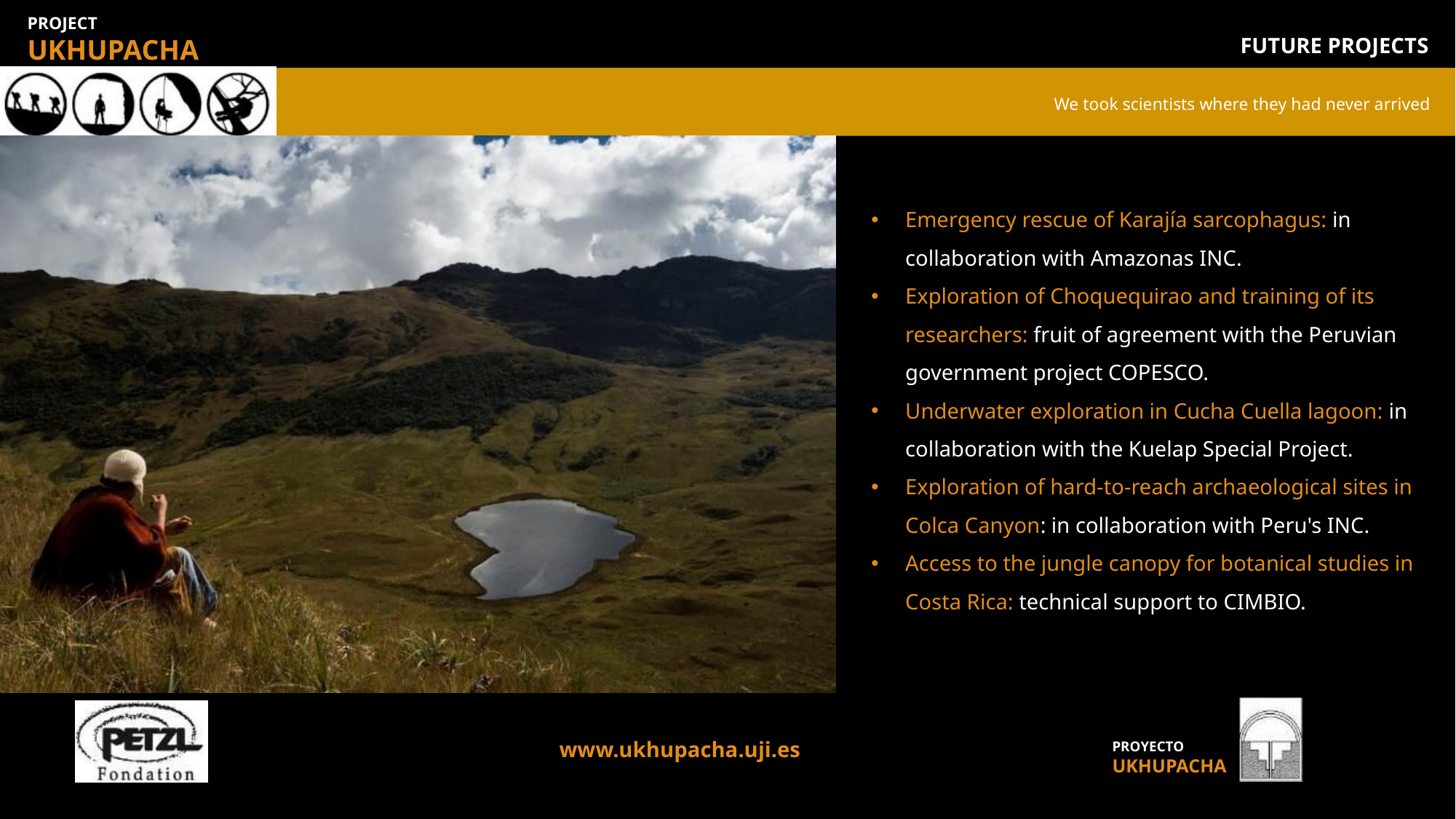

## Slide 32
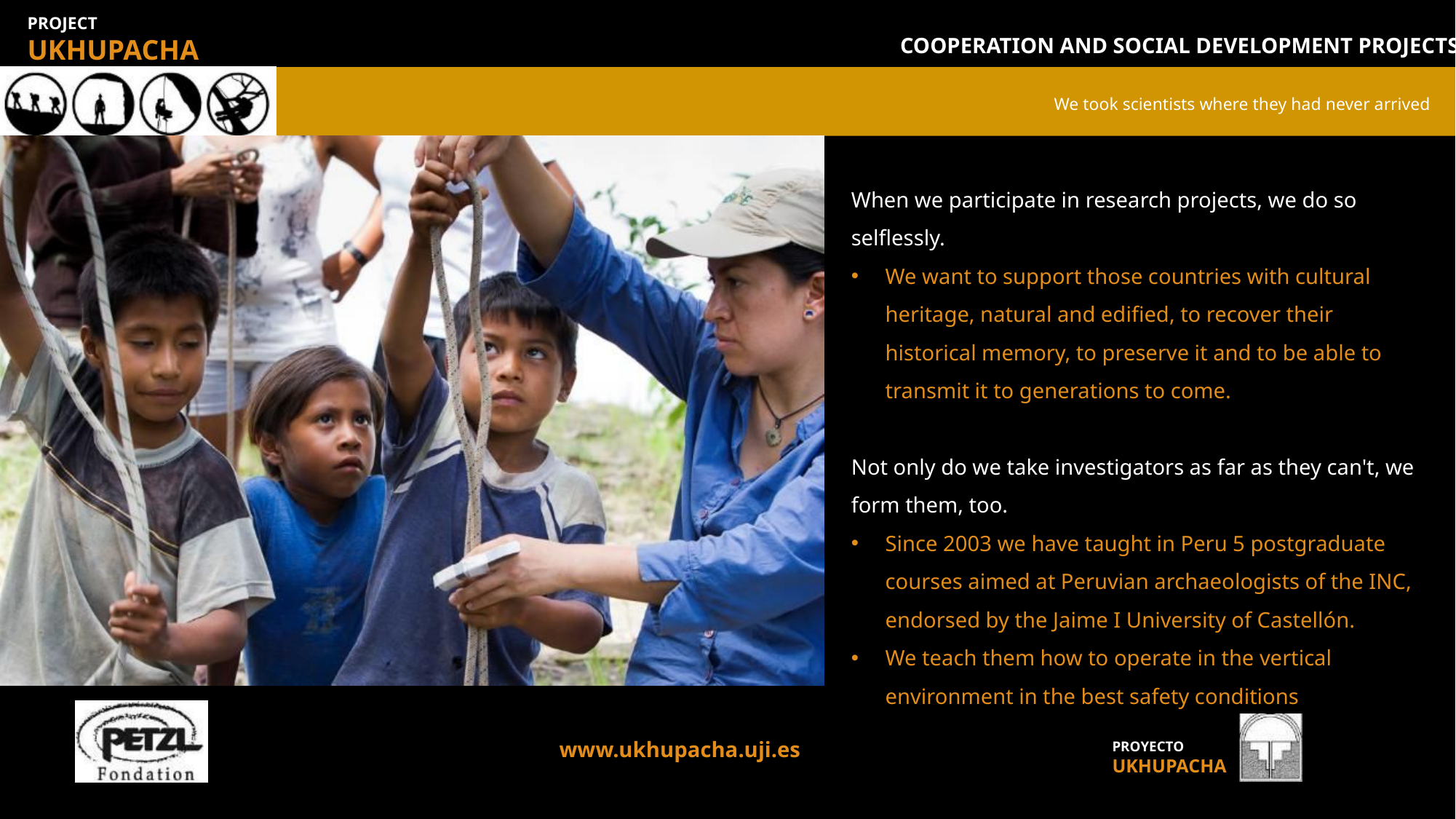

## Slide 33
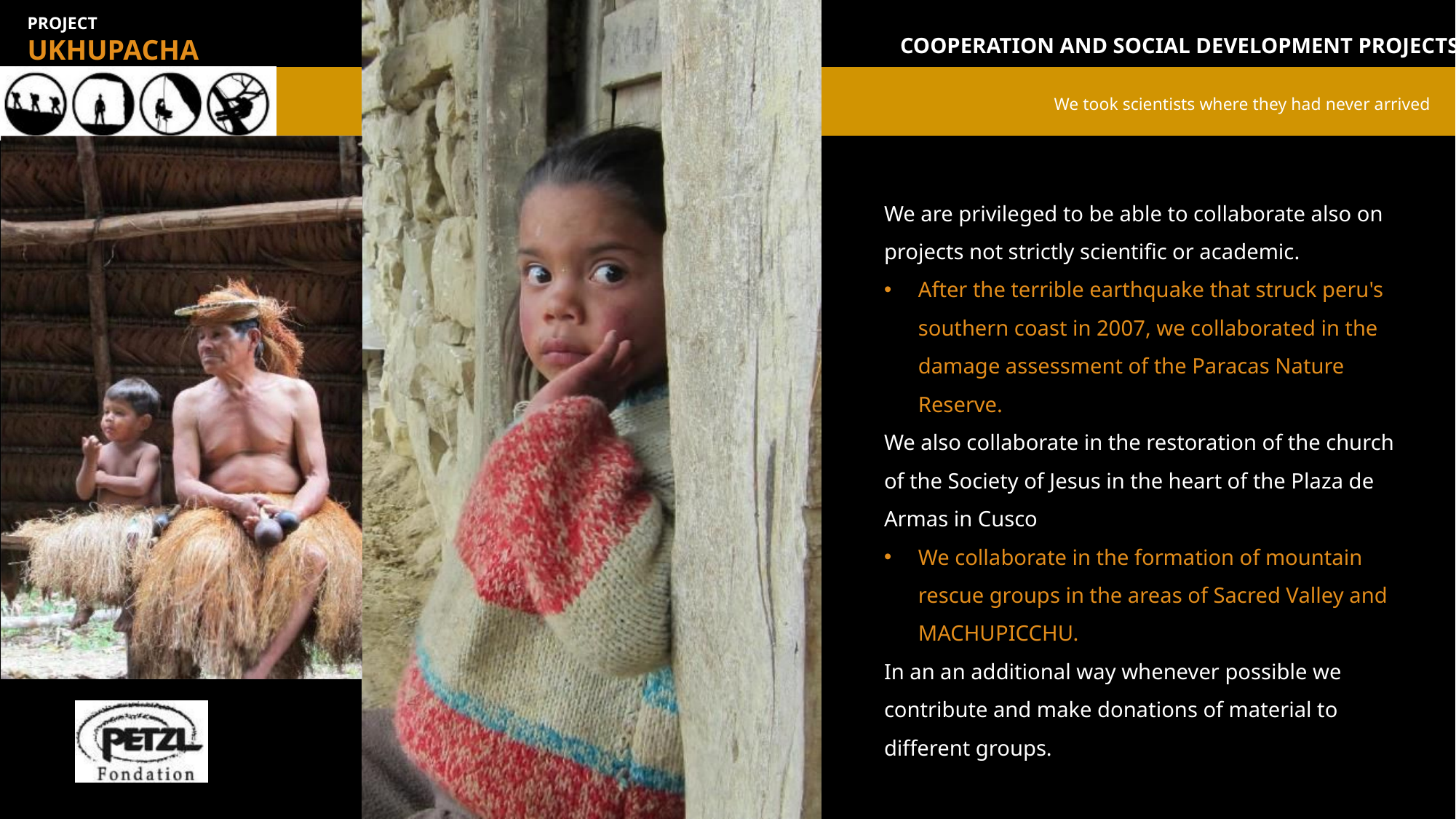

## Slide 34
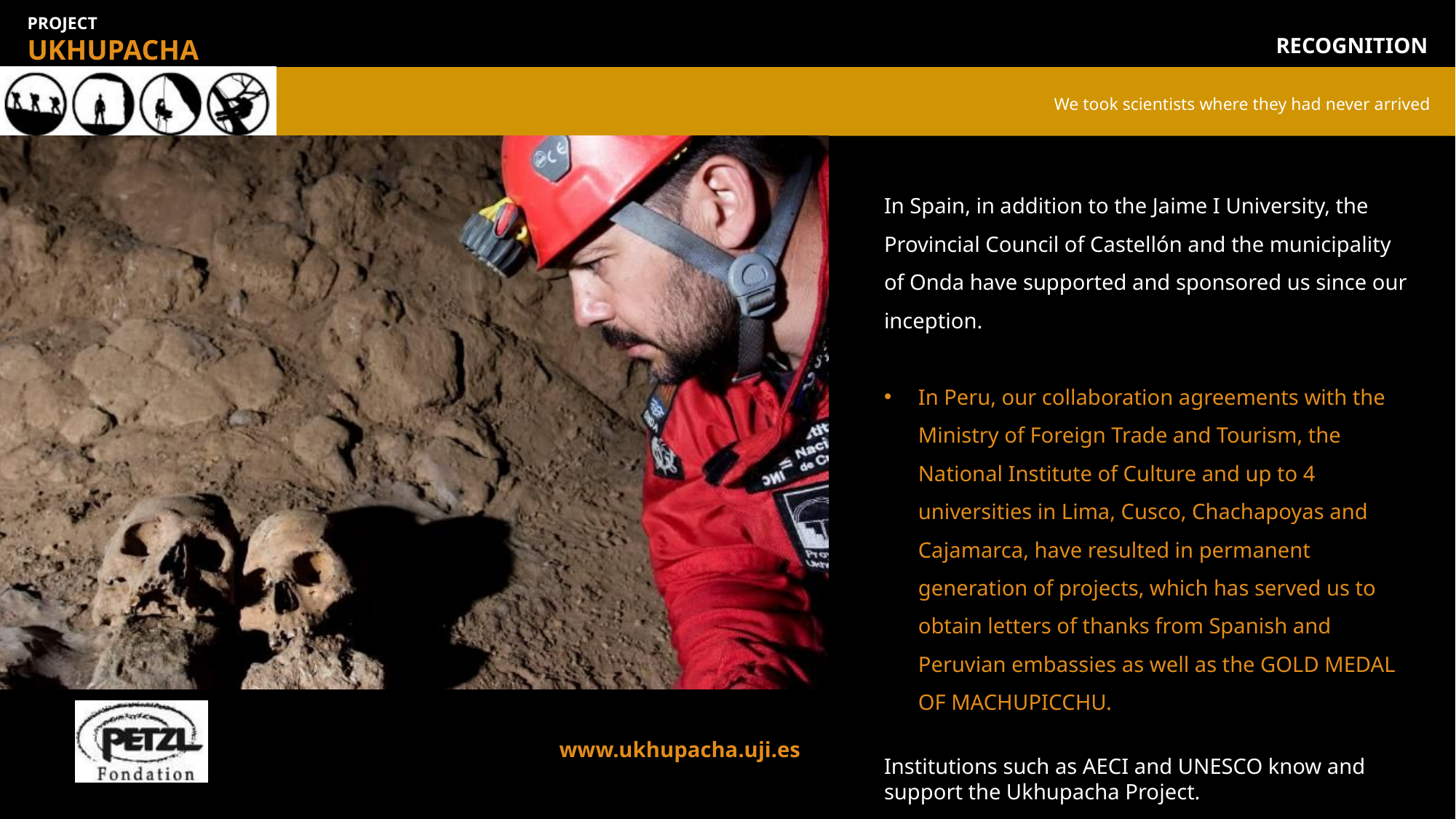

## Slide 35
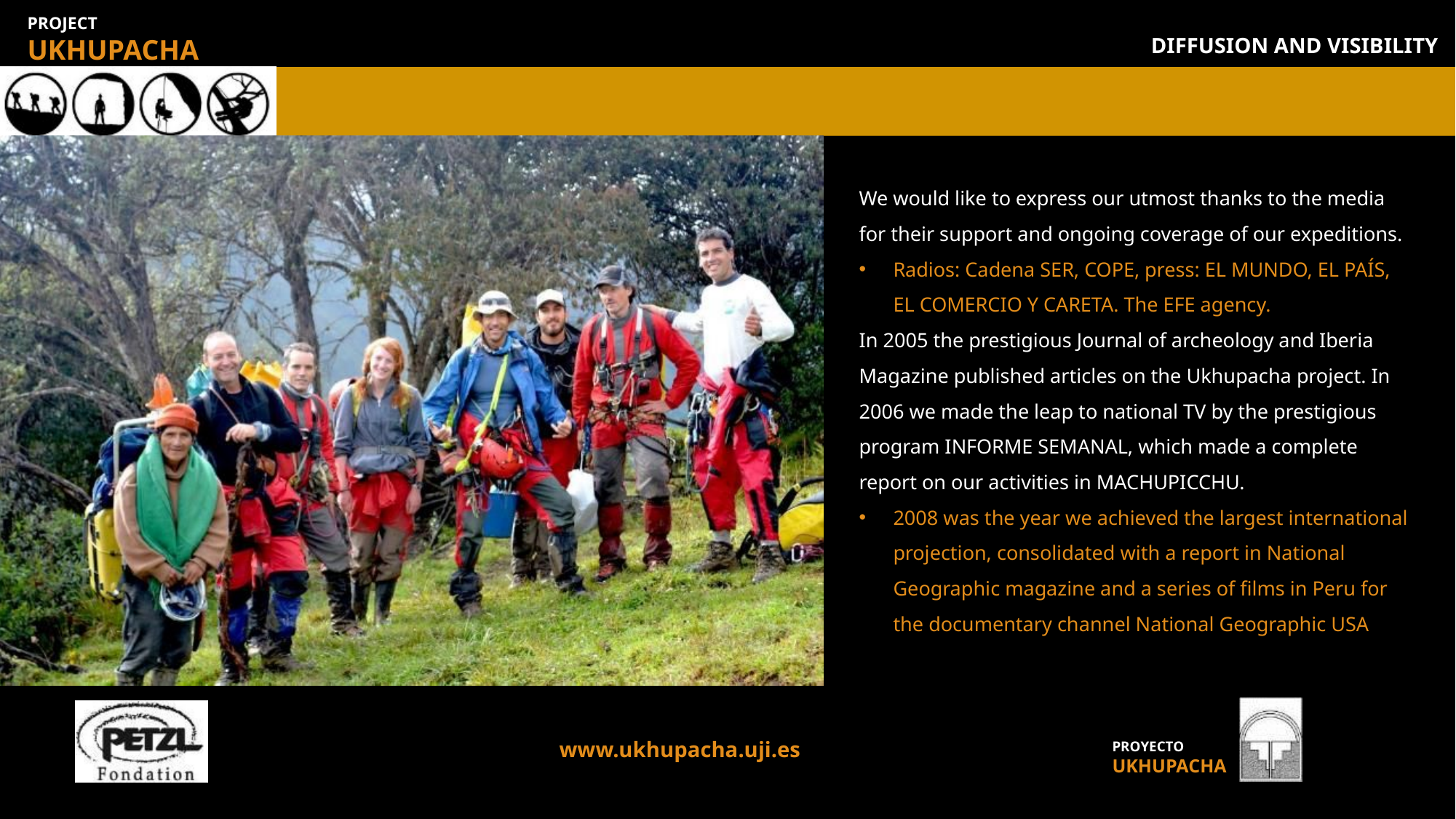

## Slide 36
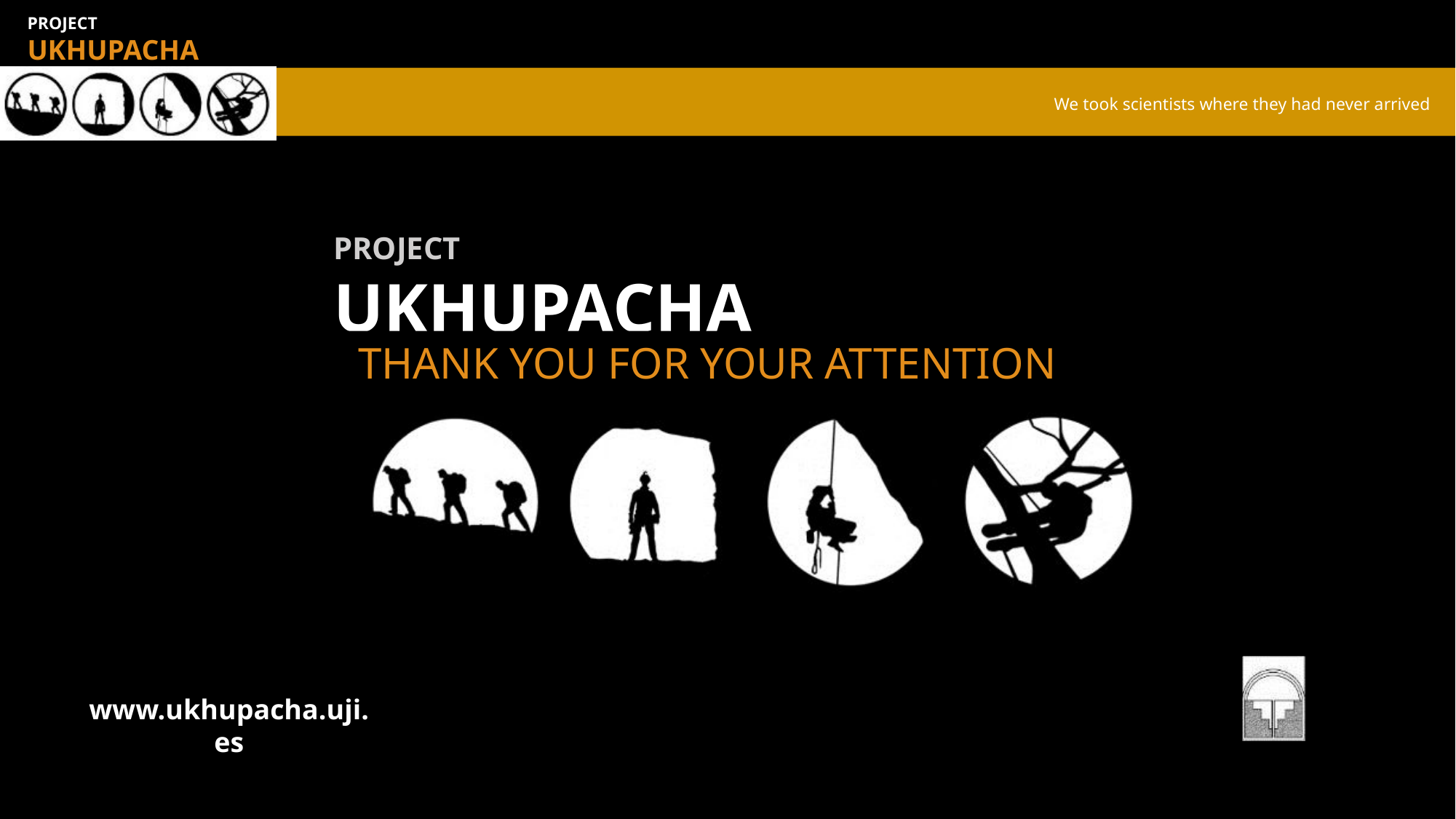

## Slide 37
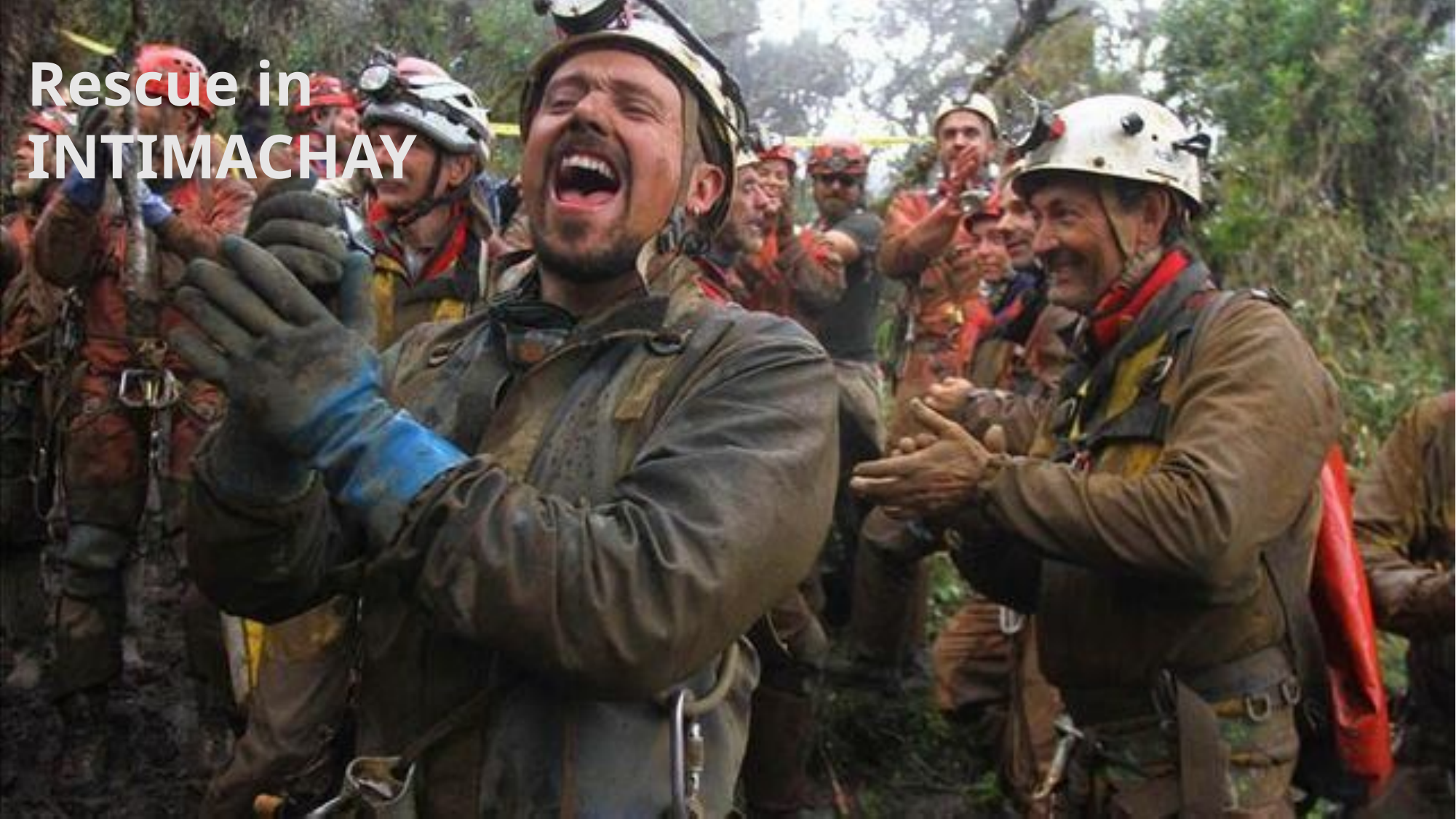

## Slide 38
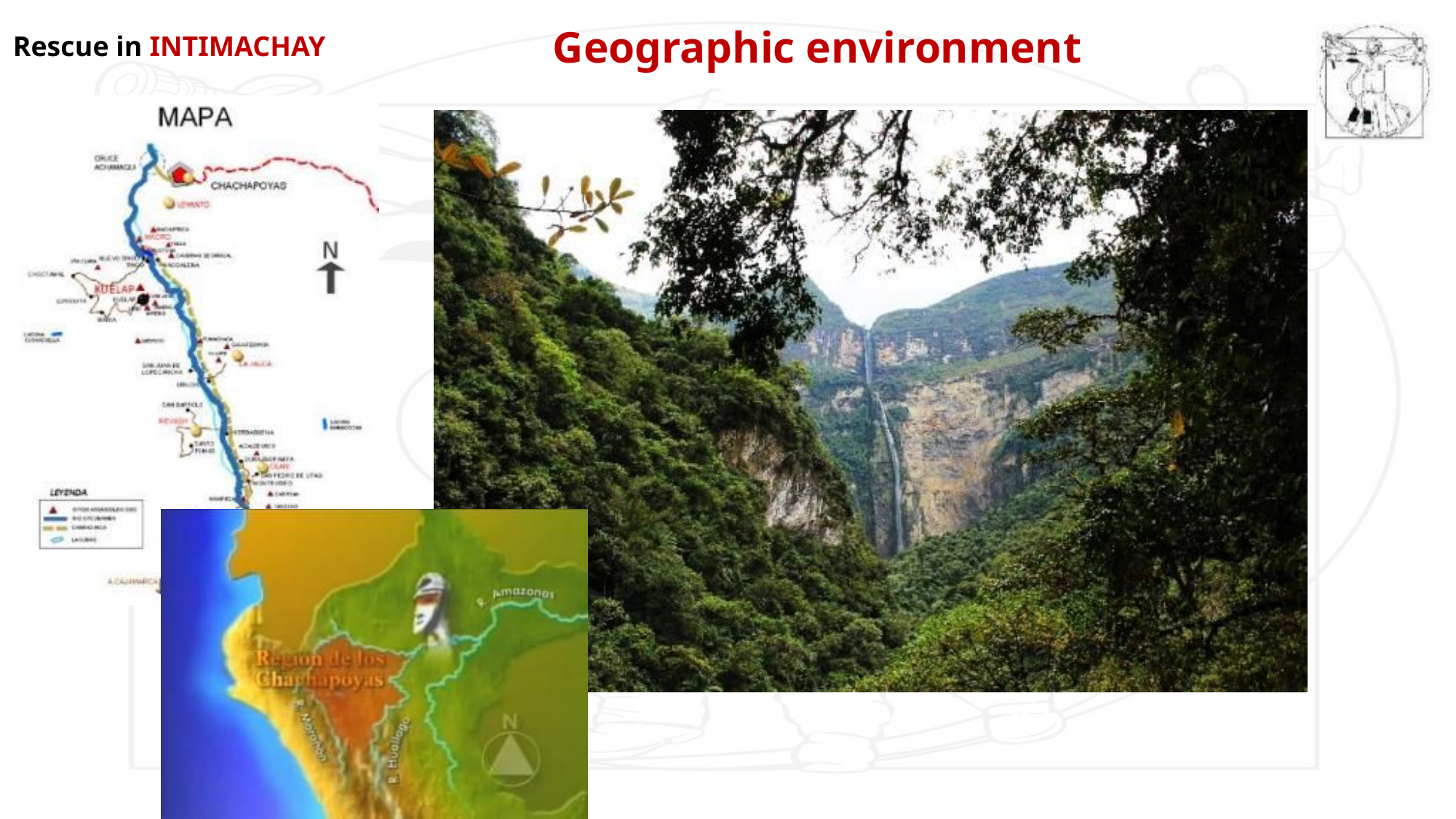

## Slide 39
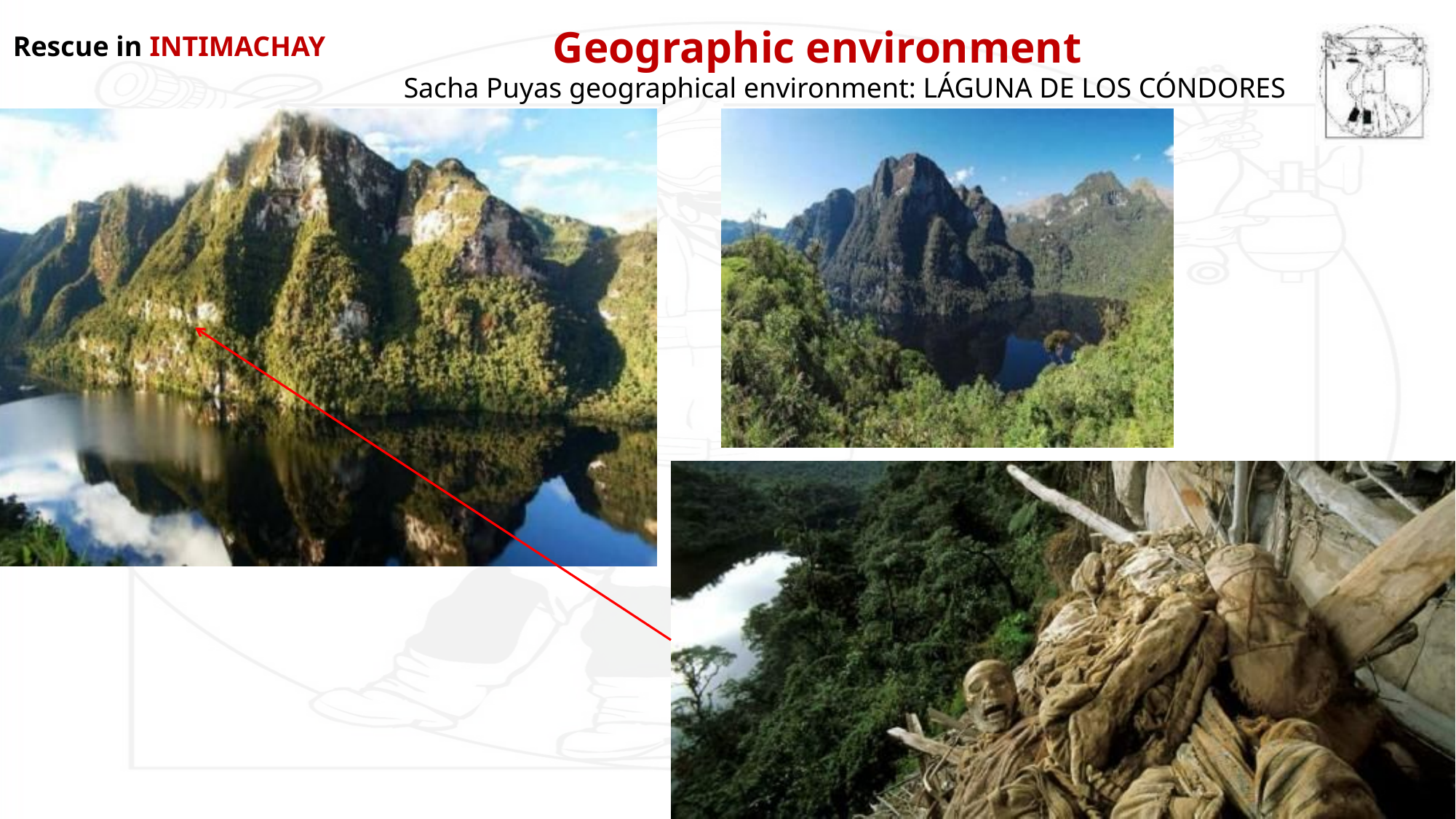

## Slide 40
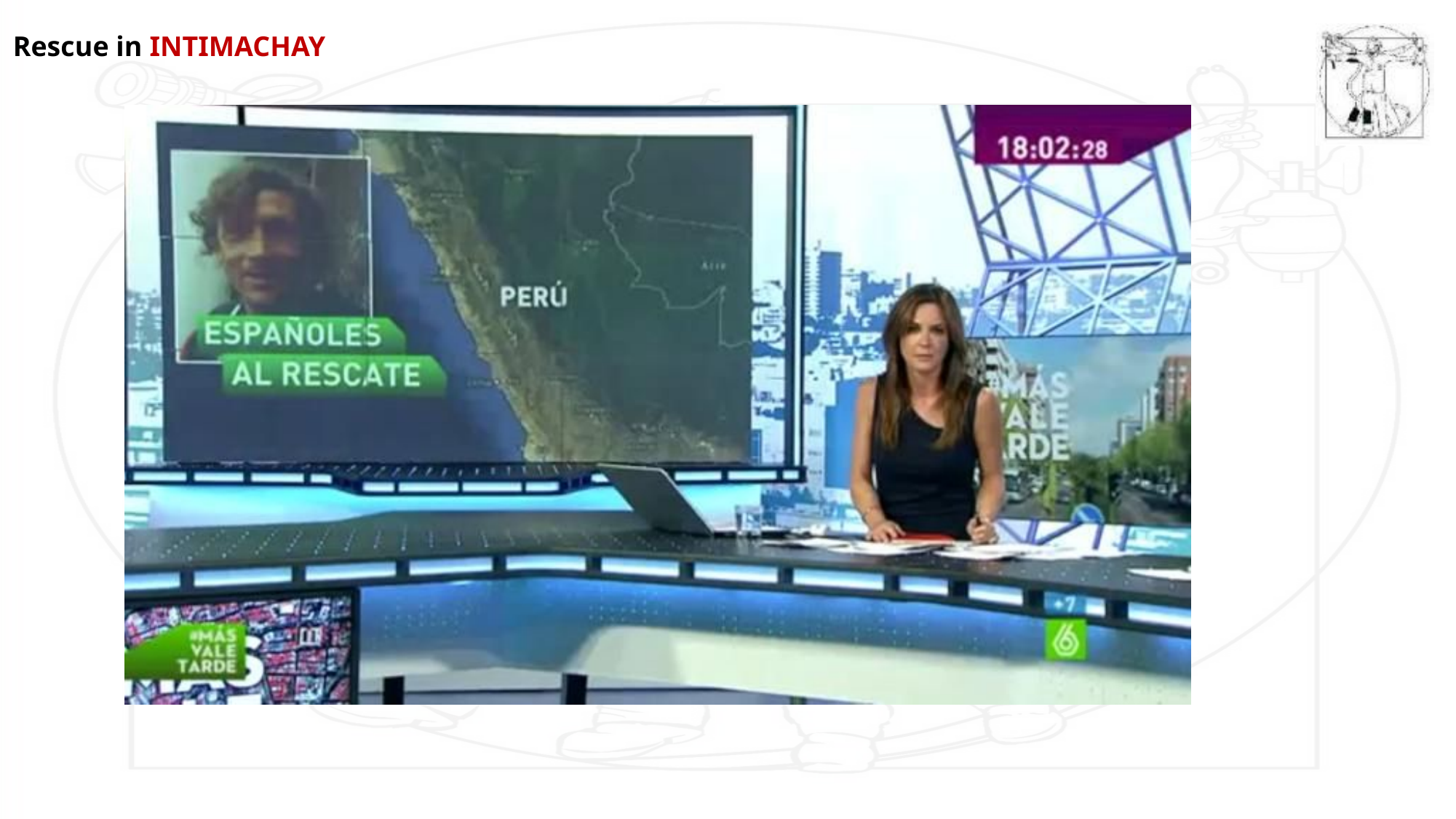

## Slide 41
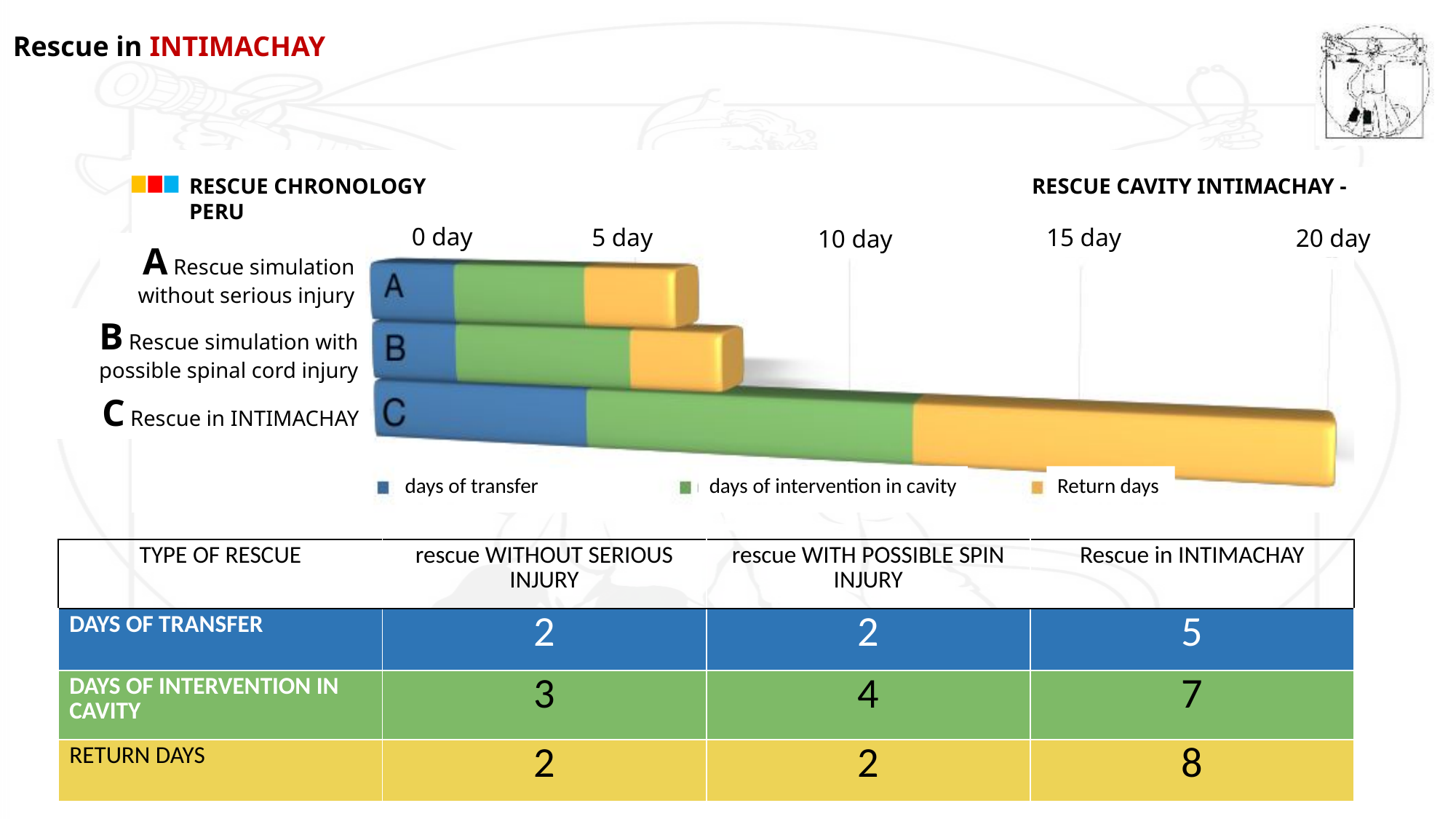

## Slide 42
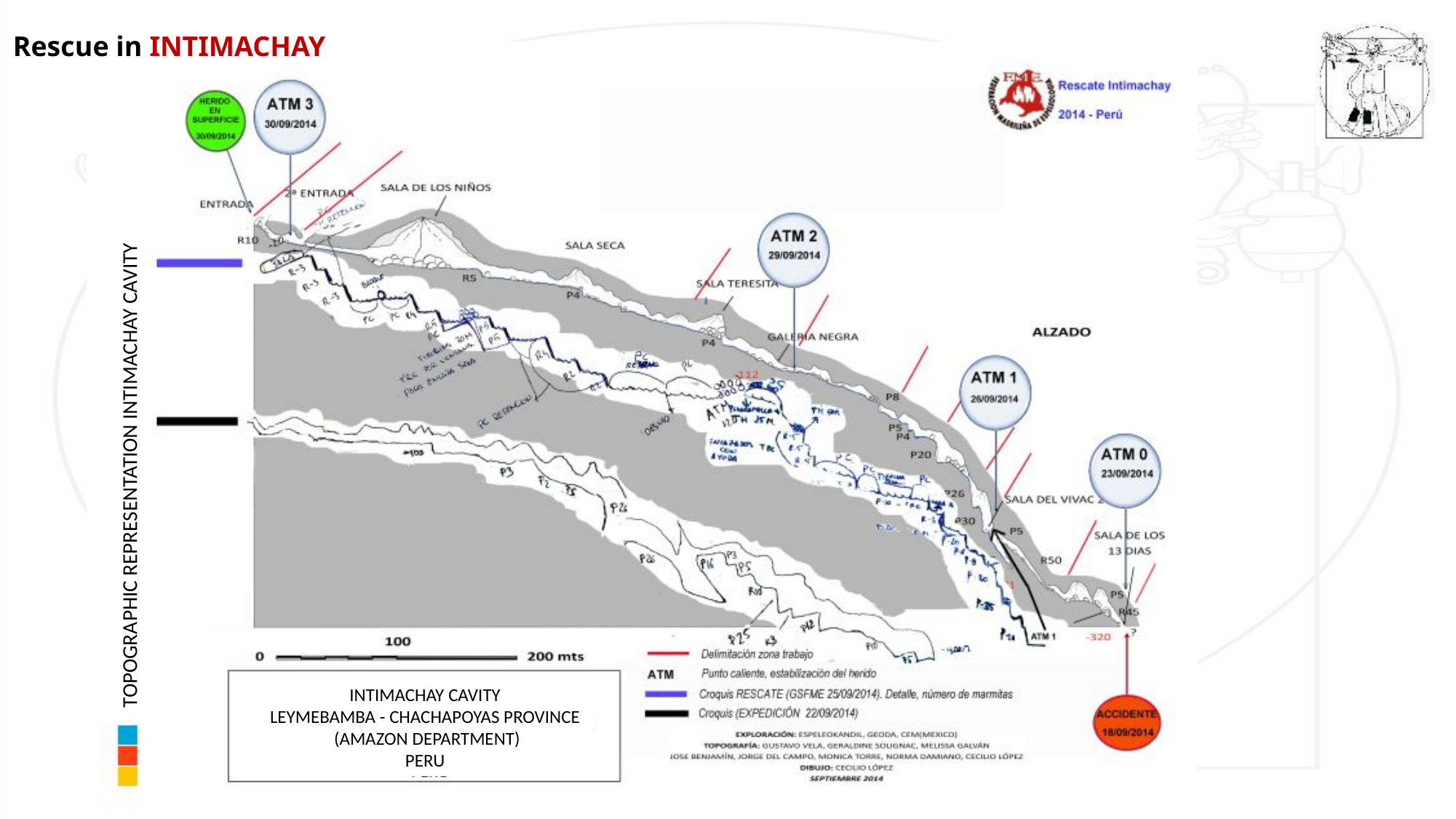

## Slide 43
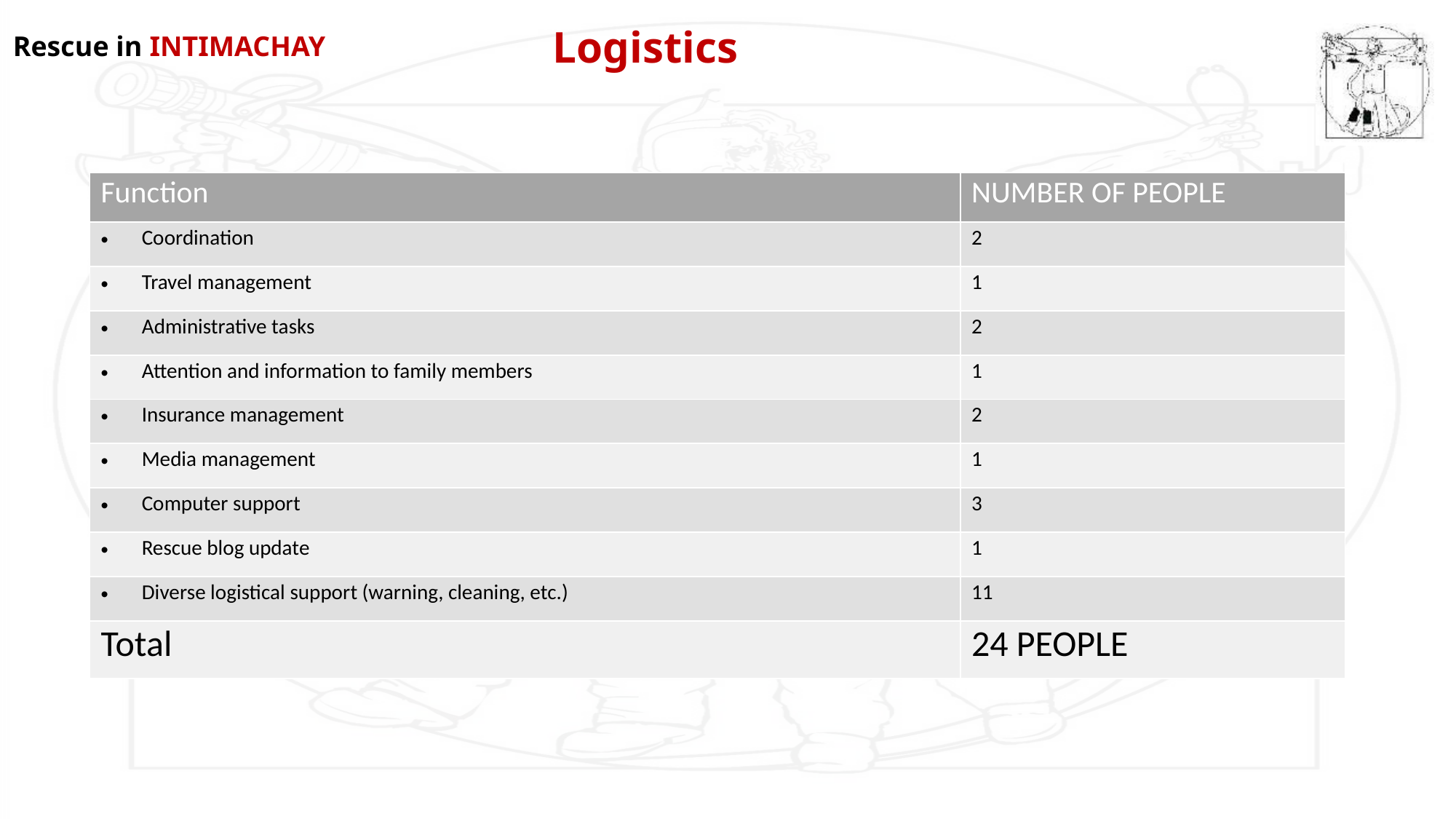

## Slide 44
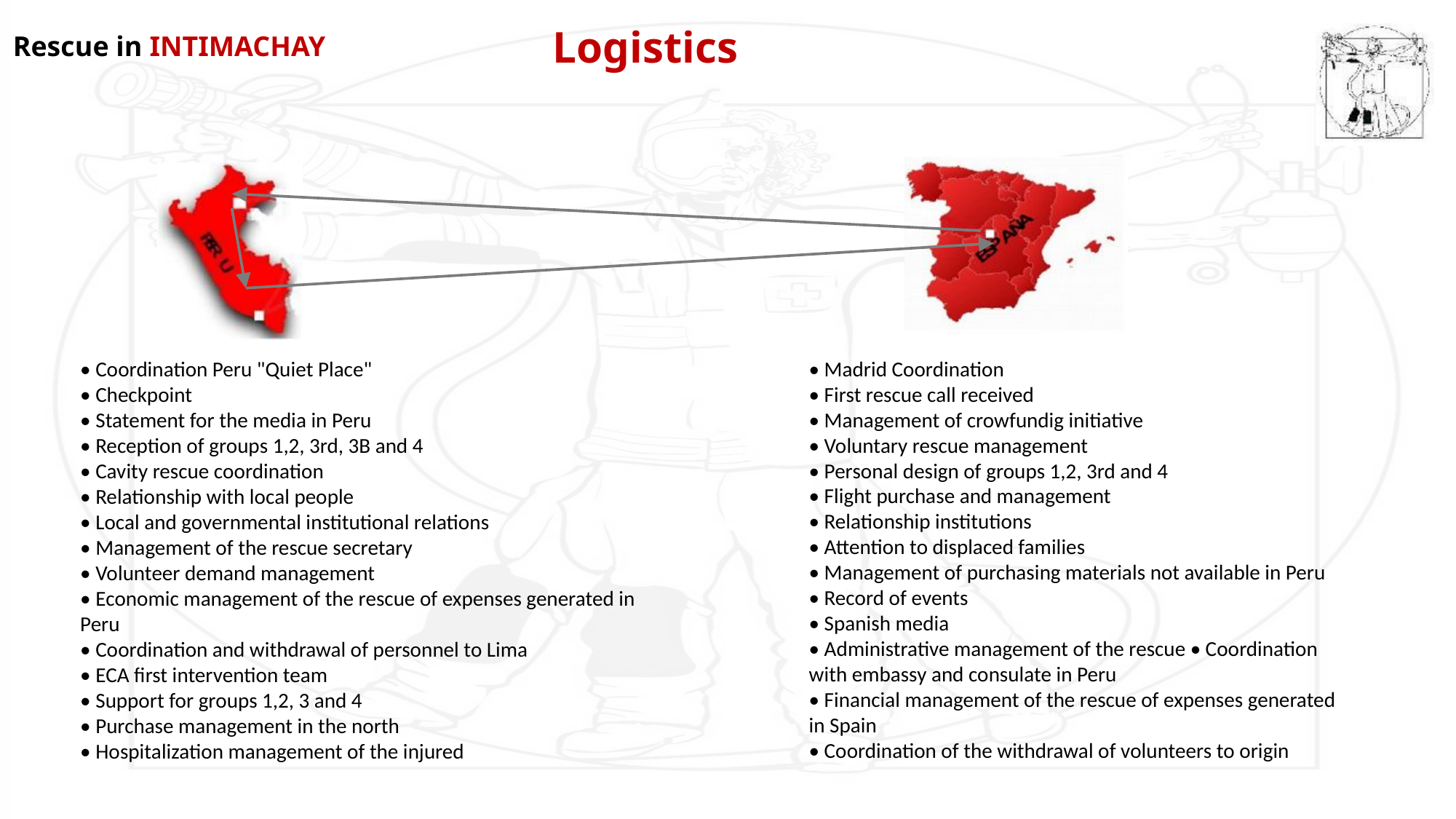

## Slide 45
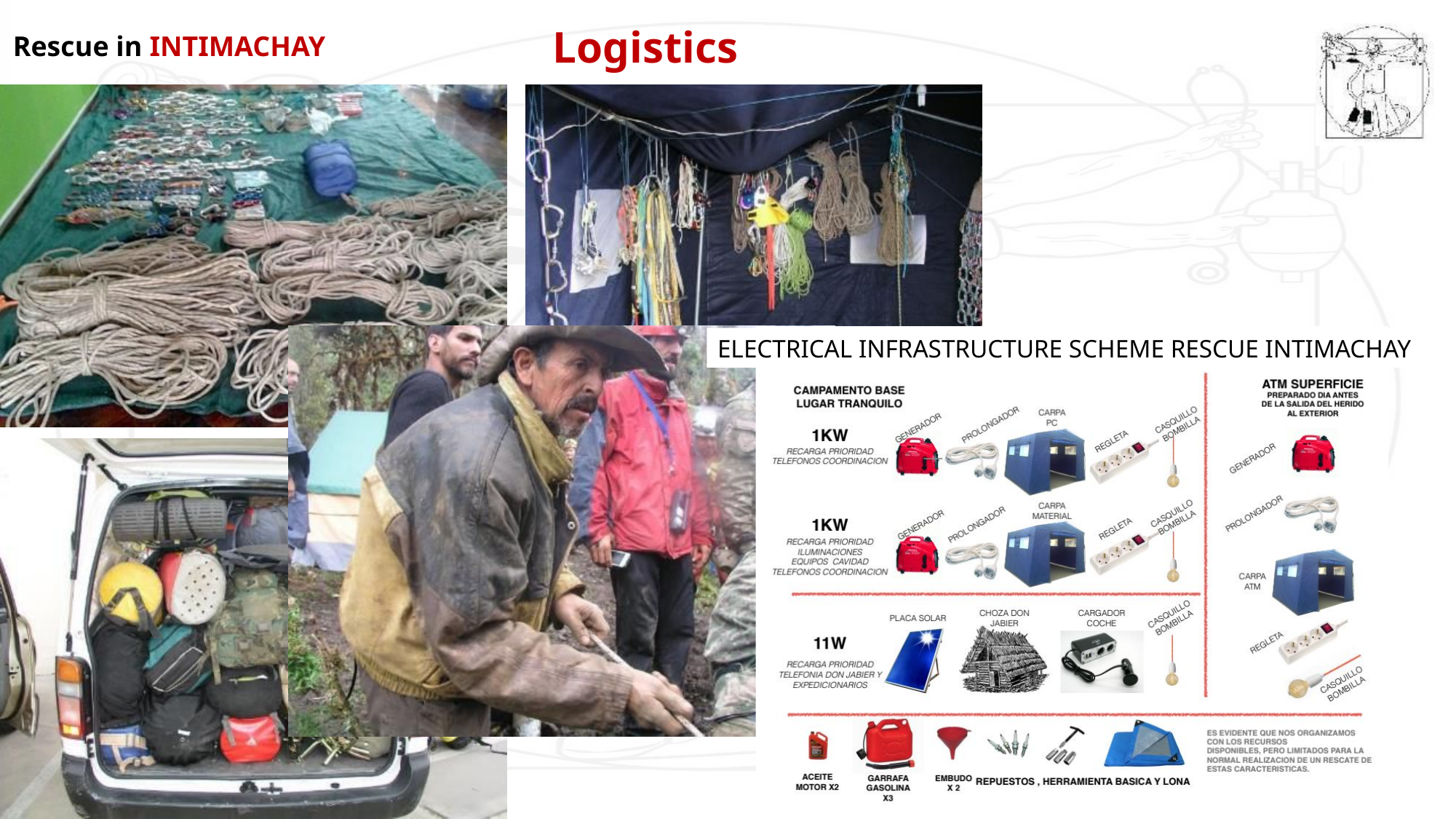

## Slide 46
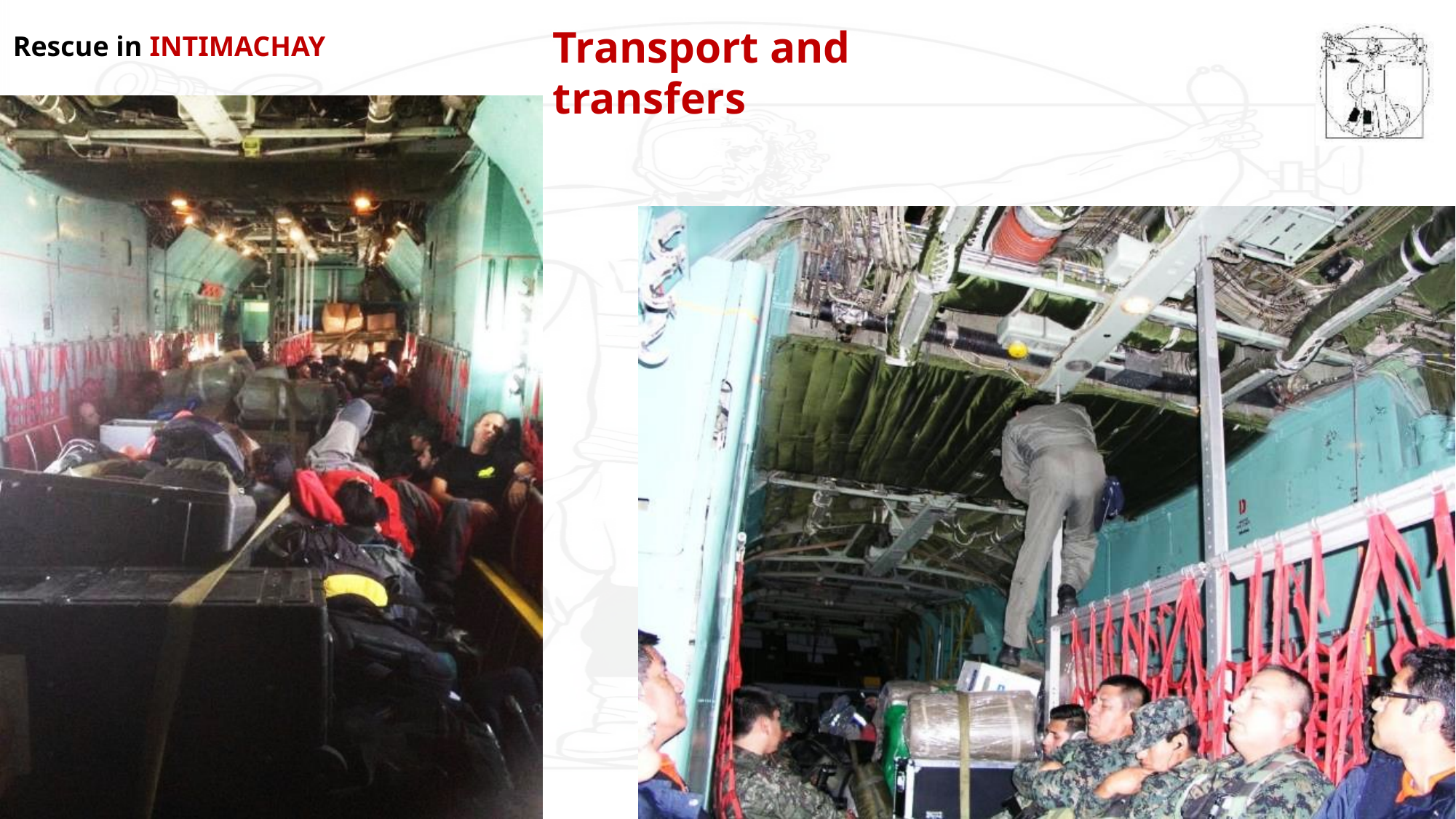

## Slide 47
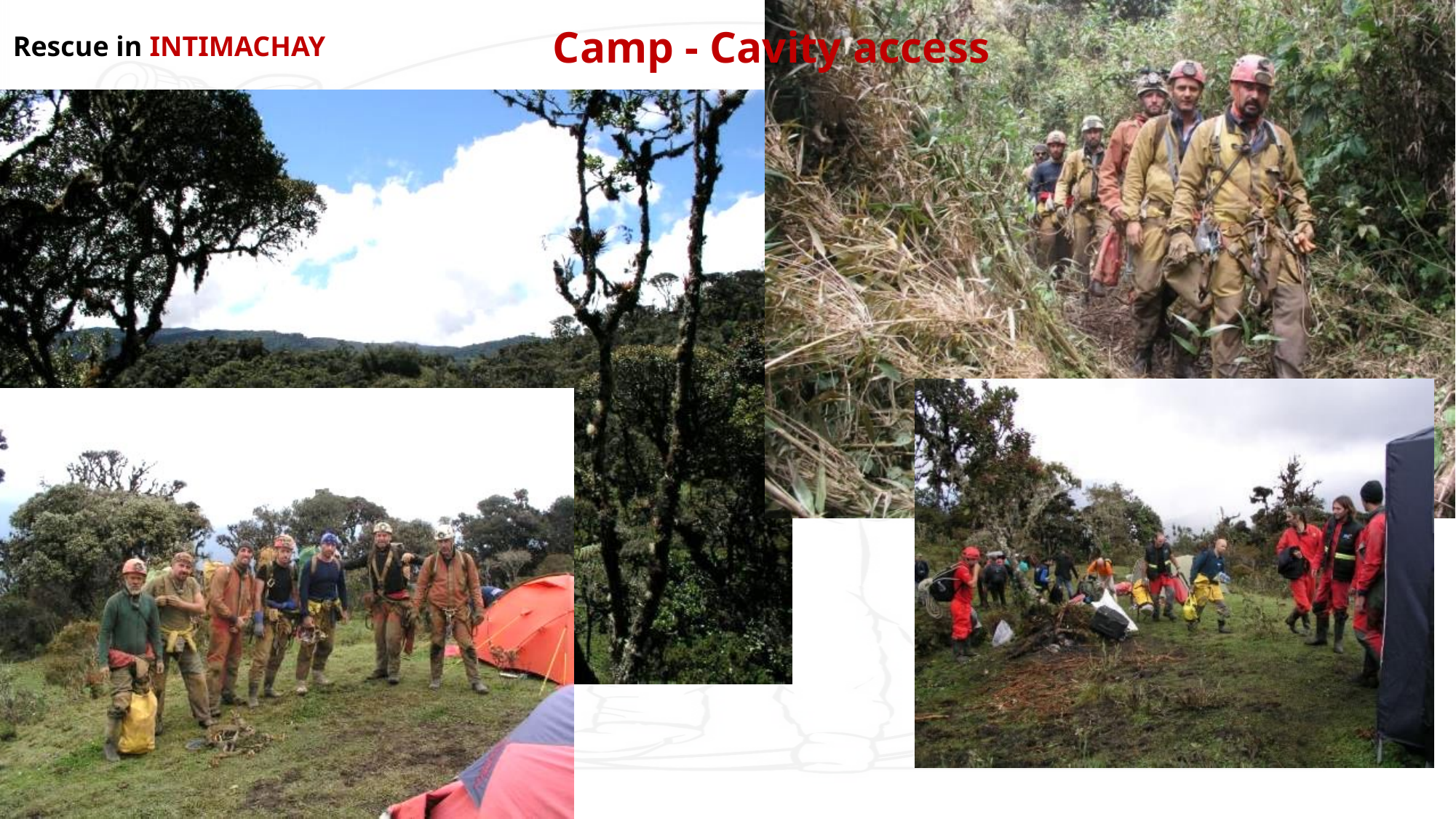

## Slide 48
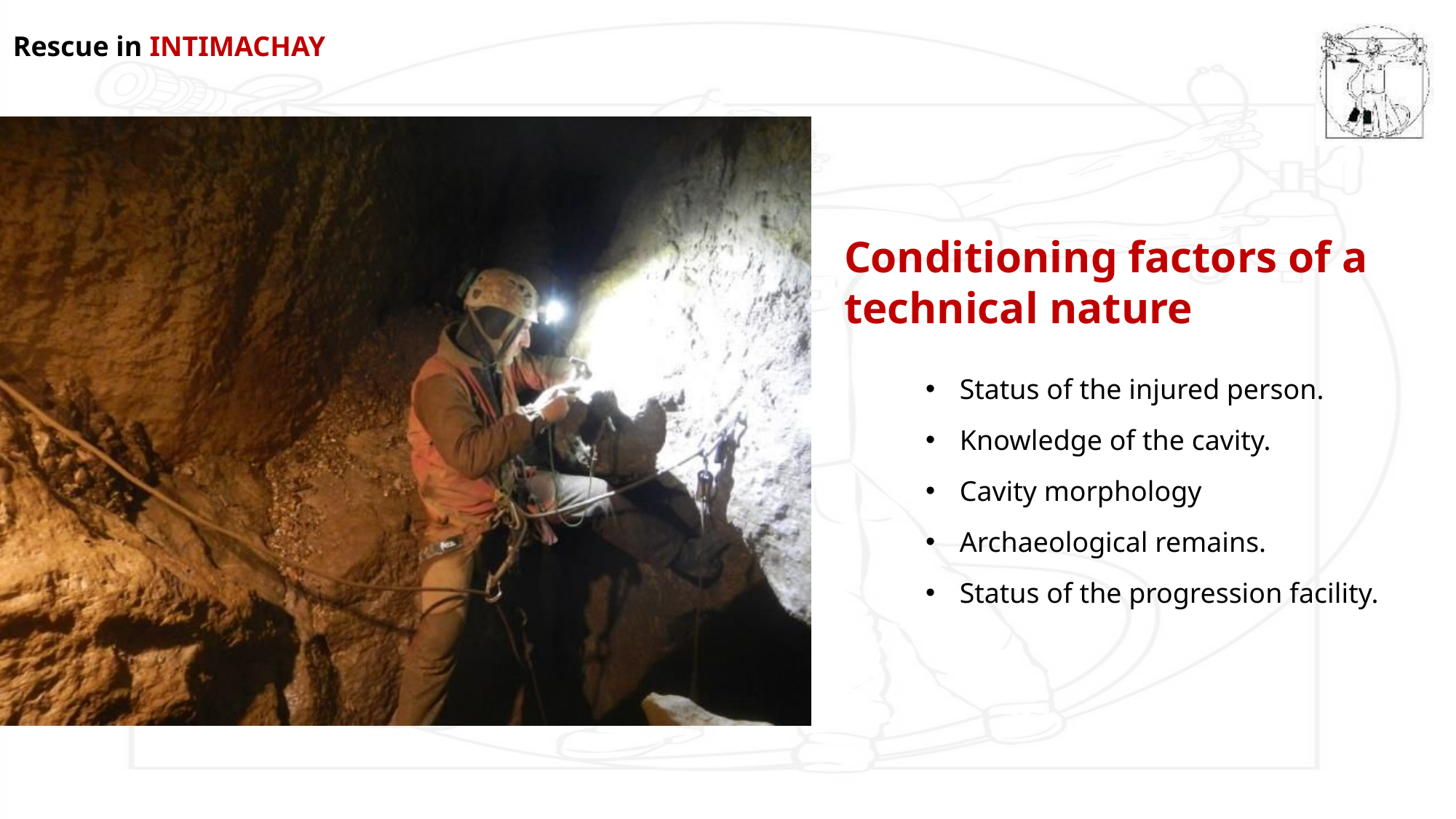

## Slide 49
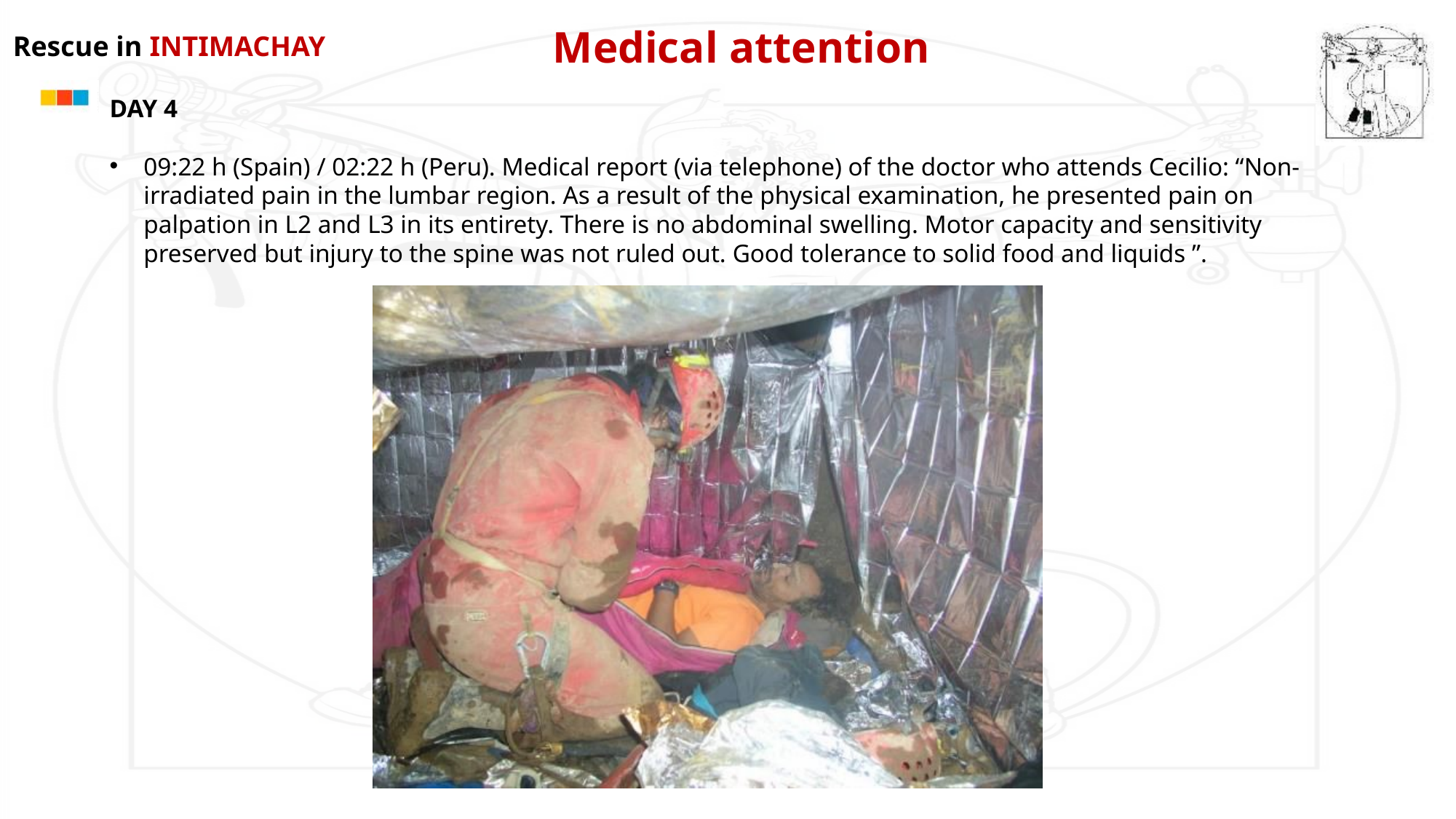

## Slide 50
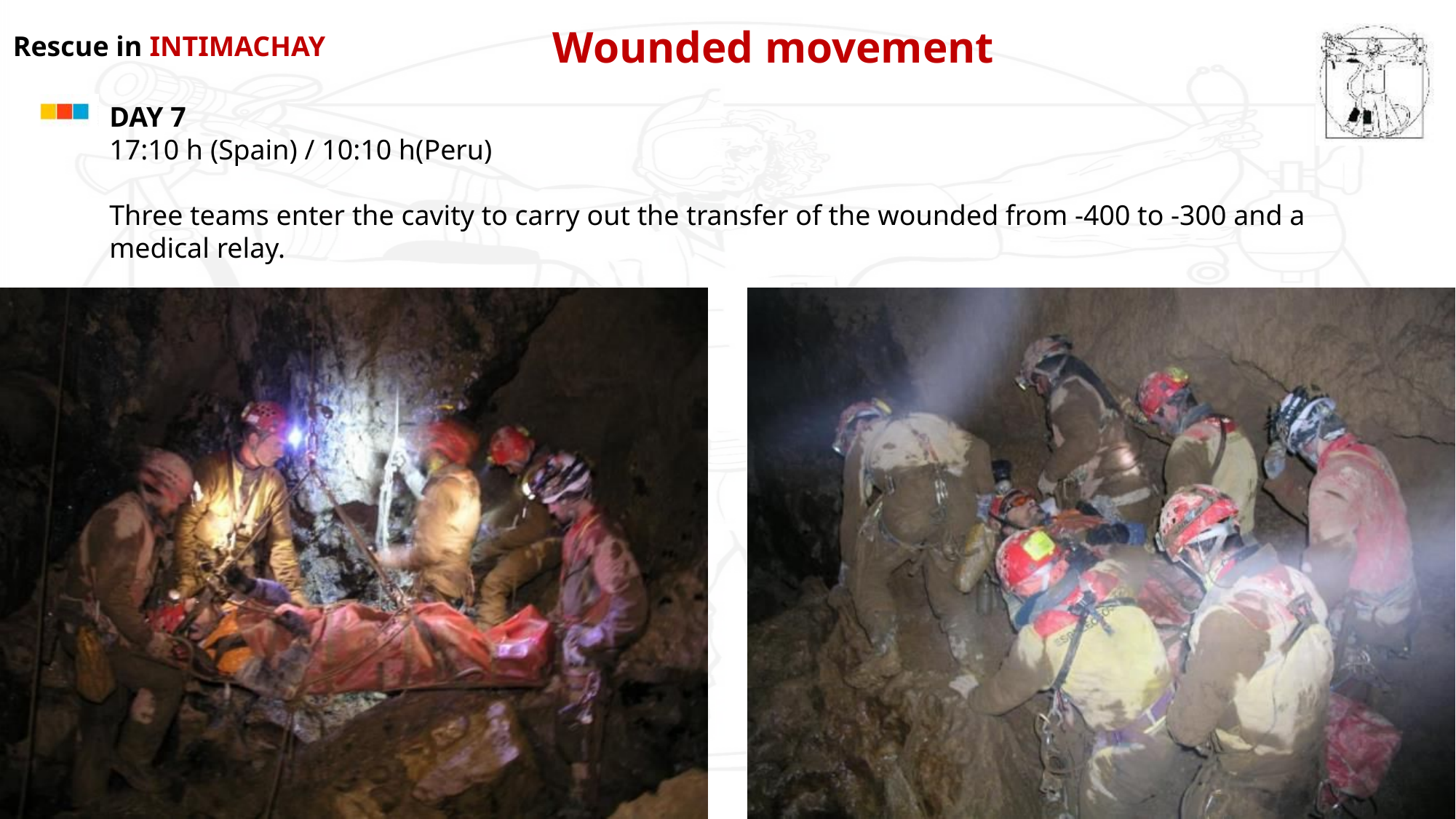

## Slide 51
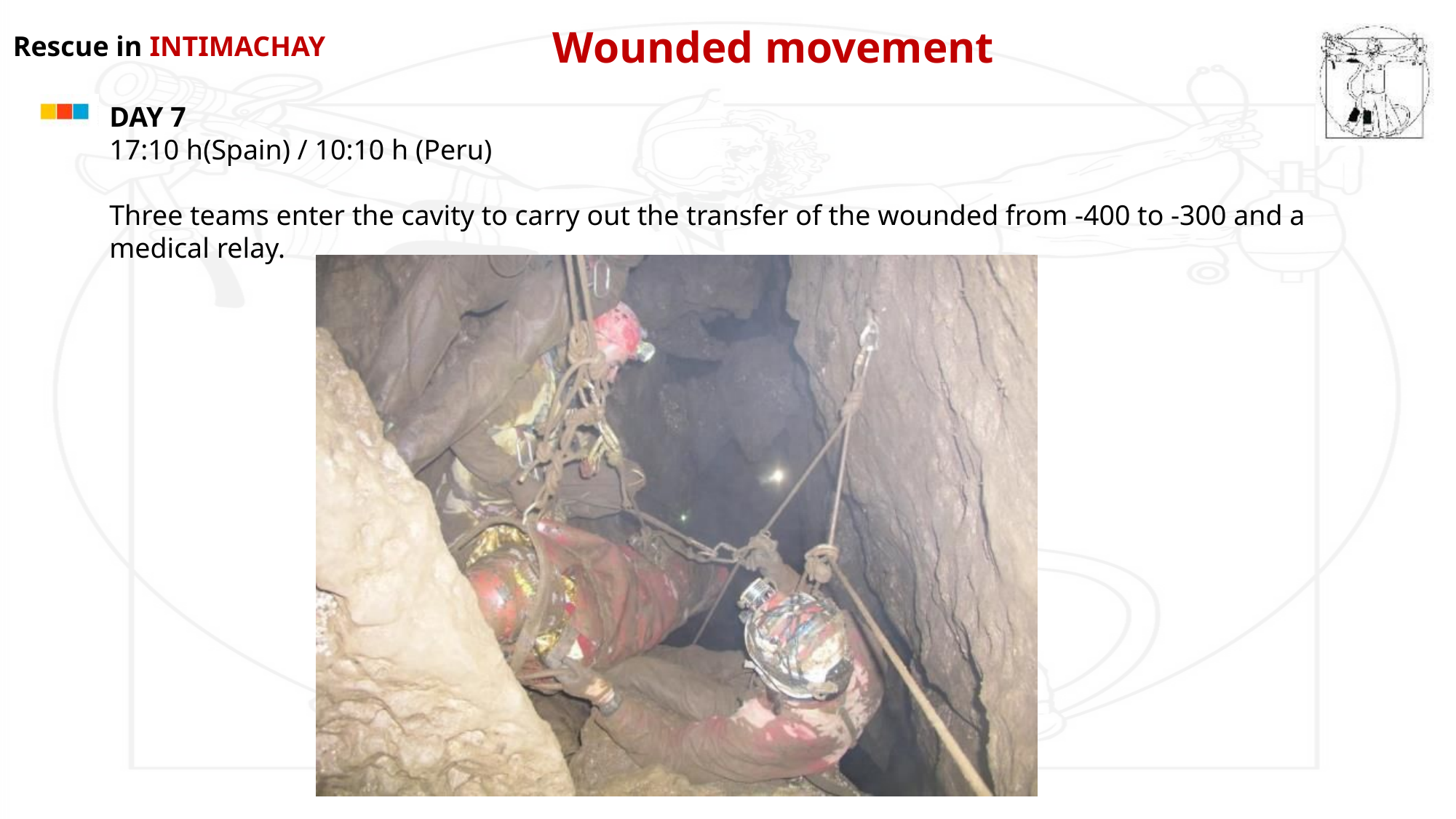

## Slide 52
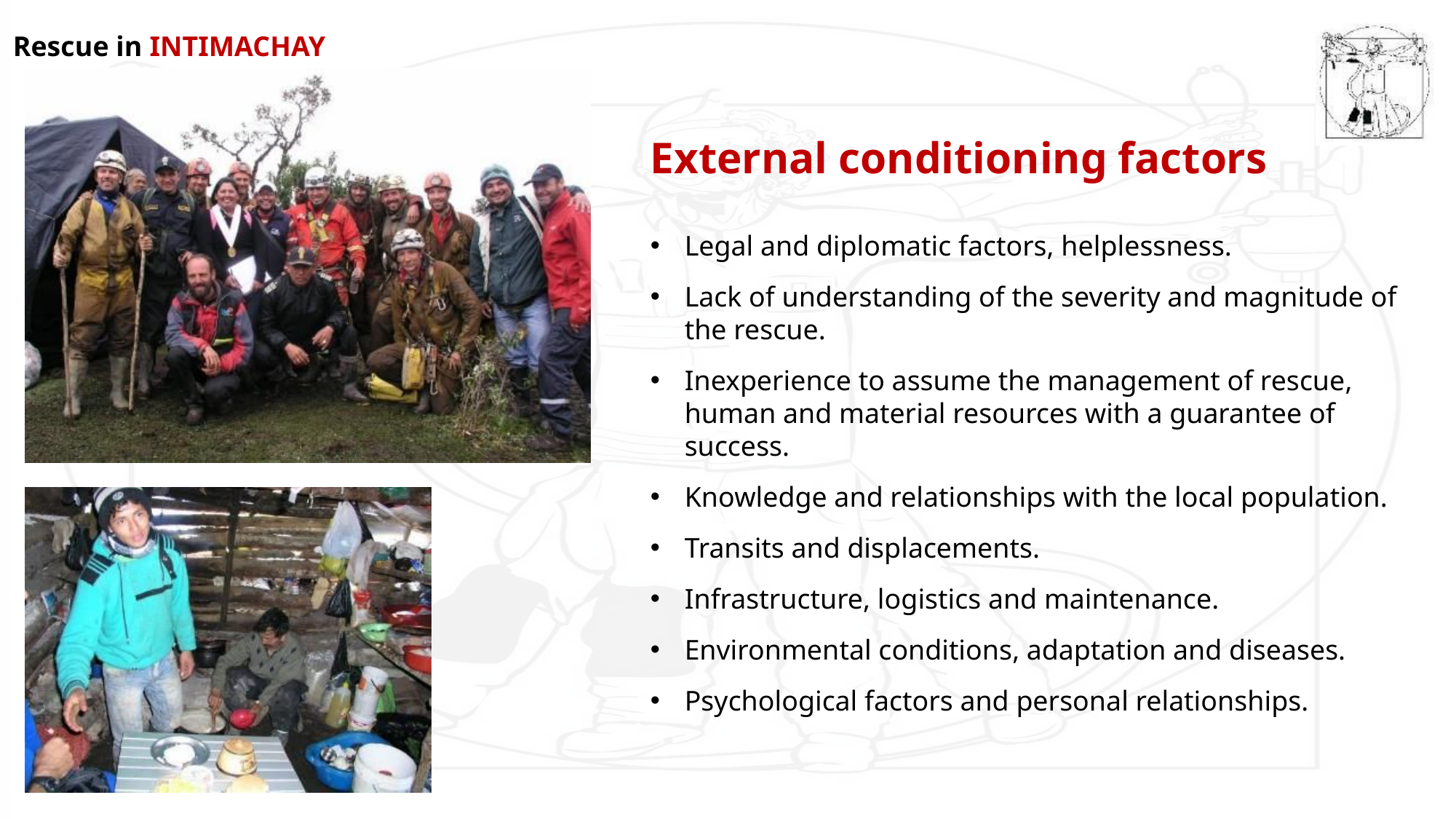

## Slide 53
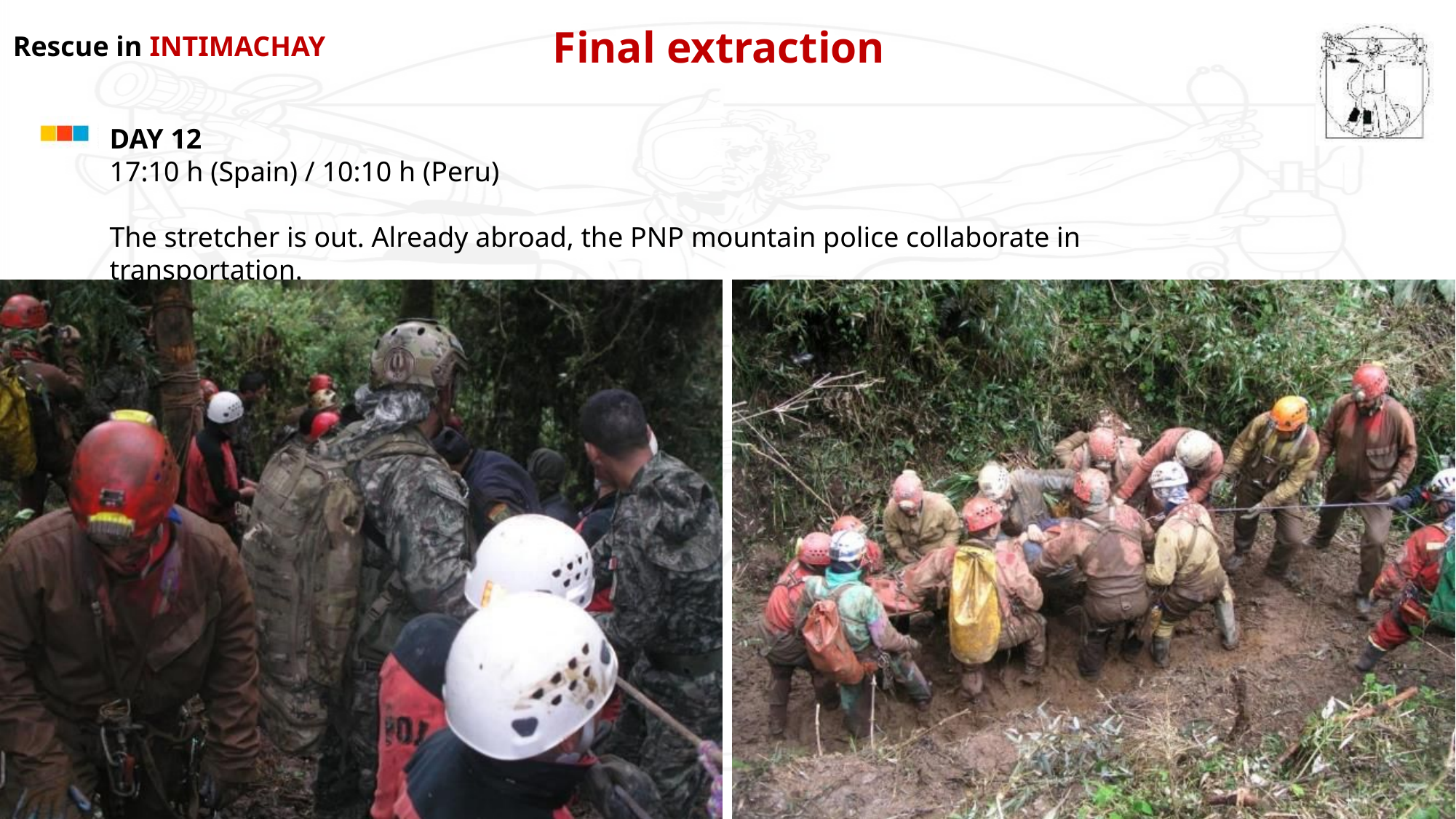

## Slide 54
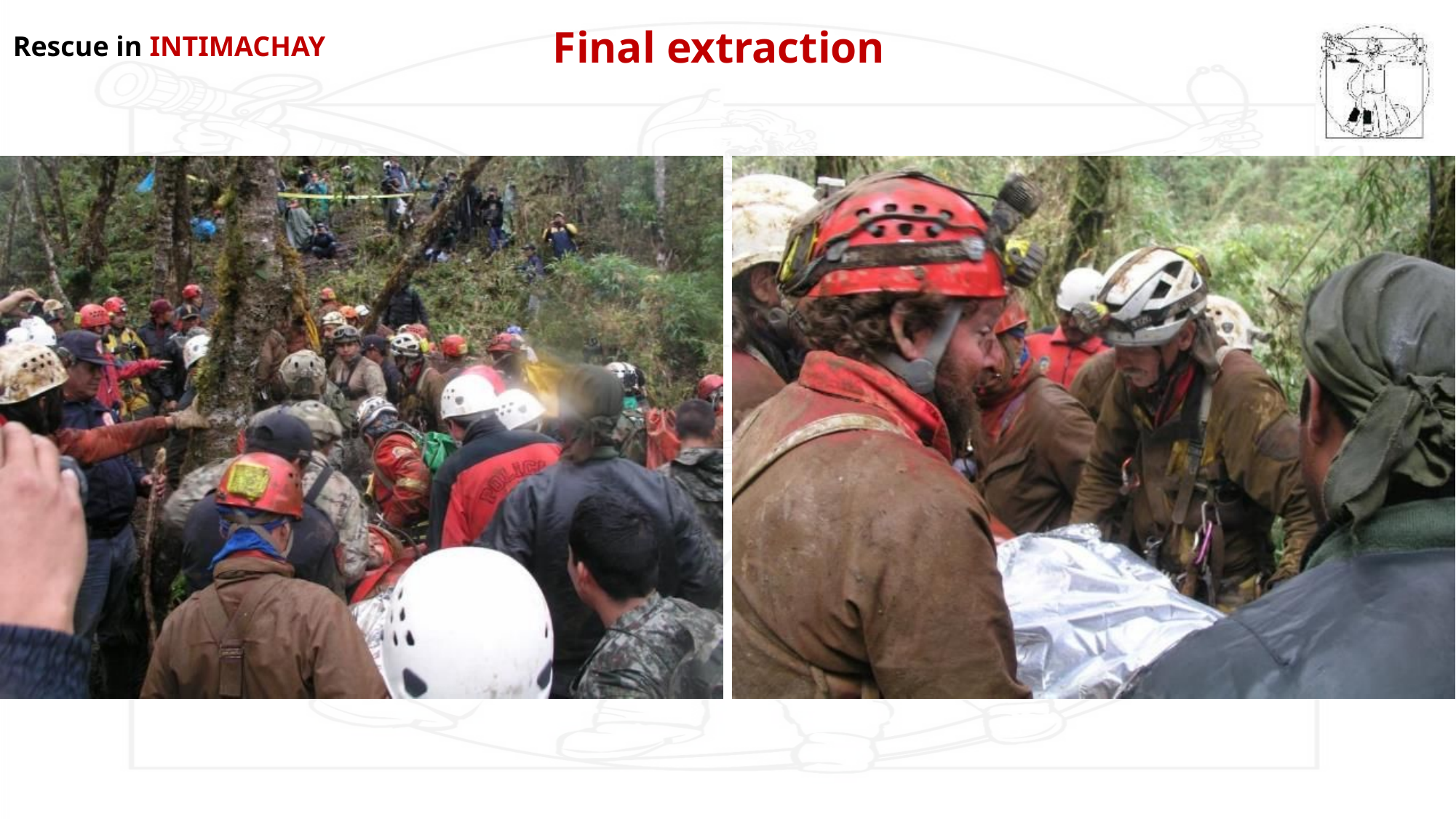

## Slide 55
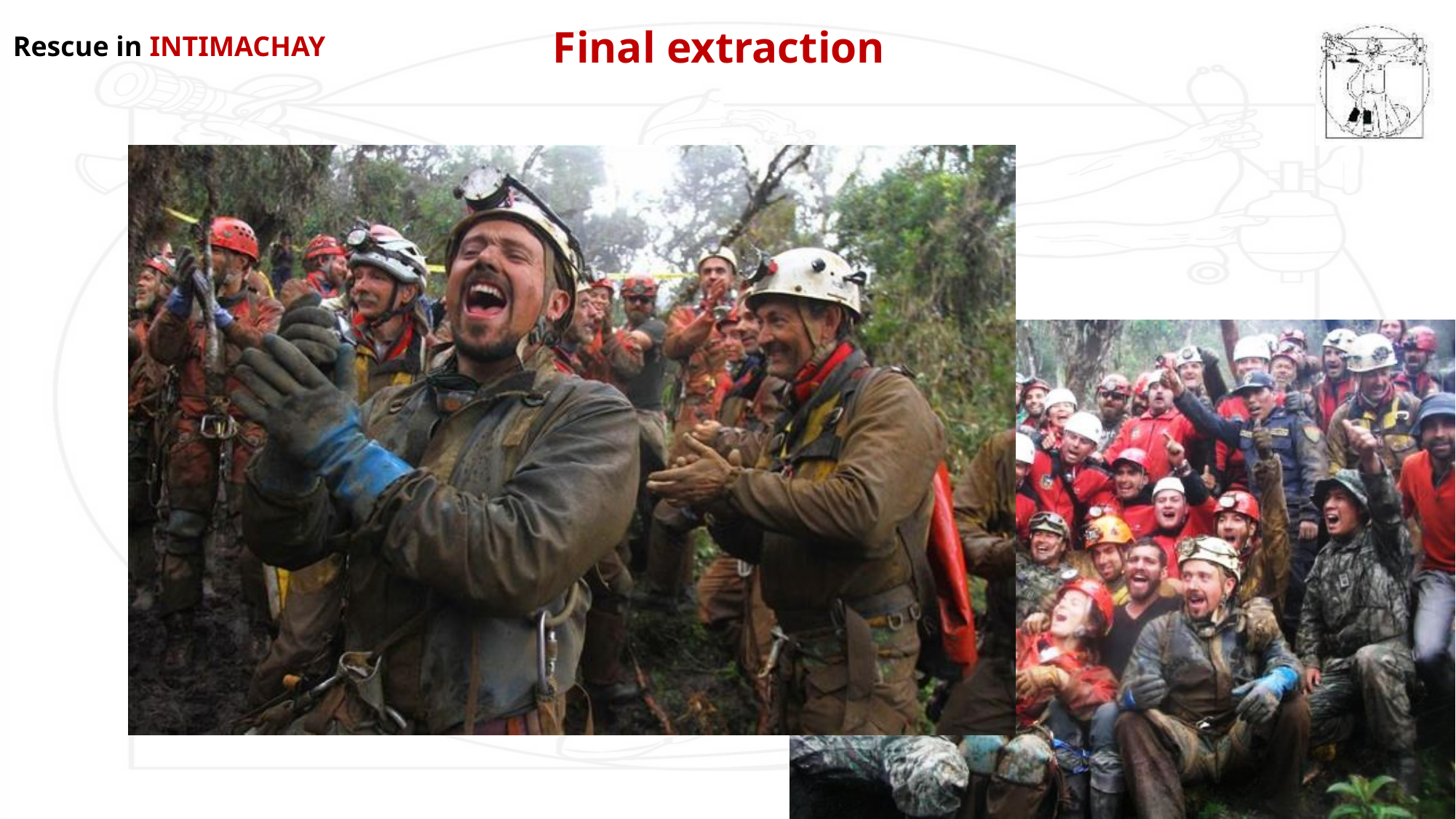

## Slide 56
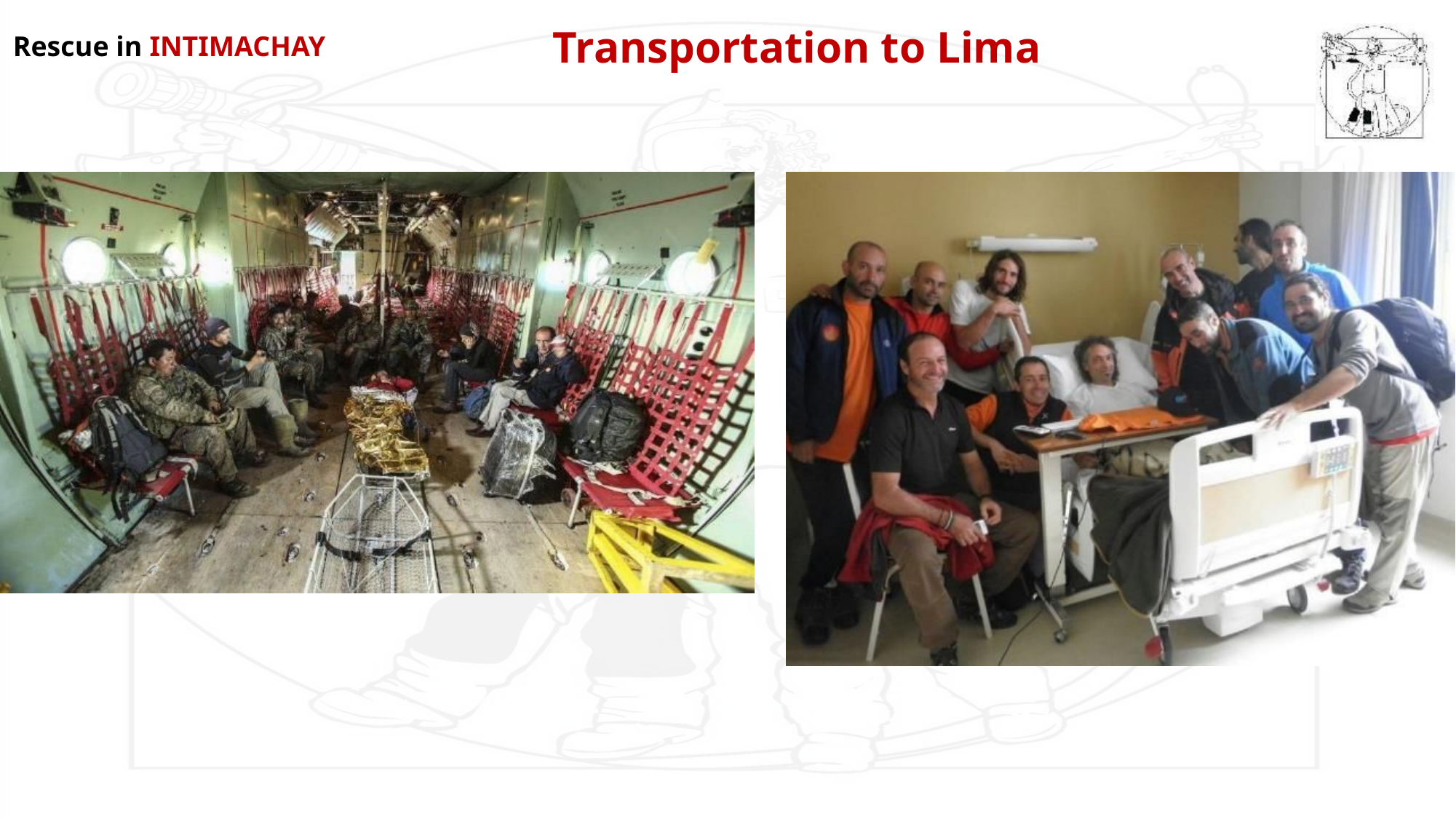

## Slide 57
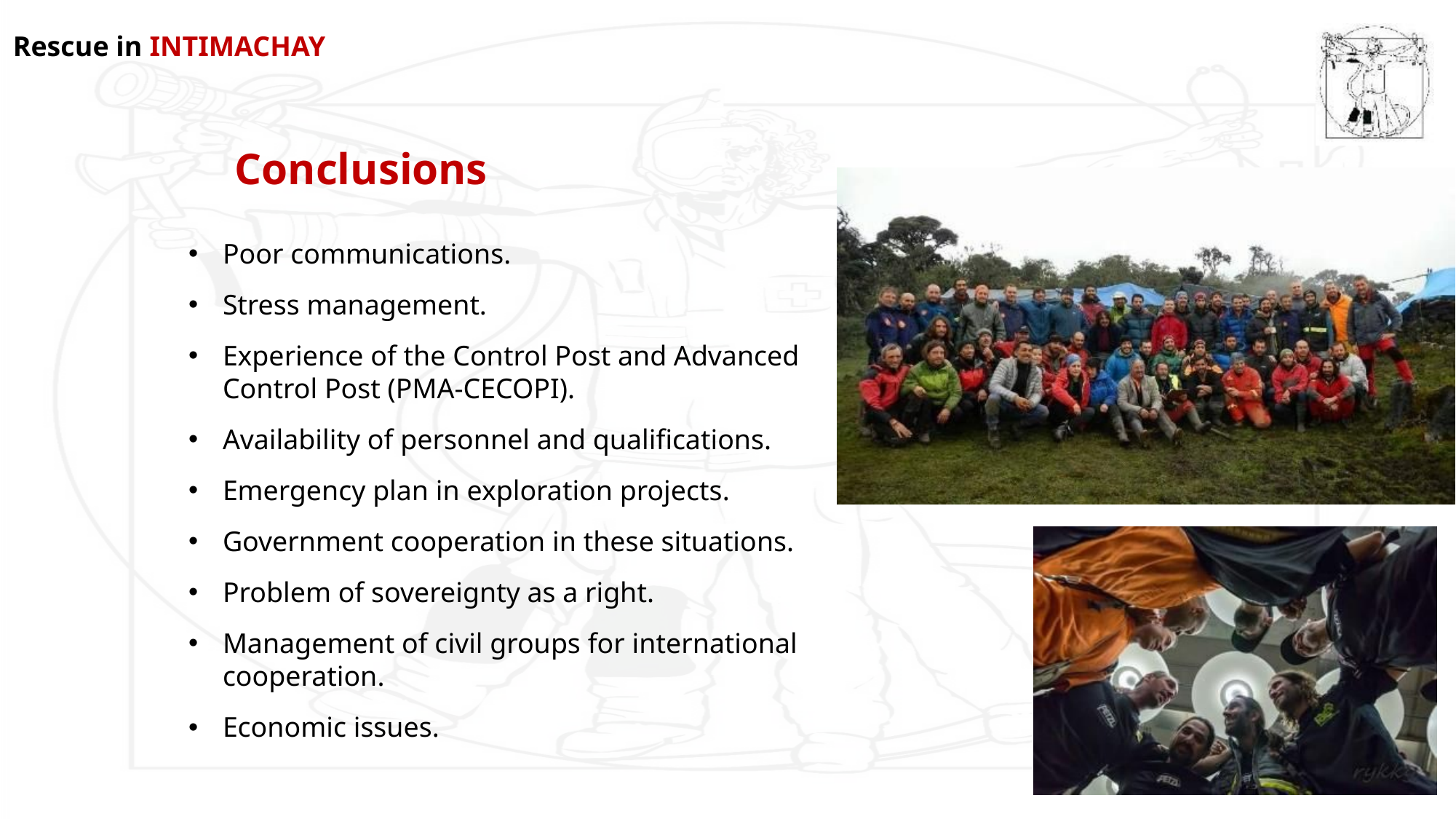

## Slide 58
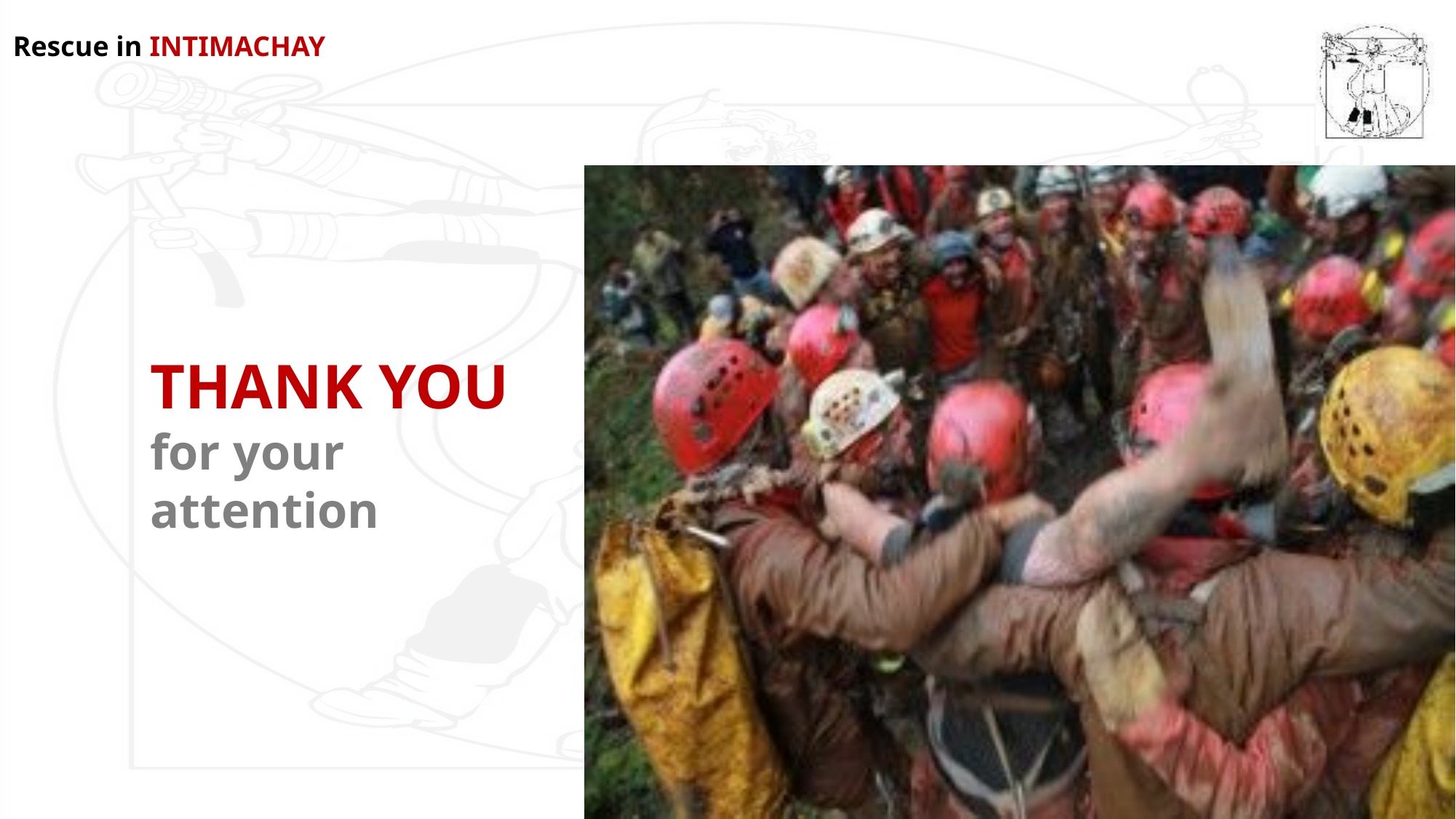

Supplement: Supplementary file 1 [file ijerph-18-03536-s001.zip › PROYECTO UKHUPACHA-PLAN RESCATE INTI MACHAY.english.ppsx]
